# Bi-allelic loss-of-function variants in *PPFIBP1* cause a neurodevelopmental disorder with microcephaly, epilepsy, and periventricular calcifications

**DOI:** 10.1016/j.ajhg.2022.06.008

**Published:** 2022-07-12

**Authors:** Erik Rosenhahn, Thomas J. O’Brien, Maha S. Zaki, Ina Sorge, Dagmar Wieczorek, Kevin Rostasy, Antonio Vitobello, Sophie Nambot, Fowzan S. Alkuraya, Mais O. Hashem, Amal Alhashem, Brahim Tabarki, Abdullah S. Alamri, Ayat H. Al Safar, Dalal K. Bubshait, Nada F. Alahmady, Joseph G. Gleeson, Mohamed S. Abdel-Hamid, Nicole Lesko, Sofia Ygberg, Sandrina P. Correia, Anna Wredenberg, Shahryar Alavi, Seyed M. Seyedhassani, Mahya Ebrahimi Nasab, Haytham Hussien, Tarek E.I. Omar, Ines Harzallah, Renaud Touraine, Homa Tajsharghi, Heba Morsy, Henry Houlden, Mohammad Shahrooei, Maryam Ghavideldarestani, Ghada M.H. Abdel-Salam, Annalaura Torella, Mariateresa Zanobio, Gaetano Terrone, Nicola Brunetti-Pierri, Abdolmajid Omrani, Julia Hentschel, Johannes R. Lemke, Heinrich Sticht, Rami Abou Jamra, Andre E.X. Brown, Reza Maroofian, Konrad Platzer

**Affiliations:** 1 Institute of Human Genetics, University of Leipzig Medical Center, 04103 Leipzig, Germany; 2 MRC London Institute of Medical Sciences, London W12 0NN, UK; 3 Faculty of Medicine, Institute of Clinical Sciences, Imperial College London, London SW7 2AZ, UK; 4 Clinical Genetics Department, Human Genetics and Genome Research Institute, National Research Centre, Cairo 12622, Egypt; 5 Department of Pediatric Radiology, University Hospital Leipzig, 04103 Leipzig, Germany; 6 Institute of Human Genetics, Medical Faculty and University Hospital Düsseldorf, Heinrich-Heine-University Düsseldorf, 40225 Düsseldorf, Germany; 7 Department of Pediatric Neurology, Children’s and Adolescents’ Hospital Datteln, Witten/Herdecke University, 58448 Witten, Germany; 8 UF6254 Innovation en Diagnostic Genomique des Maladies Rares, CHU Dijon Bourgogne, FHU translad, Génétique des Anomalies du Développement, INSERM UMR 1231, Université de Bourgogne-Franche Comté, 21070 Dijon, France; 9 Centre de Génétique et Centre de référence des Maladies rare, Anomalies du Développement et Syndromes Malformatifs, Hôpital d'Enfants, Centre Hospitalier Universitaire de Dijon, 21079 Dijon, France; 10 Department of Translational Genomics, Center for Genomic Medicine, King Faisal Specialist Hospital and Research Center, Riyadh 11211, Saudi Arabia; 11 Department of Anatomy and Cell Biology, College of Medicine, Alfaisal University, Riyadh 11211, Saudi Arabia; 12 Department of Pediatrics, Prince Sultan Military Medical City, Riyadh 12233, Saudi Arabia; 13 Department of Pediatrics, Imam Abdulrahman bin Faisal University, Dammam 34212, Saudi Arabia; 14 Biology Department, Imam Abdulrahman bin Faisal University, Dammam 34212, Saudi Arabia; 15 Department of Neurosciences, University of California, San Diego, La Jolla, CA 92093, USA; 16 Rady Children’s Institute for Genomic Medicine, San Diego, La Jolla, CA 92093, USA; 17 Medical Molecular Genetics Department, Human Genetics and Genome Research Institute, National Research Centre, Cairo 12622, Egypt; 18 Department of Medical Biochemistry and Biophysics, Karolinska Institutet, 171 77 Stockholm, Sweden; 19 Centre for Inherited Metabolic Diseases, Karolinska University Hospital, 171 76 Stockholm, Sweden; 20 Neuropediatric Unit, Department of Women’s and Children’s Health, Karolinska University Hospital, 171 77 Stockholm, Sweden; 21 Department of Molecular Medicine and Surgery, Karolinska Institutet, 171 76 Stockholm, Sweden; 22 Department of Cell and Molecular Biology and Microbiology, Faculty of Biological Science and Technology, University of Isfahan, Isfahan, Iran; 23 Palindrome, Isfahan, Iran; 24 Dr. Seyedhassani Medical Genetic Center, Yazd, Iran; 25 Alexandria University Children’s Hospital, Faculty of Medicine, Alexandria University, Alexandria 21526, Egypt; 26 Clinical, Chromosomal and Molecular Genetics Department, University Hospital Center, 42270 Saint-Étienne, France; 27 School of Health Sciences, Translational Medicine, University of Skövde, 541 28 Skövde, Sweden; 28 UCL Queen Square Institute of Neurology, University College London, London WC1N 3BG, UK; 29 Specialized Immunology Laboratory of Dr. Shahrooei, Sina Medical Complex, Ahvaz, Iran; 30 Department of Microbiology and Immunology, Clinical and Diagnostic Immunology, KU Leuven, 3000 Leuven, Belgium; 31 Department of Precision Medicine, University of Campania Luigi Vanvitelli, 80138 Naples, Italy; 32 Telethon Institute of Genetics and Medicine, 80078 Naples, Italy; 33 Child Neurology Unit, Department of Translational Medical Science, University of Naples Federico II, 80131 Naples, Italy; 34 Department of Translational Medicine, Section of Pediatrics, University of Naples Federico II, 80131 Naples, Italy; 35 Division of Clinical Studies, The Persian Gulf Nuclear Medicine Research Center, Bushehr University of Medical Sciences, Bushehr, Iran; 36 Center for Rare Diseases, University of Leipzig Medical Center, 04103 Leipzig, Germany; 37 Institute of Biochemistry, Friedrich-Alexander-Universität Erlangen-Nürnberg, 91054 Erlangen, Germany

## Abstract

*PPFIBP1* encodes for the liprin-β1 protein, which has been shown to play a role in neuronal outgrowth and synapse formation in *Drosophila melanogaster.* By exome and genome sequencing, we detected nine ultra-rare homozygous loss-of-function variants in 16 individuals from 12 unrelated families. The individuals presented with moderate to profound developmental delay, often refractory early-onset epilepsy, and progressive microcephaly. Further common clinical findings included muscular hyper- and hypotonia, spasticity, failure to thrive and short stature, feeding difficulties, impaired vision, and congenital heart defects. Neuroimaging revealed abnormalities of brain morphology with leukoencephalopathy, ventriculomegaly, cortical abnormalities, and intracranial periventricular calcifications as major features. In a fetus with intracranial calcifications, we identified a rare homozygous missense variant that by structural analysis was predicted to disturb the topology of the SAM domain region that is essential for protein-protein interaction. For further insight into the effects of *PPFIBP1* loss of function, we performed automated behavioral phenotyping of a *Caenorhabditis elegans PPFIBP1/hlb-1* knockout model, which revealed defects in spontaneous and light-induced behavior and confirmed resistance to the acetylcholinesterase inhibitor aldicarb, suggesting a defect in the neuronal presynaptic zone. In conclusion, we establish bi-allelic loss-of-function variants in *PPFIBP1* as a cause of an autosomal recessive severe neurodevelopmental disorder with early-onset epilepsy, microcephaly, and periventricular calcifications.

## Teaser

*PPFIBP1* encodes for liprin-β1, a presynaptic scaffold. Here, we describe 16 individuals from 12 unrelated families with bi-allelic loss-of-function variants in *PPFIBP1* sharing a phenotype of severe developmental delay, epilepsy, microcephaly, and cerebral calcifications. A *C. elegans hlb-1* knockout model demonstrates behavioral abnormalities to underscore gene-disease causality.

## Introduction

*PPFIBP1* (GenBank: NM_003622.4; MIM: 603141) encodes for the PPFIA-binding protein 1, also known as liprin-β1. Liprin-β1 belongs to the liprin protein family whose members are characterized by a highly conserved N-terminal coiled-coil region and three adjacent C-terminal sterile alpha motifs (SAM domains) that form multiple protein-binding surfaces and allow for protein-protein interaction.^1^, ^2^, ^3^ In mammals, the liprin family comprises four liprin-α proteins (liprin-α1–4) and two liprin-β proteins (liprin-β1 and -β2). Liprin-β1 has the ability to homodimerize and to heterodimerize with the homologous α-liprins.^1^ In addition, liprin-β1 and liprin-α1 co-localize to the cell membrane and to the periphery of focal adhesions in fibroblast cell cultures (COS cells).^1^^,^^4^ Liprin-α proteins are major scaffold proteins involved in synapse formation, synaptic signaling, and axonal transport processes via the assembly of large protein complexes.^5^^,^^6^ Although yet to be confirmed, it has been suggested, that liprin-β1 could play a role in the regulation of liprin-α-mediated protein assemblies.^1^^,^^6^^,^^7^ In line with this is the observation that liprin-β1 forms a ternary complex with liprin-α2 and CASK,^3^ a presynaptic scaffolding protein essential for neurodevelopment.^8^^,^^9^ A previous knockout model of the *PPFIBP1* ortholog *hlb-1* in *C. elegans* showed abnormal locomotion behavior. Furthermore, abnormal and decreased distribution of snb-1, an ortholog of human VAMP-family proteins involved in presynaptic vesicle release, increased presynaptic zone size, and resistance to the acetylcholinesterase inhibitor aldicarb indicated a role of *hlb-1* in the regulation of presynaptic function.^10^ Pointing towards a role in neurodevelopment, null-allele mutants of the liprin-β1 ortholog liprin-β resulted in altered axon outgrowth and synapse formation of R7 photoreceptors and also reduced larval neuromuscular junction (NMJ) size in *D. melanogaster*.^7^ Indeed, *PPFIBP1* has been proposed as a candidate gene for congenital microcephaly based on a single family, although this link remains tentative and requires independent confirmation.^11^

Here, we describe a cohort of 16 individuals with a neurodevelopmental disorder from 12 unrelated families harboring homozygous loss-of-function (LoF) variants and a fetus with a missense variant in *PPFIBP1*.

## Subjects and methods

### Patient recruitment and consent

The study was approved by the ethics committee of the University of Leipzig (402/16-ek). Written informed consent for molecular testing and permission for publication of the data was obtained from all individuals and/or their legal representatives by the referring physicians according to the guidelines of the ethics committees and institutional review boards of the respective institute.

All individuals were ascertained in the context of local diagnostic protocols followed by research evaluation of the sequencing data. The compilation of the cohort was supported by international collaboration and online matchmaking via GeneMatcher^12^ in the case of families 2, 4, 5, 6, 7, 8, 9, 10, 11, 12, and 13. Family 3 was recently published as part of a larger cohort of individuals with congenital microcephaly.^11^ In the context of our study, we describe the phenotype of affected individuals of family 3 in detail. Phenotypic and genotypic information were obtained from the referring collaborators with a standardized questionnaire.

### Exome sequencing

Trio exome sequencing (ES) was performed for the affected individuals and the parents in the families 1, 2, 12, and 13 and quadruple ES was done in family 5 for the two affected siblings and their parents. Singleton ES was performed for the affected individuals of the families 3, 4, 6, 8, 9, 10 and 11. Family 7 was investigated by trio genome sequencing (GS) (see supplemental methods for further details).

### Variant prioritization

We first analyzed single-nucleotide variants annotated in local and public mutation databases, as well as rare (minor allele frequency [MAF] < 1% in gnomAD) potentially protein-damaging variants in known disease-associated genes (e.g., by using *in silico* panels like MorbidGenes^13^). Synonymous variants and intronic variants with >20 bp distance to the adjacent exon border were not considered. Variants were prioritized on the basis of the plausibility of the mode of inheritance, zygosity, and phenotype with regard to gene-associated diseases, the presumed consequences on the gene product based on the variant type, allele frequencies in the general population and in-house databases, and *in silico* predicted pathogenicity. Because no pathogenic or likely pathogenic variants according to the guidelines of the American College of Medical Genetics (ACMG)^14^ in known disease-associated genes could be found and the families gave consent for further research, we then evaluated the sequencing data in a research setting aiming to identify potentially causative variants in novel candidate genes. For this purpose, variants in potential candidate genes were prioritized according to the following parameters and an MAF of <1%. The plausibility of the mode of inheritance and zygosity was assessed with respect to the phenotype of parents and siblings and considering parental sequencing data in cases for which trio ES or GS was performed. For further prioritization, we obtained allele frequencies and mutational constraint parameters from gnomAD.^15^ We specifically valued ultra-rare variants (MAF < 0.01%), e.g., heterozygous missense variants in candidate genes with a *Z* score > 3.09 or heterozygous LoF variants in candidate genes with a pLI > 0.9 (pLI = probability of being loss-of-function intolerant) suggesting a selective constraint on these variant types in a population not affected by early-onset neurodevelopmental disorders. For homozygous candidate variants, lower selective constraint measures were used: *Z* score of >0.5 and LOEUF of <0.9 (LOEUF = loss-of-function observed/expected upper bound fraction). In addition, absence of homozygotes in the general population was a requirement. Several *in silico* predictions were considered for missense and splice-site variants (see next paragraph). Candidate genes that are at least moderately expressed in the central nervous system were prioritized, obtaining expression data from GTEx.^16^ All variants in *PPFIBP1* described here were aligned to the human reference genome version GRCh38 (hg38) and to the transcript GenBank: NM_003622.4 (Ensembl: ENST00000228425.11) representing the transcript with the highest expression across all tissues^16^ and the MANE Select v0.95 default transcript.^17^ The pathogenicity of all described variants was classified according to the guidelines of the ACMG^14^ (Table S1).

### *In silico* prediction

*In silico* predictions of the splice-site variant c.1146+1G>A (p.?) were assessed with CADD-v6,^18^ SpliceAI,^19^ MaxEntScan,^20^ and NNSPLICE^21^ (Table S2). *In silico* predictions of the missense variant c.2177G>T (p.Gly726Val) were assessed with CADD-v6,^18^ REVEL,^22^ Mutation Taster,^23^ M-CAP 1.3,^24^ and Polyphen 2^25^ (Table S3).

### Structural analysis

Structural analysis of the p.Gly726Val variant was performed on the basis of the crystal structure of murine PPFIBP1 (PDB: 3TAD, chain C^3^), which exhibits 97% sequence identity to its human ortholog in the region of the SAM domains (according to a BLAST sequence comparison with standard parameters^26^). The exchange was modeled with SwissModel^27^ and RasMol^28^ was used for structure analysis and visualization. For the identification of hydrophobic interactions, we used a 3.9 Å distance cutoff for as specified in Wallace et al., 1995.^29^ Structural analysis for the other variants was not necessary because all are LoF variants and predicted to lead to a loss of protein.

### Mutant *C. elegans* generation

The knockout worm model was designed and made by SunyBiotech (Fuzhou, Fujian, China) in their reference N2 background. CRISPR guide RNA was designed to target a large deletion (17,118 bp) starting close to the start codon and excising all exons from the gene. Deletions were confirmed by PCR.

### Worm preparation

All strains were cultured on Nematode growth medium at 20°C and fed with *E. coli* (OP50) following standard procedure.^30^ Synchronized populations of young adult worms for imaging were cultured by bleaching unsynchronized gravid adults, and allowing L1 diapause progeny to develop for 2.5 days at 20°C.^31^ On the day of imaging, young adults were washed in M9,^32^ transferred to the imaging plates (3 worms per well) with a COPAS 500 Flow Pilot,^33^ and returned to a 20°C incubator for 3.5 h. Plates were then transferred onto the multi-camera tracker for another 30 min to habituate prior to imaging.^34^ For drug experiments, imaging plates were dosed with the compound at the desired concentration 1 day prior to imaging. Worms were then dispensed and tracked as described above, except for the 1 h exposure time where worms were returned to a 20°C incubator for 30 min and then transferred to the tracker for 30 min prior to imaging.^35^

### Image acquisition, processing, and feature extraction

Videos were acquired and processed following methods previously described in detail.^36^ Briefly, videos were acquired in a room with a nominal temperature of 20°C at 25 frames per second and a resolution of 12.4 μm px^−1^. Three videos were taken sequentially: a 5-min pre-stimulus video; a 6-min blue light recording with three 10-s blue light pulses starting at 60, 160, and 260 s; and a 5-min post-stimulus recording.

Videos were segmented and tracked with Tierpsy Tracker.^37^ After segmentation and skeletonization, a manual threshold was applied to filter skeletonized objects, likely to be non-worms from feature extraction, that did not meet the following criteria: 200–2000 μM length, 20–500 μM width. Tierpsy Tracker’s viewer was also used to mark wells with visible contamination, agar damage, or excess liquid as “bad,” and exclude these wells from downstream analysis.

Following tracking, we extracted a previously defined set of 3,076 behavioral features for each well in each of the three videos (pre-stimulus, blue light, and post-stimulus).^38^ The extraction of behavioral features was performed on a per-track basis and are then averaged across tracks to produce a single feature vector for each well. Statistically significant differences in the pre-stimulus, post-stimulus, and blue-light behavioral feature sets extracted from the loss-of-function mutant compared to the N2 reference strain were calculated with block permutation t-tests (code available on GitHub, see web resources). Permutations were randomly shuffled within, but not between, the independent days of image acquisition in order to control for day-to-day variation in the experiments. The p-values were then corrected for multiple comparisons with the Benjamini-Hochberg procedure^39^ to control the false discovery rate at 5%. The code for generating the figures is available on GitHub (see data and code availability).

### Pharyngeal pumping assay

Pharyngeal pumps per minute (ppm) of *C. elegans* strains were determined by counting grinder movements over a 15 s period by eye using a stereomicroscope,^40^ n = 120 worms per strain. Grinder movements of a single worm were counted three times and the results recorded as an average of these values. Statistical differences in ppm between N2 reference strain and *hlb-1(syb4896)* were calculated with block permutation t-tests. The code for generating the figures is available on GitHub (see data and code availability).

## Results

### Clinical description

All individuals except for individual 7 and individual 9 are offspring of consanguineous parents. Four of the 16 individuals in this cohort deceased during childhood at ages ranging from 3 years and 9 months to 8 years. All individuals shared a core phenotype of global developmental delay/intellectual disability (GDD/ID) and epilepsy. 15 were affected by profound or severe GDD/ID (15/16). They had not acquired speech (15/16) and showed impaired motor development (15/16). Most of them never achieved gross motor milestones such as sitting and walking, except for individual 6-1, who was able to sit independently at the age of 6 years, and individual 7, who could stand and walk. Individual 1 presented with moderate ID, developed expressive language skills, and had a normal motor development. All individuals were affected by epilepsy: most commonly with focal seizures (11/16) including focal to bilateral tonic-clonic (2/16) and one case of impaired awareness seizures (1/16). Furthermore, generalized onset seizures occurred in seven of the individuals (7/16). Epileptic spasms were described in seven (7/16). Other reported seizure types included tonic (3/16) and myoclonic seizures (6/16). The median age of seizure onset was at 2 months with a range from the first day of life up to 4 years. Most individuals were initially affected by daily seizures (12/16). All individuals have been treated with multiple antiepileptic drugs (AEDs). In the majority, the epilepsy was drug-resistant (13/16) in that they did not achieve sustained seizure freedom of 1 year or, if longer, three times the longest preintervention interseizure interval on therapy with at least two AED schedules.^41^ One of them, individual 8, was seizure-free for 5 months at the last assessment since the introduction of valproate. The outcome of this intervention remains undetermined. In individuals 7 and 12, epilepsy was drug-responsive (2/16) as they were sustained seizure-free for 2.5 years and 2 years, respectively. Both received antiepileptic polytherapy including valproate. Individual 2 was seizure-free for 4 to 5 months at the last assessment on her first two combined AED regimens and the responsiveness of the seizures remained undefined. (For further information on antiepileptic treatment, see Table S4.) Electroencephalography (EEG) was performed in 14 individuals (Table S4). EEG findings included focal (3/14) or multifocal (5/14) interictal epileptiform discharges. In two individuals, bilateral paroxysmal discharges were observed (2/14). Hypsarrhythmia was recorded in four individuals (4/14) who were also affected by epileptic spasms and thus met the criteria for West syndrome. In one of these cases, the phenotype progressed to Lennox-Gastaut syndrome later. All but individual 8 were affected by microcephaly (15/16), defined here by an occipitofrontal circumference (OFC) ≤ −2 standard deviations (SD) (range: <<−3 SD to −1.78 SD) at last assessment. The majority showed primary (9/16) and/or progressive (11/16) microcephaly. Secondary microcephaly (4/16) developed in individual 1, individual 6-1 (who had a low OFC of −1.94 SD already at birth), individual 7, and individual 12. Individual 8 showed borderline low normal head circumference at the last assessment. Other common neurological findings comprise muscular hypertonia (10/16) up to spastic tetraplegia (6/16), but also muscular hypotonia (5/16), dystonic movements (3/16), and nystagmus (4/16).

Nine individuals were born small for gestational age (birthweight ≤ 10^th^ percentile; 9/16). Failure to thrive leading to decreased body weight (≤−2SD) was seen in eight individuals (8/16), and short stature (height ≤ −2 SD) manifested in seven (7/16). Some of the individuals exhibited feeding difficulties (7/16), and deglutition disorders were described in three of them.

**Figure 1:**
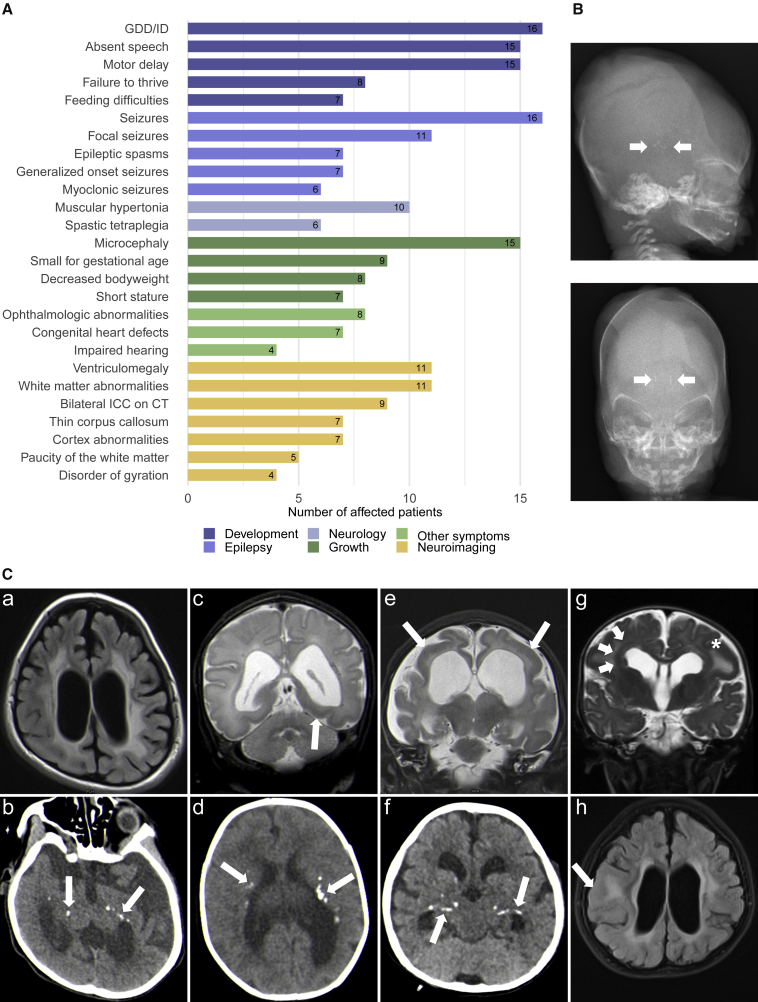
Prevalence of clinical findings, neuroimaging features, and X-ray of the fetus (A) Prevalence of phenotypic features in the cohort grouped by clinical categories. (B) Fetus, 25^th^ gestational week: X-ray babygram postmortem showing macroscopic intracranial calcifications (arrows). (C) Exemplary MRI and CT images. a) Individual 2, MRI, age 5 years, T2-FLAIR axial: pronounced leukoencephalopathy with hyperintensities of the white matter, paucity of the white matter, consecutive ventriculomegaly. b) Individual 2, CT, age 5 years: bilateral symmetrical calcifications periventricular and in the basal ganglia (arrows). c) Individual 3-3, MRI, age 3 days, T2-TSE coronal: moderate ventriculomegaly with accentuation of the occipital horn and pachygyria with thickening of the occipitotemporal cortex (arrow). d) Individual 3-3, CT, age 3 days, bilateral calcifications periventricular and in the deep white matter (arrows). e) Individual 4, MRI, age 3 months, T2-TSE coronal: severe paucity of the white matter and consecutive ventriculomegaly, bilateral pachygyria, and thickening of the parietal cortex (arrows). f) Individual 4, CT, age 3 months: bilateral symmetrical calcifications periventricular and in the basal ganglia (arrows). g) Individual 8, MRI, age 6 months, T2-TSE coronal: ventriculomegaly, pachygyria with thickening of the cortex (asterisk), and periventricular gray matter heterotopia (arrow). h) Individual 12, MRI, age 5 years, T2-FLAIR axial: ventriculomegaly, leukoencephalopathy, paucity of the white matter, and thickening of the cortex (arrow).

Other repeatedly described symptoms include impaired hearing (4/16), ophthalmologic abnormalities (8/16), undescended testes (3/10), and congenital heart defects (7/16). The latter comprise patent ductus arteriosus (PDA, 6/16), atrial septal defects (ASDs, 3/16), ventricular septal defects (VSDs, 2/16), a dilated left ventricle (1/16), and a coronary fistula (1/16) with mitral regurgitation and cardiomegaly. There were no overarching dysmorphic facial features in the affected individuals. (For an overview of the phenotypic spectrum, see Table 1 and Figure 1A. For further details on the phenotype of each individual, see supplemental notes and Table S4.)

**Table 1:** Clinical and genetic details of all affected individuals with causative variants in *PPFIBP1*

**Ind.**	**Age**^a^**(sex)**	**Variant (GenBank:****NM_003622.4****)**	**Development**	**Seizure types (age of onset)**	**MRI (age)**	**ICCs**^b^	**Neurological findings**	**Microcephaly**	**Growth**	**CHD**	**Ophthalmologic features**
**Ind. 1**	19 years (M)	c.1146+1G>A (p.?), homozygous	moderate ID, delayed speech, normal motor development	focal impaired awareness (4 years)	normal (18 years)	not done	none	yes	SGA	no	normal
**Ind. 2**	6 years^c^ (6 years) (F)	c.2654del (p.Tyr885Leufs^∗^4), homozygous	profound DD, no speech, unable to sit	focal, generalized tonic clonic (2 months)	paucity of the WM, VM, hypoplastic CC, Blakes's pouch cyst (5 years)	yes	spastic tetraplegia, nystagmus	yes	short stature, low weight	no	bilateral papillary pallor, no eye contact
**Ind. 3-1**	11 years (M)	c.1368_1369del (p.Glu456Aspfs^∗^3), homozygous	profound DD, no speech, unable to sit	epileptic spasms, focal, tonic clonic, tonic (7 months)	periventricular leukomalacia, metopic synostosis	yes	spastic tetraplegia	yes	SGA, low weight	yes	poor fixation
**Ind. 3-2**	7 years (M)	c.1368_1369del (p.Glu456Aspfs^∗^3), homozygous	profound DD, no speech, unable to sit	epileptic spasms, LGS (2 months)	moderate hyperintensity of periventricular white matter, mild VM (2 years)	yes	spastic tetraplegia	yes	SGA, low weight	yes	normal
**Ind. 3-3**	5 years (M)	c.1368_1369del (p.Glu456Aspfs^∗^3), homozygous	profound DD, no speech, unable to sit	epileptic spasms, focal, multifocal (1 day)	VM, abnormal signal intensity of the WM, bilateral temporal and left occipital pachygyria (3 days)	yes	spastic tetraplegia	yes	SGA, low weight	yes	normal
**Ind. 4**	11 months (M)	c.1368_1369del (p.Glu456Aspfs^∗^3), homozygous	profound DD no speech, unable to sit	epileptic spasms, focal, generalized tonic, status epilepticus (5 months)	VM, paucity of the WM, bilateral parietal and occipital pachygyria (5 months)	yes	spastic diplegia, hyperreflexia	yes	SGA, short stature	yes	haemorrhagic retinitis, chronic retinal detachment, right eye exotropia w/ slow pupillary reaction
**Ind. 5-1**	8 years^c^ (8 years) (F)	c.2413C>T (p.Arg805^∗^), homozygous	profound DD, no speech, unable to sit	focal, myoclonic (2 months)	not done	yes	spastic tetraplegia	yes	SGA, short stature, low weight	no	N/A
**Ind. 5-2**	2 years^c^ (4 years) (F)	c.2413C>T (p.Arg805^∗^), homozygous	profound DD, no speech, unable to sit	focal, myoclonic, tonic (1 months)	not done, VM on CT	yes	hypertonia of the limbs, dystonia	yes	short stature	no	normal
**Ind. 6-1**	6 years (M)	c.1468C>T (p.Gln490^∗^), homozygous	profound DD, no speech, sat independently at 6 years	generalized tonic clonic, myoclonic (4 months)	VM, cortical atrophy, demyelination of periventricular WM, thin CC, cerebellar vermian hypoplasia	not done	hypertonia of the limbs	yes	short stature, low weight	no	optic atrophy, followed light
**Ind. 6-2**	2 years (M)	c.1468C>T (p.Gln490^∗^), homozygous	profound DD, no speech, no head support	generalized tonic clonic, myoclonic, excessive smacking movements (2 months)	asymmetrical VM, cortical atrophy, demyelination of periventricular WM, thin CC, cerebellar vermian hypoplasia	yes	spasticity, rigidity, dystonic movement	yes	short stature, low weight	yes	optic atrophy, couldn't follow light
**Ind. 7**	4 years (F)	c.403C>T (p.Arg135^∗^), homozygous	severe DD, no speech, motor delay but can stand and walk	epileptic spasms, focal with apnoea, myoclonic (4 months)	normal at 4 months; thin CC, periventricular dysmyelination, possibly reduction of the WM at 1.5 years	not done	hypotonia	yes	normal	no	normal
**Ind. 8**	N/A (F)	c.1417_1427del (p.Ala473Lysfs^∗^20), homozygous	profound DD, no speech, unable to sit	generalized tonic clonic (6 months)	bilateral parietal pachygyria, periventricular heterotopia, VM, hyperintensity and paucity of the WM	N/A	hypotonia, nystagmus	no, but low OFC	SGA	yes	normal, but poor fixation
**Ind. 9**	2 years 6 months^c^ (3 years 9 months) (M)	c.1300C>T (p.Gln434^∗^), homozygous	severe DD, no speech, can sit but not walk	epileptic spasms and gaze (2 months)	abnormal	N/A	spastic tetraplegia, no sphincter control	yes	SGA	no	blindness
**Ind. 10**	1 years 2 month (M)	c.2629C>T (p.Arg877^∗^), homozygous	severe DD, no speech yet, motor delay	focal myoclonic, epileptic spasms (1 week)	abnormal myelination of the periventricular WM and at corona radiata and centrum semiovale, hypoplastic CC, mild VM	N/A	hypotonia, nystagmus	yes	N/A	no	right ptosis, left iris coloboma, diffuse chorioretinal degeneration
**Ind. 11**	5 months (M)	c.1468C>T (p.Gln490^∗^), homozygous	severe DD, no speech yet, no head support	focal, myoclonic (2 weeks)	cortical atrophy, deep Sylvian fissures, mild VM, prominent basal ganglia, hypoplastic CC, retrocerebellar and bitemporal arachnoid cysts	yes	hypotonia, dystonia, brisk reflexes, nystagmus	yes	SGA	yes	optic atrophy
**Ind. 12**	5 years 11 months (F)	c.2654del (p.Tyr885Leufs^∗^4), homozygous	profound DD, no speech, unable to sit	focal, generalized (6 months)	VM, leukoencephalopathy, paucity of the WM, suspected periventricular microcalcifications, frontal polymicrogyria, temporoparietal thickening of the cortex (5 years)	not done	hypotonia, dyskinesia, stereotypic movements	yes	short stature, low weight	N/A	abnormalities of VEPs
**Fetus (ind. 13)**	25^th^ GW	c.2177G>T (p.Gly726Val), homozygous	developmental age estimated around 22^nd^ GW	–	–	yes^d^	–	yes	IUGR	–	–

Abbreviations: CC, corpus callosum; CHD, congenital heart defect; DD, developmental delay; F, female; IUGR, intrauterine growth restriction; GDD, global developmental delay; GW, gestational week; ICCs, intracranial calcifications; ID, intellectual disability; LGS, Lennox-Gastaut syndrome; M, male; N/A, not available; OFC, occipitofrontal circumference; SGA, small for gestational age; VEPs, visually evoked potentials; VM, ventriculomegaly; WM, white matter.

Further clinical details are provided in Table S4.

a Age at last assessment.

b On CT scan.

c Deceased (age at death).

d ICCs seen on X-ray babygram.

### Neuroimaging

Neuroimaging revealed abnormalities of brain morphology in all 14 individuals that underwent MRI, except for individual 1, who had a normal MRI at the age of 18 years. Eleven individuals presented signs of leukoencephalopathy (11/14) (Figure 1C) mainly in a periventricular localization (9/14). Five of the individuals showed paucity of the white matter (5/14). For each of the individuals 3-2 and 4, MRI data from two different time points was available that suggested a progression of the periventricular hyperintensities and loss of white matter, respectively. Seven individuals had abnormalities of the cortex morphology (7/14). Four of them showed disorders of cortical gyration (Figure 1C) including bilateral frontal polymicrogyria (1/14), increased cortical thickness (4/14), and pachygyria (3/14), with one also showing severe periventricular gray matter heterotopia (1/14; Figure 1C: [g]). Cortical atrophy was seen in three (3/14). Ventriculomegaly of variable degree (10/14) was a common finding. Other notable findings included hypoplasia of the corpus callosum (7/14), cerebellar vermian hypoplasia (2/14), and a Blake’s pouch cyst (1/14). Head CT scan, performed in eight individuals, revealed bilateral intracranial calcifications (ICCs) in all of them (9/9). Calcifications mostly appeared in a scattered pattern with periventricular localization (9/9) but also the basal ganglia (5/9), centra semiovale (2/9), and internal capsule (2/9) were affected (Figure 1C). Furthermore, CT scan also showed ventriculomegaly in individual 5-2, who did not have MRI.

### Fetal phenotype

The fetus (individual 13) showed severe intrauterine growth retardation and microcephaly during pregnancy, and the pregnancy was terminated in the 25^th^ gestational week. Autopsy confirmed length and weight below −2 SD and an occipitofrontal circumference below −4 SD. An X-ray babygram showed ICCs (Figure 1B), and the histopathological examination of the brain revealed predominant macrocalcification and rare necrotic foci in the process of calcification in the germinative and periventricular areas around the 3^rd^ ventricle and occipital horns, as well as cerebral edema with spongiosis and glial response. As an additional finding, autopsy revealed a bicornuate uterus. The parents of the fetus are healthy individuals.

### Genetic results

ES and GS revealed homozygous LoF variants in *PPFIBP1* (GenBank: NM_003622.4) in all affected individuals. In the affected individuals of the families 2-12, we detected eight different homozygous protein-truncating variants. These comprise five nonsense variants and three frameshift variants. Three of the variants were recurrent as each was identified in two unrelated families. (All variants are displayed in Table 1.) Because all of these variants lead to premature termination codons > 50 nucleotides upstream of the last exon-exon splice junction considering the transcripts with the highest expression overall and in brain tissues in specific (GenBank: NM_003622 and GenBank: NM_001198915.2),^15^^,^^16^ they are predicted to undergo nonsense-mediated mRNA decay (NMD).^42^ For two of the variants described above, limitations to the prediction of NMD have to be considered. For the variant c.403C>T (p.Arg135^∗^), NMD can only be predicted (Figure S2) with respect to transcript GenBank: NM_003622.4, as the variant lies in the 5′ untranslated region (UTR) of the transcript Genbank: NM_001198915.2. The variant c.1300C>T (p.Gln434^∗^) in exon 15/30 (GenBank: NM_003622.4) is predicted by SpliceAI^19^ to cause a loss of the acceptor- and donor-splice sites of exon 15 with Δ-scores of 0.39 and 0.3, respectively, which means the variant could affect splicing at these positions. A loss of these splice sites would lead to an in-frame deletion of exon 15, which would potentially be less disruptive on protein function than NMD due to a nonsense variant. All LoF variants mentioned above can be classified as pathogenic according to the guidelines of the ACMG^14^ except for the variant c.1300C>T (p.Gln434^∗^), which can only be classified as of unknown significance. Nonetheless, this variant is deemed causative due to a high phenotypic overlap with the rest of the cohort (Table S1).

In family 1, a homozygous splice-site variant, c.1146+1G>A (p.?), affecting the consensus 5′-splice site of exon 13 was identified. Multiple *in silico* tools consistently predict a loss of the splice site (Table S2). This could lead to out-of-frame exon skipping or to intron retention.^43^^,^^44^ Thus, the mRNA resulting from this allele is likely to include a premature termination codon, thus resulting in NMD (Figure S1).

**Figure 2:**
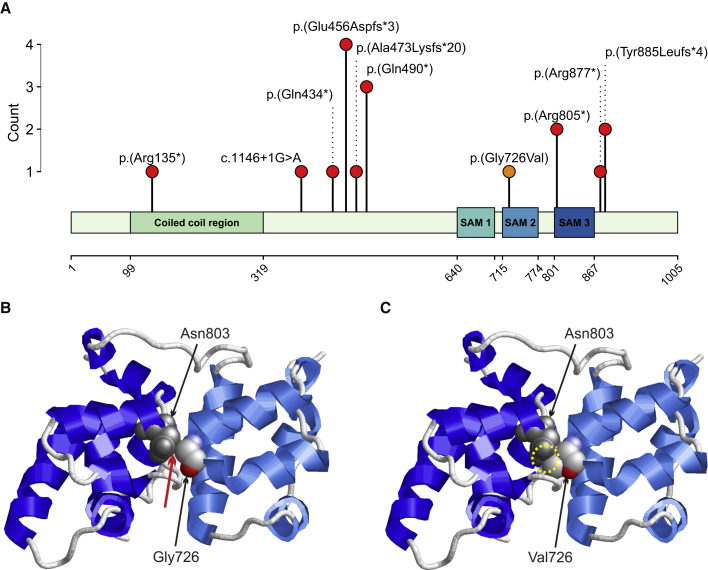
Variant locations on protein level and structure of liprin-β1 illustrating the effect of the p.Gly726Val exchange (A) Location of the variants on protein level aligned to the liprin-β1 isoform 1 (GenBank: NP_003613.4 [GenBank: NM_003622.4]) and count of each variant in the cohort. Truncating and splice-site variants are indicated by red dots, the missense variant is indicated by an orange dot. Abbreviation: SAM, sterile alpha motif. (B) Structure of SAM2 and SAM3 domains that pair in the wild-type protein. Gly726 is located in a tight turn and makes interactions with Asn803 of the adjacent SAM domain. The contacts are formed between Gly732(Cα) and Asn803(Cβ) (distance = 3.5 Å) and between Gly732(Cα) and Asn803(Cγ) (distance = 3.6 Å). The site of the contacts is marked by a red arrow; Asn803 is shown in grey and Gly726 is colored by atom type. The topology of the protein backbone is schematically depicted with helices in light blue (SAM2 domain) and dark blue (SAM3 domain). (C) In the Gly726Val variant, a severe steric overlap (yellow circle) between the sidechains of Val726 and Asn803 is observed, which will disrupt the domain interface thereby altering the topology of the SAM domain region.

Furthermore, in a fetus, a homozygous missense variant, c.2177G>T (p.Gly726Val) was identified. The missense variant lies in the second SAM domain (Figure 2A) and affects a highly conserved amino acid considering nine species up to the opossum. Multiple *in silico* tools consistently predicted a damaging effect of the variant (Table S3). Structural analysis showed that Gly726 is located in a tight turn of the second SAM domain of *PPFIPB1* (Figure 2B). At this position, a valine can only be accommodated in a strained backbone conformation resulting in domain destabilization. In addition, the longer valine sidechain causes steric problems with Asn803 located in the third SAM domain (Figure 1B), which are predicted to disrupt the domain interface.

All of the identified variants are very rare in the general population, represented by gnomAD.^15^ The variants detected in the families 1, 2, 3, 4, 6, 8, 9, 10, 11, and 12 and in the fetus are absent from gnomAD. Five alleles are reported for the variant identified in family 5 (MAF of 0.0000199) and seven alleles are reported for the variant identified in family 7 (MAF of 0.00002828), all in heterozygous state in each case. The parents were confirmed as heterozygous carriers in the families 1, 2, 4, 5, 6, 7, 8, 10, 11, 12, and 13 by Sanger sequencing and/or trio ES/GS.

### Modeling loss of *PPFIBP1* in *C. elegans*

Worm models are useful for modeling the underlying mechanistic causes of genetic disorders. Automated quantitative phenotyping of the disease model mutant *hlb-1(syb4896)* was used to identify differences compared to the wild-type strain N2 across a range of behavioral dimensions.^38^

**Figure 3:**
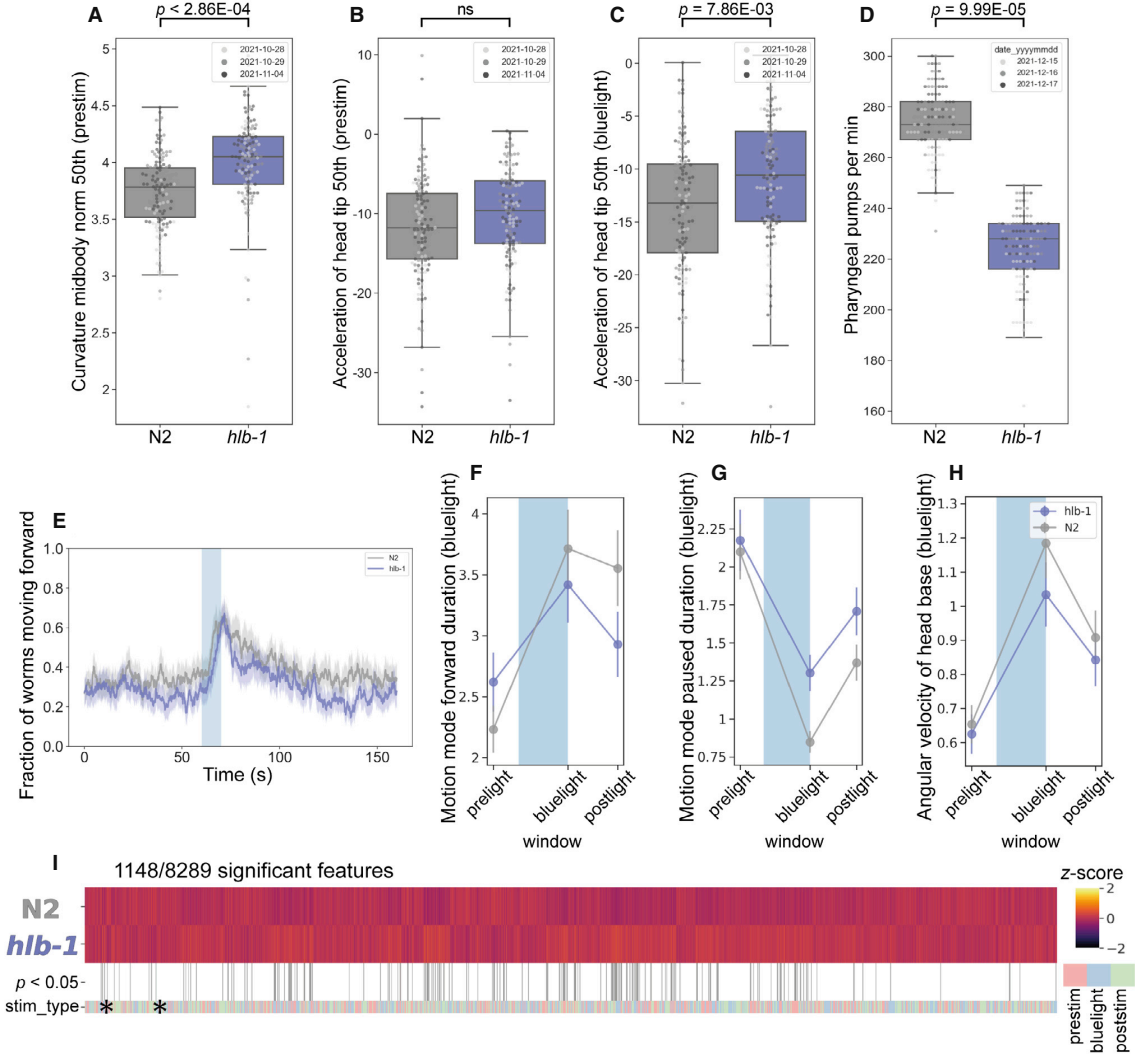
Behavioral phenotype of *Caenorhabditis elegans PPFIBP1* ortholog, *hlb-1(syb4896)* (A–C) Example behavioral and postural features altered in the loss-of-function *hlb-1(syb4896)* [*C. elegans* ortholog of *PPFIBP1*] mutant strain under baseline (pre-stimulus) imaging conditions. Individual points marked on the box plots are average values from multiple worms in a single well. The different point colors indicate data from independent experimental days. The selected features were compared to the N2 reference strain with block permutation t-tests, and p values are shown above the respective plots. (D) Pharyngeal pumps per minute of *hlb-1(syb4896)* and N2 reference strain. (E) Overall fraction of worms moving forward 60 s prior to and 80 s following stimulation with a 10 s blue light pulse (blue shading). Colored lines represent averages of the detected fraction of paused worms across all biological replicates and shaded areas represent the 95% confidence intervals. (F–H) Average changes in the total fraction of worms moving forward or paused prior to, during, and following stimulation with blue light (F and G), average change in an example postural feature in response to blue light (H). Feature values were calculated as averages of 10 s window summaries centered around 5 s before, 10 s after, and 20 s after the beginning of a 10 s blue light pulse (blue shading). (I) Heatmap of the entire set of 8,289 behavioral features extracted by Tierpsy for *hlb-1(syb4896)* and N2. The stim_type barcode denotes when during image acquisition the feature was extracted: pre-stimulation (pink), blue light stimulation (blue), and post-stimulation (green). Asterisks indicate the selected features present in the box plots above (A–C) and the color map (right) represents the normalized *Z* score of the features.

Loss of *hlb-1* did not result in developmental delay or a growth defect in *C. elegans*, however the *hlb-1(syb4896)* mutant showed a significant increase in body curvature (Figure 3A). In existing *C. elegans* models of epilepsy “head bobbing” is a phenotype associated with convulsions and the onset of seizures.^45^ We saw no statistically significant difference in the head movement of *hlb-1(syb4896)* compared to N2 during baseline (pre-stimulus) tracking (Figure 3B). However, upon stimulation with pulses of blue light, a significant increase in the acceleration of the head tip (indicative of increased head movement) was observed for mutant strains (Figure 3C), highlighting some overlap in the behavioral phenotype of *hlb-1(syb4896)* and other pre-existing worm models of epilepsy. Thus, this finding indirectly suggests some elements of a mild epileptic phenotype may be present in *hlb-1(syb4896)*.

There is little difference in the baseline locomotion of *hlb-1(syb4896)* and N2 (Figure 3E). However, *hlb-1(syb4896)* displays a short-lived photophobic escape response when pulsed with blue light, as demonstrated by the LoF mutant returning to a paused state faster upon the cessation of the aversive stimulus (Figures 3F–3G). We also note that there is an attenuated change in posture of *hlb-1(syb4896)* during blue light tracking (Figure 3H).

A previous study into the function of *hlb-1* in *C. elegans* identified a defect in pharyngeal pumping rate,^10^ which we also confirm for *hlb-1(syb4896)* (Figure 3D), and enlarged pre- and post-synaptic sites. Given the role of aberrant synaptic transmission events in the onset of epileptic seizures and the hypothesis that liprin-β1 acts as a core scaffold to mediate protein assembly in the presynaptic zone,^3^ we investigated whether our quantitative phenotyping approach could detect a defect in the synaptic transmission apparatus of *hlb-1(syb4896)*.

**Figure 4:**
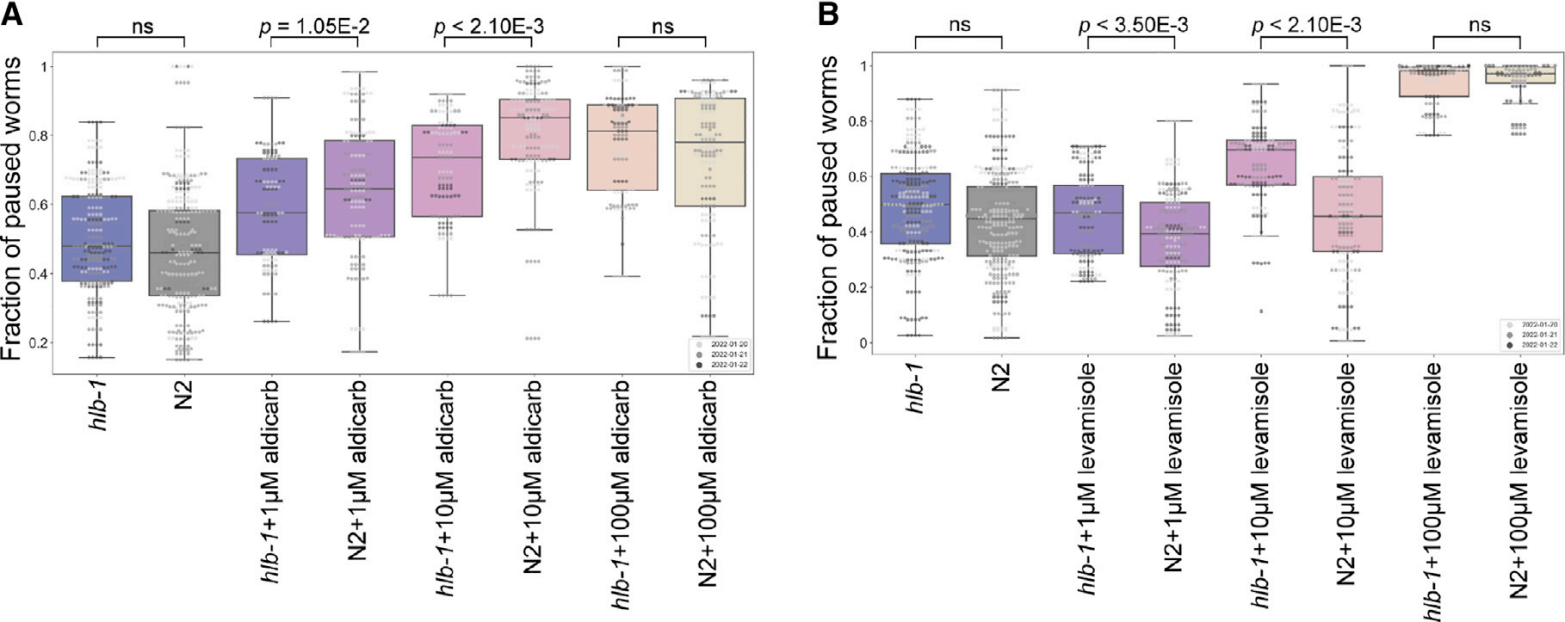
Fraction of paused worms in response to treatment with aldicarb or levamisole (A and B) Overall fraction of paused worms after (A) 1 h exposure to aldicarb and (B) 4 h exposure to levamisole at the concentrations denoted under the boxplots. N2 (grey) and *hlb-1* (blue) are solvent only controls (DMSO and ddH2O for aldicarb and levamisole, respectively). Individual points marked on the box plots are averaged values from multiple worms in a single well. The different point colors indicate data from independent experimental days. The fraction of paused *hlb-1(syb4896)* worms was compared to the fraction of paused N2 worms at each concentration with block permutation t-tests, with p > 0.05 considered not significant (ns), n = 30 wells for each compound and concentration tested.

Aldicarb is an acetylcholinesterase inhibitor that induces paralysis of the body-wall muscles in *C. elegans* as a result of an accumulation of acetylcholine (ACh) and the subsequent overstimulation of acetylcholine receptors. Increased resistance to aldicarb occurs if mutations give rise to defects in presynaptic function, as ACh accumulates in the neuromuscular junction at a slower rate.^46^ Indeed, *hlb-1(syb4896)* showed a significant dose-dependent decrease in the fraction of paused worms that were exposed to 1–10 μM aldicarb for 1 h compared to N2 (Figure 4A), demonstrating increased aldicarb resistance. Levamisole is a paralysis-inducing ACh receptor agonist. Resistance to levamisole has been shown to persist in worms if mutations affect the postsynaptic site, whereas sensitivity to levamisole persists if mutations only affect the presynaptic site.^47^ In contrast to previous *hlb-1* studies,^10^ we do not observe any resistance to levamisole in *hlb-1(syb4896)* worms. If anything, there is an increased sensitivity observed at 10 μM levamisole for 4 h (Figure 4B).

These findings provide evidence that a defect arises in the presynaptic, but not postsynaptic, apparatus of *C. elegans* as a result of *hlb-1* LoF. Coupled with existing evidence that liprins are involved in the assembly of presynaptic active zones across species,^3^^,^^7^ this points towards a conserved biological role of *hlb-1* and its orthologs in regulating the formation of NMJs and supports presynaptic defects as a cause of the pathologies arising from mutations in *PPFIBP1*.

## Discussion

Here we describe 16 individuals from 12 unrelated families with a core phenotype of moderate to profound developmental delay, progressive microcephaly, epilepsy, and periventricular calcifications. In all 16 individuals, ES and GS revealed rare homozygous LoF variants in *PPFIBP1*. In addition, we describe a fetus with severe growth restriction, microcephaly, and intracranial calcifications with a homozygous missense variant that is *in silico* and structurally predicted to be disrupting.

Consistent with the proposed autosomal recessive inheritance, LoF variants in *PPFIBP1* in the general population are not common with an observed/expected ratio (o/e) of 0.57 (90% confidence interval = 0.43–0.75). In addition, there were no homozygous LoF variants observed in gnomAD. Because all described variants are ultra-rare (MAF < 0.01%), it is highly unlikely to assemble a cohort with this level of phenotypical overlap and homozygous LoF variants in *PPFIBP1* by coincidence, which further strengthens disease causality.

The 15 individuals harboring homozygous frameshift or nonsense variants exhibit a consistent phenotype in terms of the severity of the developmental delay, epilepsy, and frequently found neuroimaging features. Only individual 7 presented a milder disease course compared to the other individuals with truncating variants, as she had secondary microcephaly, was able to stand and walk, albeit showing impaired motor development, and showed less prominent neuroimaging features. The nonsense variant c.403C>T (p.Arg135^∗^) found in individual 7 lies in the 6^th^ exon thus being the most upstream variant in this cohort. It is predicted to cause NMD considering the transcript GenBank: NM_003622.4, which shows the highest overall expression and thereby most likely has the highest biological relevance. Nevertheless, it cannot be ruled out that a shorter transcript like GenBank: NM_001198915.2 with its start codon lying 57 base pairs downstream of this variant could compensate for the loss of the main transcript to some extent. GenBank: NM_001198915.2 has the second highest mean expression across all tissues and particularly shows expression levels that are comparable to those of GenBank: NM_003622.4 in some areas of the brain.^16^

Individual 1 with the homozygous splice-site variant has a milder phenotype compared to the other individuals with nonsense or frameshift variants, although he shares the core clinical signs. This could be due to an incomplete splice defect, either leading to the expression of a fraction of normal protein or to an altered protein not completely impaired in function or stability. Canonical splice site variants as observed in individual 1 can have a variety of effects on pre-mRNA splicing such as exon skipping, which is the most common mechanism in variants disrupting consensus 5′-donor splice sites^43^ and would result in a frameshift in this case. However, a loss of the splice site could also result in intron retention with a premature termination-codon or enable the activation of a cryptic splice site with subsequent inclusion of an intron fragment or the removal of an exon fragment either inframe or out of frame. Both of the latter possibilities can lead to a variety of aberrant transcripts.

The pathogenicity of the missense variant identified in the fetus is not as clear as that of the LoF variants. However, the striking similarity of the intracranial calcifications, the growth restriction, and the severe microcephaly represent a significant phenotypic overlap with the rest of the cohort, suggesting this variant to be causative. Potential pathogenicity of the variant is further supported by its absence from the general population, by multiple *in silico* predictions and its expected effects on the SAM domains from structural analysis. SAM domains are a family of protein interaction modules present in a wide variety of proteins.^48^ The Gly726Val exchange is located in the second SAM domain of PPFIBP1 destabilizing both the second SAM domain and the interaction between the second and third SAM domain. Therefore, this variant is expected to severely disturb the topology of the SAM domain region and its function in protein-protein interactions. Given the hypothesis that liprin-β1 acts as a core scaffold to mediate protein assembly in the presynaptic zone,^3^ the ability to precisely interact with other proteins would appear to be critical for protein function.

ICCs located in the periventricular area but also affecting the basal ganglia and the internal capsule appear to be a highly characteristic sign in this cohort. Pathologic ICCs have heterogeneous etiologies such as neoplastic, infectious, vascular, metabolic, and genetic conditions.^49^ Congenital infections with pathogens of the TORCH-spectrum, and congenital cytomegalovirus (CMV) infections in particular, account for a significant amount of congenital and pediatric ICCs that are associated with brain malformations and impaired neurodevelopment.^50^ However, genetic disorders such as interferonopathies represent important differential diagnoses for congenital ICCs and some conditions significantly overlap with the symptomatic spectrum of congenital TORCH-infections.^51^, ^52^, ^53^ It is assumed that the genetic etiologies of unsolved ICCs have not been fully discovered yet.^50^^,^^54^ Individual 4 was admitted to the neonatal intensive care unit for 1 month after birth, as his clinical presentation was indicative of a congenital CMV infection (see supplemental notes for further details). However, an active CMV infection could not be confirmed in standard laboratory diagnostics. In both affected siblings of family 5 and in individual 6-2, a screening for infections of the TORCH spectrum was performed with negative results and also the fetus was tested negative for CMV.

To date, no alterations in any of the human liprin genes have been associated with human disease. The biological function of liprin-β1 and its molecular mechanisms are still largely unstudied. However, recent studies point towards a role in neurodevelopment that echo the findings of a neurodevelopmental disorder in the cohort described here. Liprin- β1 has been identified as a binding partner of liprin-α proteins. The role of liprin-α proteins or their orthologues in synapse formation and synaptic transmission has been demonstrated in previous animal model studies.^7^^,^^55^, ^56^, ^57^ Liprin-α proteins function as major scaffold proteins at the presynaptic active zone and at the postsynaptic density and also play a role in intracellular transport, cell motility, and protein assembly.^3^^,^^5^^,^^6^^,^^58^, ^59^, ^60^ Wei et al. found that liprin-α2 forms a ternary complex simultaneously binding liprin-β1 and CASK, another presynaptic scaffold protein, supporting the hypothesis that liprin-β1 could act as a core scaffold and mediate large protein assemblies in the presynaptic active zone.^3^ Interestingly, pathogenic variants in *CASK* (MIM: 300172) are associated with X-linked neurodevelopmental disorders.^61^ Pathogenic variants in *CASK* cause X-linked neurodevelopmental disorders with varying phenotypes depending on variant type and inheritance. In particular, heterozygous and hemizygous LoF variants in *CASK* lead to microcephaly with pontine and cerebellar hypoplasia (MICPCH [MIM: 300749]). The phenotypic spectrum comprises moderate to profound ID, progressive microcephaly, impaired hearing, ophthalmologic anomalies, muscular hypo- or hypertonia and spasticity, as well as seizures and partly epileptic encephalopathy in males.^61^ Because the phenotype is overlapping with the clinical signs found in this cohort, it seems possible that *PPFIBP1* and *CASK* are involved in similar biological functions such as protein assembly in the presynaptic active zone. Supporting the potential role of liprin-β1 in synapse formation and neurodevelopment, we have shown here that a *C. elegans PPFIBP1/hlb-1* knockout model shows defects in spontaneous and light-induced behavior. The observed sensitivity of the worm model to the acetylcholinesterase inhibitor aldicarb supports a presynaptic defect as at least a partial cause of the observed behavioral phenotypes. This is broadly consistent with previous work showing that null-allele mutants of the *drosophila* orthologs liprin-β and liprin-α independently cause abnormal axon outgrowth, target layer recognition, and synapse formation of R7 photoreceptors as well as reduced larval NMJ size in *Drosophila melanogaster*. Interestingly, distinct effects on axon outgrowth between single liprin-β and liprin-α mutants and an additive effect in double mutants were observed, indicating independent functions of both proteins.^7^

In summary, we establish bi-allelic loss-of-function variants in *PPFIBP1* as a cause for an autosomal recessive severe neurodevelopmental disorder with early-onset epilepsy, microcephaly, and periventricular calcifications.

### Data and code availability

All identified variants in *PPFIBP1* have been uploaded to ClinVar https://www.ncbi.nlm.nih.gov/clinvar/submitters/506086/ with the following accession numbers: c.1146+1G>A (p.?) in individual 1 (ClinVar: VCV001679175); c.2654del (p.Tyr885Leufs^∗^4) in individuals 2 and 12 (ClinVar: VCV001679176); c.1368_1369del (p.Glu456Aspfs^∗^3) in individuals 3-1, 3-2, 3-3, and 4 (ClinVar: VCV001679177); c.2413C>T (p.Arg805^∗^) in individuals 5-1 and 5-2 (ClinVar: VCV001679178); c.1468C>T (p.Gln490^∗^) in individuals 6-1, 6-2, and 11 (ClinVar: VCV001679179); c.403C>T (p.Arg135^∗^) in individual 7 (ClinVar: VCV001679180); c.1417_1427del (p.Ala473Lysfs^∗^20) in individual 8 (ClinVar: VCV001679181); c.1300C>T (p.Gln434^∗^) in individual 9 (ClinVar: VCV001679182); c.2629C>T (p.Arg877^∗^) in individual 10 (ClinVar: VCV001679183); c.2177G>T (p.Gly726Val) in the fetus (ClinVar: VCV001679120); c.2158+2T>C (p.?) in individual 14 (see supplemental information). The code used for tracking and extracting *C. elegans* behavioral features is available at https://github.com/Tierpsy and code for performing statistical analysis and generating figures is available at https://github.com/Tom-OBrien/Phenotyping-hlb1-disease-model-mutant. The associated *C. elegans* datasets are available at https://doi.org/10.5281/zenodo.6338403. The Morbid Genes Panel is available here https://morbidgenes.org/ and here https://zenodo.org/record/6136995#.YiYvI-jMKUk.

## Web resources

BLAST, https://blast.ncbi.nlm.nih.gov/Blast.cgi
Ensembl, https://www.ensembl.org/index.html
GeneMatcher, https://genematcher.org/
GenBank, https://www.ncbi.nlm.nih.gov/genbank/
gnomAD, https://gnomad.broadinstitute.org/
GTEx, https://gtexportal.org/home/
MorbidGenes, https://morbidgenes.org/
OMIM, https://www.omim.org/
PDB, https://www.rcsb.org/
SpliceAI, https://spliceailookup.broadinstitute.org/
SwissModel, https://swissmodel.expasy.org/

## Supplemental information

Document S1. Supplemental notes, Figures S1 and S2, Tables S1–S3, and supplemental methods

Table S4. Full clinical details of all affected individuals with causative variants in *PPFIBP1*

Document S2. Article plus supplemental information

## Footnotes

Supplemental information can be found online at https://doi.org/10.1016/j.ajhg.2022.06.008.
