# Supplementary material for: Bi-allelic loss-of-function variants in PPFIBP1 cause a neurodevelopmental disorder with microcephaly, epilepsy, and periventricular calcifications
Source: Am J Hum Genet. 2022 Jul 12;109(8):1421–35. doi: 10.1016/j.ajhg.2022.06.008 (PMC9388382; doi:10.1016/j.ajhg.2022.06.008)
Supplement: Document S1. Supplemental notes, Figures S1 and S2, Tables S1–S3, and supplemental methods [file mmc1.pdf]

The American Journal of Human Genetics, Volume *109*

Supplemental information

Bi-allelic loss-of-function variants in *PPFIBP1* cause a neurodevelopmental disorder with microcephaly, epilepsy, and periventricular calcifications

Erik Rosenhahn, Thomas J. O'Brien, Maha S. Zaki, Ina Sorge, Dagmar Wieczorek, Kevin Rostasy, Antonio Vitobello, Sophie Nambot, Fowzan S. Alkuraya, Mais O. Hashem, Amal Alhashem, Brahim Tabarki, Abdullah S. Alamri, Ayat H. Al Safar, Dalal K. Bubshait, Nada F. Alahmady, Joseph G. Gleeson, Mohamed S. Abdel-Hamid, Nicole Lesko, Sofia Ygberg, Sandrina P. Correia, Anna Wredenberg, Shahryar Alavi, Seyed M. Seyedhassani, Mahya Ebrahimi Nasab, Haytham Hussien, Tarek E.I. Omar, Ines Harzallah, Renaud Touraine, Homa Tajsharghi, Heba Morsy, Henry Houlden, Mohammad Shahrooei, Maryam Ghavideldarestani, Ghada M.H. Abdel-Salam, Annalaura Torella, Mariateresa Zanobio, Gaetano Terrone, Nicola Brunetti-Pierri, Abdolmajid Omrani, Julia Hentschel, Johannes R. Lemke, Heinrich Sticht, Rami Abou Jamra, Andre E.X. Brown, Reza Maroofian, and Konrad Platzer

Contents

Supplemental note: Case Reports ... 3
Individual 1 ... 3
Individual 2 ... 4
Individual 3-1, 3-2 and 3-3 ... 5
Individual 4 ... 8
Individual 5-1 and 5-2 ... 10
Individual 6-1 and 6-2 ... 13
Individual 7 ... 15
Individual 8 ... 16
Individual 9 ... 17
Individual 10 ... 18
Individual 11 ... 19
Individual 12 ... 20
Fetus (family 13) ... 22
Additional Case: Individual 14 ... 22
Figure S1. ... 24
Figure S2. ... 25
Table S1 ... 27
Table S2 ... 28
Table S3 ... 29
Supplemental Methods ... 30
Use of GTEx Expression Data ... 30
Supplemental sequencing methods ... 30
Individual 1 ... 30
Individual 2 ... 31
Individuals 3-1, 3-2 and 3-3 ... 31
Individual 4 ... 31
Individuals 5-1, 5-2, 8 and 10 ... 32
Individuals 6-1, 6-2 and 11 ... 32
Individual 7 ... 33
Individual 9 ... 33
Individual 12 ... 33
Fetus (Family 13) ... 34
RNA analysis ... 34
Family 1 (Figure S1) ... 34
Family 7 (Figure S2) ... 35

Supplemental References 36

Supplemental note: Case Reports

Individual 1

The male individual was born to healthy consanguineous parents (first cousins) of Turkish origin. The family history was uneventful. Pregnancy was uneventful besides growth retardation and oligohydramnios. He was born at 40+3 gestational weeks with a weight of 2650 g (-1.53 SD), a length of 49 cm (-1.44 SD) and an occipitofrontal circumference (OFC) of 33 cm (-1.15 SD). After birth, neonatal icterus and a ptosis of the left eye were noticed, he slept a lot.

Mild to moderate developmental delay was diagnosed in kindergarten. He visited a school for intellectually handicapped children. Focal impaired awareness seizures started at the age of four years with an initial frequency of two seizures per month and they required therapy with Lamotrigine and Oxcarbazepine. At the time of last examination, individual 1 was 19 years old. He presented with microcephaly (52 cm [<-2.5 SD]) and normal height (166 cm [-1.44 SD]) and weight (79 kg [0.78 SD]). At the time had focal seizures every 1-2 months and still received Lamotrigine and Oxcarbazepine. He was able to write his name, to recognize numbers and read single words. He had a large nose, short philtrum and a small chin.

Brain MRI at the age of 19 years did not reveal any anomalies.

Chromosomal analysis, SNP array and panel sequencing was normal. Trio exome sequencing revealed a homozygous splice variant in *PPFIBP1* (NM_003622.4): c.1146+1G>A. Both parents were confirmed heterozygous for this variant by Sanger sequencing and were phenotypically normal.

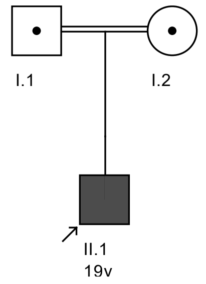

Individual 2

The female individual was born at term to unaffected and fourth degree consanguineous parents of Syrian origin. Two siblings of the individual passed away in childhood. One of them presented epilepsy and microcephaly and the second died due to high grade glioma. Neither of them was genetically tested. Pregnancy and neonatal period were uneventful.

At the age of six years, she presented with short stature (95 cm at 5 years and 4 months [-3.4 SD]), severe microcephaly (42 cm [<< -3 SD]) and low weight (12 kg [-4.14 SD]). She had profound intellectual disability (ID), absent speech, spastic tetraplegia and severely delayed motor development being unable to sit or walk independently. Seizures started at two months of age and she was lastly affected by focal as well as bilateral tonic-clonic seizures. EEG findings included a temporal paroxysmal focus with wave spikes during sleep. She received antiepileptic treatment with Levetiracetam and Clonazepam which controlled the seizures. Furthermore, she was affected by laryngo-pharyngo-tracheomalacia requiring gastrostomy at the age of five years, left hypoacusis, bilateral papillary pallor and a nystagmus on the left. Dysmorphic features included long philtrum, wide nasal bridge, low columella, synophris and low set ears. She died at the age of six years of cardiac and respiratory arrest, likely linked to intractable seizures.

Brain MRI was performed at the age of five years and revealed ventriculomegaly, leukoencephalopathy, paucity of the white matter and thalamus, microcalcification in basal ganglia, hypoplasia of the splenium and the knee of the corpus callosum and a Blake's pouch cyst. Cranial CT showed bilateral calcifications of the thalamus and periventricular along the wall of the lateral ventricles.

Trio exome sequencing revealed a homozygous frameshift variant in *PPFIBP1* (NM_003622.4): c.2654del, p.(Tyr885Leufs*4). The parents were heterozygous for this variant. In two daughters of a paternal cousin who presented ID, hypotonia and hyporeflexia with respiratory insufficiency and feeding difficulties, a homozygous mutation in *TBCK* was found. The mutation was not present in the individual described here.

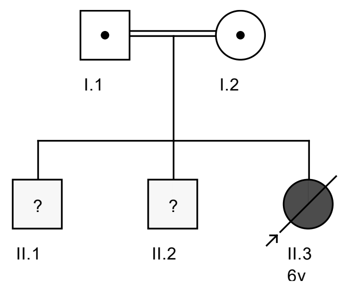

Individual 3-1, 3-2 and 3-3

Individual 3-1:

The male individual was born to healthy consanguineous parents. In the family there are two other similarly affected siblings (individuals 3-2 and 3-3) and a fourth sibling died shortly after birth at the age of 6 days and was not molecularly tested.

After an uneventful pregnancy, individual 3-1 was born at term (37+6 gestational weeks) with a weight of 2200 g (-2.67 SD), a length of 44 cm (-3.11 SD) and an OFC of 28.5 cm (-4.69 SD). After birth, two episodes of apnea were reported.

At the age of 11 years, he presented decreased body weight of 15 kg (<<-3 SD) and severe microcephaly with an OFC of 41 cm (<<-3 SD). He was affected by profound global developmental delay (GDD)/ ID, absent speech, spastic tetraplegia and severely delayed motor development being unable to sit or walk independently. He achieved social smiling at the age of 12 months. Seizures started at the age of seven months as epileptic spasms that evolved to focal, focal to bilateral tonic-clonic, and tonic seizures that occurred daily. Seizures were refractory to multiple antiepileptic drugs (Phenobarbital, Vigabatrin, Clonazepam). EEG findings included hypsarrhythmia and multifocal epileptiform discharges. Furthermore, he was noted to have impaired hearing, congenital heart defects (atrial septal defect and patent ductus arteriosus), a single kidney and café au lait spots.

CT and MRI imaging performed in the individual revealed bilateral intracranial calcifications in the periventricular region and in the centrum semiovale, periventricular leukomalacia and metopic synostosis.

Singleton exome sequencing revealed a homozygous frameshift variant in *PPFIBP1* (NM_003622.4): c.1368_1369del, p.(Glu456Aspfs*3). Exome sequencing also identified a homozygous missense variant c.5814C>A, p.(Asp1938Glu) in *CENPF* (GenBank: NM_016343.3; MIM: 600236) which is associated with Stromme syndrome (MIM: 243605) caused by bi-allelic truncating variants. Since up to date, no (likely) pathogenic missense variants were described to cause this condition and as his very similarly affected brother (Individual 3-2) was found heterozygous for this variant, it was not considered to be causative. The individual was tested positive for increased chromosomal breakage.

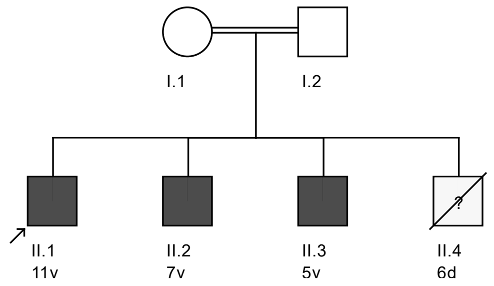

Individual 3-2:

The male individual was born to healthy consanguineous parents. In the family there are two other similarly affected siblings (individuals 3-1 and 3-3) and a fourth sibling died shortly after birth at the age of 6 days and was not molecularly tested.

After an uneventful pregnancy, individual 3-2 was born at term (37 gestational weeks) with a weight of 2200 g (-2.67 SD), a length of 46 cm (-2.05 SD) and an OFC of 31.5 cm (-2.33 SD). The neonatal period was uneventful

At the age of seven years, he presented decreased body weight of 14 kg (-3.84 SD) and severe microcephaly with an OFC of 44 cm (<<-3 SD). He was affected by severe GDD/ID, absent speech, spastic tetraplegia and severely delayed motor development being unable to sit or walk independently. He achieved social smiling at the age of 10 months. Seizures started at the age of two to three months as epileptic spasms. Epilepsy evolved to Lennox-Gastaut syndrome in the course. Seizures were very frequent and refractory to multiple antiepileptic drugs (Levetiracetam, Topiramate, Vigabatrin, Clonazepam). EEG showed hypsarrhythmia and in the course Lennox-Gastaut syndrome pattern.

Furthermore, he was noted to have impaired hearing, congenital heart defects (atrial septal defect, patent ductus arteriosus, small ventricular septal defect) and undescended testes.

MRI performed at the age of 3 days and 2 years showed some punctual hyperintensities in the basal ganglia progressing to moderate hyperintensities in the periventricular white matter and mild ventriculomegaly. CT scan revealed bilateral intracranial calcifications in the periventricular region and in the centrum semiovale,

Singleton exome sequencing revealed a homozygous frameshift variant in *PPFIBP1* (NM_003622.4): c.1368_1369del, p.(Glu456Aspfs*3).

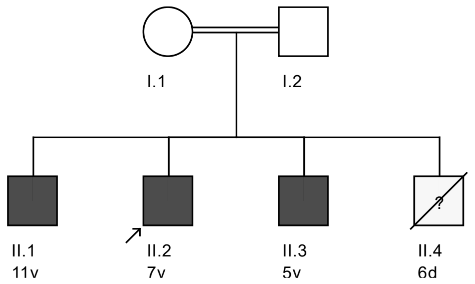

Individual 3-3:

The male individual was born to healthy consanguineous parents. In the family there are two other similarly affected siblings (individuals 3-1 and 3-2) and a fourth sibling died shortly after birth at the age of 6 days and was not molecularly tested.

Individual 3-3 was born late pre-term (36+3 gestational weeks) with a weight of 1800 g (-2.42 SD), a length of 43 cm (-1.92 SD) and an OFC of 29.5cm (-2.33 SD). During pregnancy intrauterine growth retardation was noted.

At the age of five years he presented decreased body weight of 8.5 kg (-5.28 SD) and severe microcephaly with an OFC of 39.5 cm (<< -3 SD). He was affected by severe GDD/ID, absent speech, spastic tetraplegia and severely delayed motor development being unable to sit or walk independently. He achieved social smile at the age of 12 months. Seizures started at the first day of life and evolved from left focal seizures to epileptic spasms and multifocal epilepsy. Seizures were refractory to multiple antiepileptic drugs (Levetiracetam, Phenobarbital, Vigabatrin). EEG showed right hemispheric mainly

temporal epileptiform discharges. Furthermore, he was noted to have impaired hearing, congenital heart defects (atrial septal defect, patent ductus arteriosus), undescended testes and a right ectopic pelvic kidney.

MRI from the third day of life showed ventriculomegaly with irregular walls of the lateral ventricles, T1-hyperintensity and T2-hypoinenstity in the periventricular white matter and in the basal ganglia, abnormal signal intensity of the white matter on the right side, and bilateral temporal and left occipital thickening of the cortex (pachygyria). CT scan revealed bilateral calcifications in the periventricular white matter, the ventricles wall and in basal ganglia.

Singleton exome sequencing revealed a homozygous frameshift variant in *PPFIBP1* (NM_003622.4): c.1368_1369del, p.(Glu456Aspfs*3). Exome sequencing also identified a homozygous missense variant c.5814C>A, p.(Asp1938Glu) in *CENPF* (GenBank: NM_016343.3; MIM: 600236) which is associated with Stromme syndrome (MIM: 243605) caused by bi-allelic truncating variants. Since up to date, no (likely) pathogenic missense variants were described to cause this condition and as his very similarly affected brother (Individual 3-2) was found heterozygous for this variant, it was not considered to be causative.

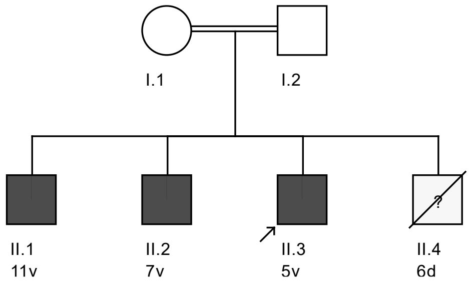

Individual 4

The male individual was born via normal vaginal delivery after 40 weeks of pregnancy as the first child of healthy consanguineous first-degree-cousin parents. The mother had a vaginal infection during pregnancy. Intrauterine growth restriction was evident in the prenatal ultrasound. At birth, his bodyweight was 2110 g (-2.92 SD), his length was 45 cm (-2.58 SD), his head circumference was 30 cm (-3.51 SD) and Apgar score was 7 and 9 at 1 and 5 minutes, respectively. He stayed in NICU for one

month with the impression of a congenital cytomegalovirus (CMV) infection based on the clinical presentation of microcephaly, intracranial calcifications, haemorrhagic retinitis, neonatal hypoglycaemia, neonatal seizure, hyperbilirubinemia and congenital heart disease with VSD of 5 mm, small PDA, small ASD and dilated left ventricle (LV) and left atrium (LA) with left to right shunt. CMV IgG serology was positive, but urine CMV PCR was negative. He received an intravenous course of Ganciclovir.

After discharge from NICU, he continued to have severe global developmental delay and recurrent admissions to the hospital. Chronic retinal detachment was found at 5 months of age. Also, he developed different types of seizures including focal onset seizures, tonic seizures and infantile spasms. His antiepileptic drugs included Phenobarbitone, Levetiracetam, Vigabatrin and Topiramate.

Currently, he can make cooing sounds and shows some interest in lights. On the most recent examination at 11 months of age, his head circumference was 39 cm (<< -3 SD), his weight was 8.5 kg (-0.93 SD) and his length was 67 cm (-2.94 SD). He had dysmorphic features including microcephaly, bitemporal hollowing, broad nasal bridge, long philtrum, micrognathia, short neck, low set ears, prominent antihelix ears, and bilateral undescended testes. He had right eye leukocoria, right eye exotropia with slow pupillary reaction on the same eye. His gag reflex was fair. He had spontaneous antigravity movements of his limbs, hypertonia with spasticity of his elbows, wrists, hips and knees, hyperreflexia with spreading and crossing and positive clonus.

Singleton exome sequencing revealed a homozygous frameshift variant in *PPFIBP1* (NM_003622.4): c.1368_1369del, p.(Glu456Aspfs*3). The parents were phenotypically normal and confirmed as heterozygous carriers of the variant.

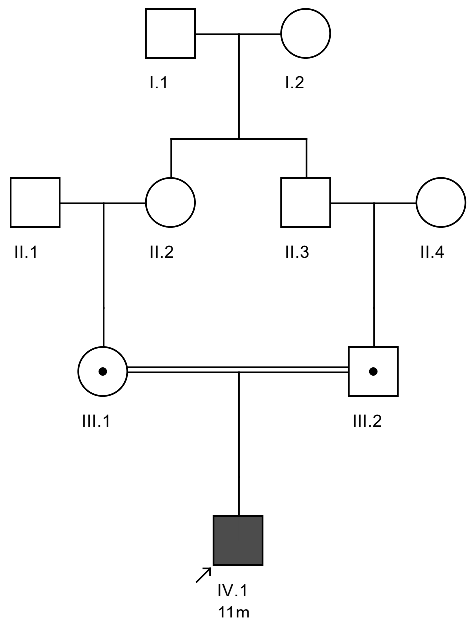

Individual 5-1 and 5-2

Individual 5-1:

The female individual was born to unaffected consanguineous (second degree) parents of Egyptian origin. She has a similarly affected sibling (see individual 5-2). Pregnancy was uneventful and she was born at term (39 gestational weeks) with a weight of 2500 g (-1.73 SD), length of 46 cm (-1.69 SD) and microcephaly with an OFC of 30 cm (-3.27 SD). After birth she presented with mild cyanosis and weak crying.

At the last assessment at the age of eight years she presented short stature (97 cm [-5 SD]), decreased weight (18 kg [-2.24 SD]) and severe progressive microcephaly (OFC = 42,5 cm [<< -3 SD]). She was affected by profound ID, absent speech, spastic tetraplegia and was unable to sit or walk. Seizures started at the age of two months, initially occurred daily and included focal seizures with head deviation and myoclonic epilepsy as clusters. By treatment with multiple antiepileptic drugs (VPA, CNZ, CBZ, LEV, LTG) seizures could be fairly controlled but seizures always recurred again in the course. EEG showed bilateral temporal paroxysmal discharges. Further symptoms included acquired scoliosis from spasticity

and a deglutition disorder with feeding difficulties, failure to thrive and infrequent vomiting. As a dysmorphic feature, bitemporal hollowing was noted. She died at the age of eight years.

Cranial CT showed periventricular calcifications with a linear pattern surrounding the lateral ventricles. Brain MRI was not performed.

TORCH screening, extended Metabolic screening and karyotyping were normal. Quadruple exome sequencing revealed a homozygous nonsense variant in *PPFIBP1* (NM_003622.4): c.2413C>T, p.(Arg805*) that was also detected in the similarly affected sibling in homozygous state. Her parents were phenotypically normal and found to be heterozygous carriers of the variant.

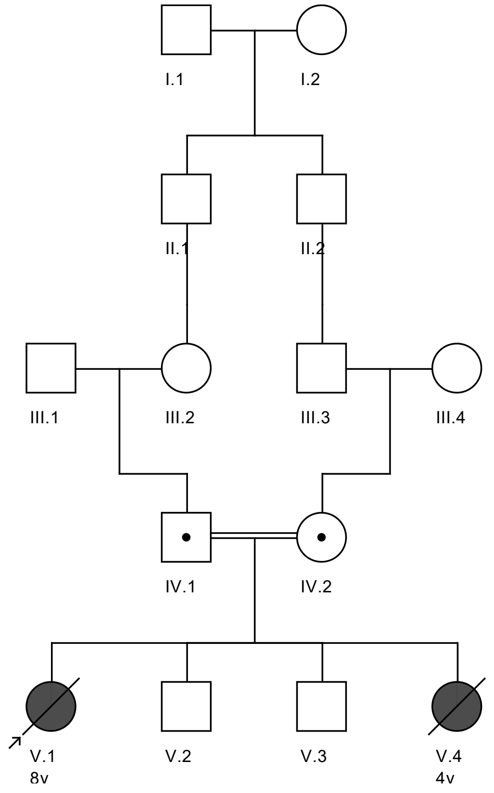

Individual 5-2:

The female individual was born to unaffected consanguineous (second degree) parents of Egyptian origin. Apart from a peptic ulcer in the mother, pregnancy was uneventful and she was born at term (38 gestational weeks) with a weight of 2700 g (-1.23 SD), a length of 47 cm (-1.15 SD) and microcephaly with an OFC of 30.5 cm (-2.85 SD).

At the last assessment at the age of two years she presented short stature (77 cm [-2.71 SD]), decreased body weight (9.5 kg [-1.57 SD]) and severe progressive microcephaly (OFC = 39.5 cm [<< -3 SD]). She was affected by profound ID, absent speech, hypertonia of the limbs, dystonia and was unable to sit or walk. Seizures started at the age of one month, initially as daily focal seizures with versive head movement that, after one month, evolved to myoclonic, focal and sometimes tonic seizures partly associated with cyanosis. She was treated with multiple antiepileptic drugs (VPA, CBZ, LEV, LTG) but seizures were refractory. EEG showed active left centro-temporal epileptogenic discharges. She experienced feeding difficulties. As dysmorphic features bitemporal hollowing, an upturned nose, a long philtrum and low set ears were noted. She died at the age of four years.

Cranial CT showed periventricular calcifications with a linear pattern surrounding the lateral ventricles and an enlarged ventricular system. Brain MRI was not performed.

TORCH screening was normal. Quadruple exome sequencing revealed a homozygous nonsense variant in *PPFIBP1* (NM_003622.4): c.2413C>T, p.(Arg805*) that was also detected in the similarly affected sibling in homozygous state. Her parents were phenotypically normal and found to be heterozygous carriers of the variant.

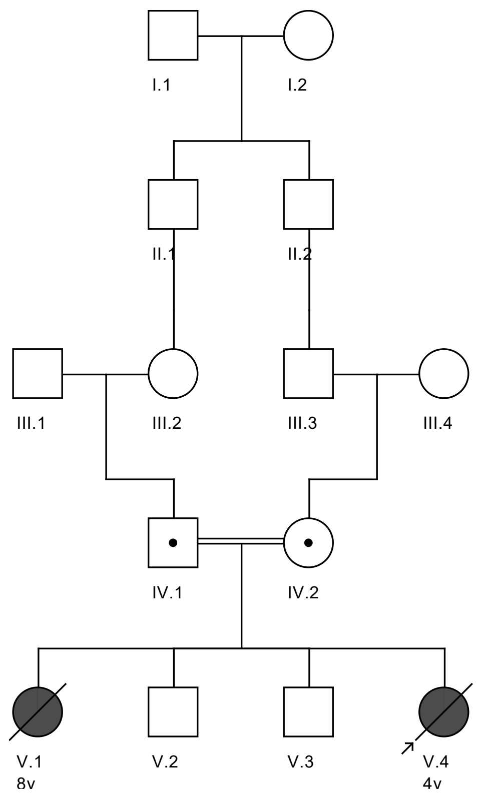

Individual 6-1 and 6-2

Individual 6-1:

The male individual was born to healthy first-cousin parents of Egyptian origin. He has a similarly affected younger brother (individual 6-2) and an unaffected brother. The pregnancy was uneventful and he was born at term (40 gestational weeks) with a weight of 2900 g (-0.96 SD), a length of 49 cm (-0.47 SD) and with an OFC of 32 cm (-1.94 SD).

At the last assessment at the age of six years he presented short stature (103 cm [-2.63 SD]), decreased body weight (15.3 kg [-2.3 SD]) and severe progressive microcephaly (OFC = 42.2 cm [<< -3 SD]). He was affected by profound ID, absent speech, hypertonia of the limbs and was unable to walk but could sit independently at the age of 6 years. Seizures started at the age of 4 months as myoclonic, followed by generalized tonic-clonic seizures and attacks of cyanosis, especially occurring together with fever. He was treated with multiple antiepileptic drugs (VPA, LEV, CZP) and the seizures could be fairly controlled and reduced to about one per month. EEG showed multifocal epileptiform discharges with abnormal background activity. Ophthalmological examination revealed optic atrophy and he was only

able to follow light. He experienced feeding difficulties was unable to masticate. As dysmorphic features a long face, prominent supraorbital ridges, sparse eyebrows, a prominent and upturned nose, a long philtrum, a v shaped upper lip, low set large ears with prominent antihelix and a retruded mandible were noted.

Brain MRI at the age of 4 years showed mild ventriculomegaly, cortical atrophy, a deep Sylvian fissure, periventricular and deep white matter demyelination mainly around frontal and occipital horn, a thin corpus callosum, and cerebellar vermian hypoplasia.

Metabolic screening and karyotyping were normal. Singleton exome sequencing revealed a homozygous nonsense variant in *PPFIBP1* (NM_003622.4): c.1468C>T, p.(Gln490*) that was also detected in the similarly affected sibling in homozygous state. His parents were phenotypically normal and heterozygous for this variant by Sanger sequencing.

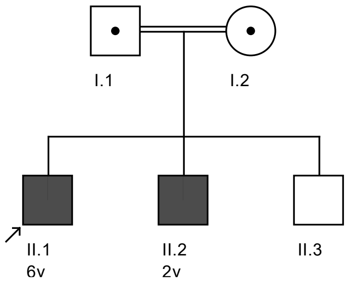

Individual 6-2:

The male individual was born to healthy first-cousin parents of Egyptian origin. He has a similarly affected older brother and an unaffected younger brother. The pregnancy was uneventful and he was born at term (39 GW) with a weight of 2800 g (-1.18 SD), a length of 47 cm (-1.52 SD) and microcephaly with an OFC of 31.8 cm (-2.10 SD). After birth he was admitted to hospital with jaundice for phototherapy.

At the last assessment at the age of two years he presented with short stature (79 cm [-2.66 SD]), decreased body weight (9.1 kg [-2.54 SD]) and severe progressive microcephaly (OFC = 40 cm [<< -3 SD]). He was affected by profound GDD with absent speech and no head support. He showed high spasticity, rigidity and dystonic movements. Seizures started at the age of two months and included

myoclonic, generalized tonic-clonic and tonic seizures with upward gaze and he showed excessive smacking movements. He was treated with multiple antiepileptic drugs (VPA, LEV, CZP) but seizures were refractory. EEG showed active multifocal spikes more in right temporoparietal area. Ophthalmological examination revealed optic atrophy and he was not able to follow light. Further symptoms included feeding difficulties and a persistent ductus arteriosus which was operated at one year of age. As dysmorphic features a long face, a high forehead, prominent supraorbital ridge, sparse eyebrows, a prominent and upturned nose, a long philtrum, a V-shaped upper lip, low set large ears with a prominent antihelix, retruded mandible and chin dimple were noted.

Brain MRI at the age of 8 months showed an asymmetric dilatation of the lateral ventricles with more dilatation on the right side, cortical atrophy, deep Sylvian fissures, periventricular and deep white matter demyelination mainly around frontal and occipital horn, a thin corpus callosum and cerebellar vermian hypoplasia.

Karyotyping, metabolic screening and TORCH screening were normal. Singleton exome sequencing revealed a homozygous nonsense variant in *PPFIBP1* (NM_003622.4): c.1468C>T, p.(Gln490*) that was also detected in the similarly affected sibling in homozygous state. His parents were phenotypically normal and heterozygous for this variant by Sanger sequencing.

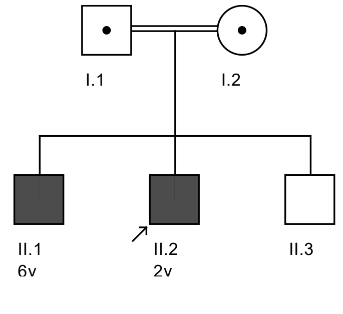

Individual 7

Individual 7 is the first common child of parents from the same area in northern Sweden. She was born full term, and already from the start she gave less contact and appeared hypotonic as compared to other children. Around 4 months of age she started having apneic episodes, and EEG confirmed epileptic activity. Her seizures progressed into infantile spasms at 7 months of age. She was treated with ACTH,

but the spasms recurred and Vigabatrin and Topiramate were added. An initial MRI performed at 4 months of age was normal. She continued to have multiple seizure types including myoclonias. At 1,5 years of age Valproic acid was added which controlled the seizures well. Concomitantly, a new MRI at 1,5 years of age showed a thin corpus callosum, patchy dysmyelination in periventricular regions, and possibly a reduction of the white matter. Analysis of amino acids, pipecolic acid, and very long chain fatty acids in plasma were all normal, as were organic acids, purine and pyrimidine metabolites and amino acids in urine. In her lumbar puncture she had increased levels of Glial Fibrillary Acidic Protein (GFAP) and Neurofilament light (NfL), indicating active destruction. Her head circumference was normal at birth, but at 2 years of age clearly microcephalic. Her development was now at 2 years of age delayed. She was not spastic, but she did have lively reflexes. She had a wide based gait and she could pick up objects using her full hand. She did not have any language and little understanding. She did recognize her close family but paid little attention to the milieu around her. Muscle biopsy showed slightly reduced ATP production with several substrates. She is now 4 years old, still seizure free on treatment, and with little development as compared to 2 years of age. Trio genome sequencing identified a homozygous mutation in the *PPFIBP1* gene c.403C>T, p.(Arg135*). Her parents were both confirmed heterozygous for the variant by sanger sequencing.

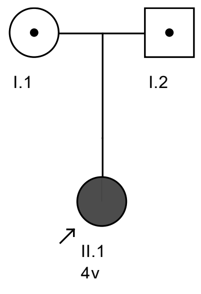

Individual 8

The female individual was born to unaffected consanguineous parents of Iranian origin. In the pedigree, a maternal aunt with developmental delay, seizures and congenital heart defects was described who deceased and was not molecularly evaluated. During pregnancy, the mother was affected by a urinary tract infection, a renal stone and headache. Individual 8 was born at 37+6 gestational weeks with a weight of 2600 g (-1.47 SD), a length of 50 cm (0.46 SD) and an OFC of 33 cm (-0.74 SD). As she had

a congenital coronary fistula, mitral regurgitation and cardiomegaly, she underwent angiography for closure of the fistula during neonatal period.

At the age of three years she presented with normal height (98 cm [0.77 SD]), a body weight of 11 kg (-1.85 SD) and progressive low head circumference (-1.78 SD, 3.8th percentile). She was affected by profound developmental delay, was not able to sit independently, did not speak and showed muscular hypotonia and nystagmus. Epilepsy started at the age of six months and she was affected by generalized tonic-clonic seizures. Antiepileptic treatment with topiramate, valproate and levetiracetam controlled the seizures and she was seizure free for 5 months. Further symptoms included mild scoliosis and an internal rotation of the right foot. MRI of the head at the age of six months revealed a severe disorder of gyration with bilateral parietal thickening of the cortex and pachygyria, severe gray matter heterotopia along the lateral ventricles wall, ventriculomegaly with irregular walls, and hyperintensity and paucity of the white matter

Singleton exome sequencing revealed a homozygous nonsense variant in *PPFIBP1* (NM_003622.4): c.1417_1427del, p.(Ala473Lysfs*20) that was found heterozygous in the parents and in the unaffected brother by Sanger sequencing.

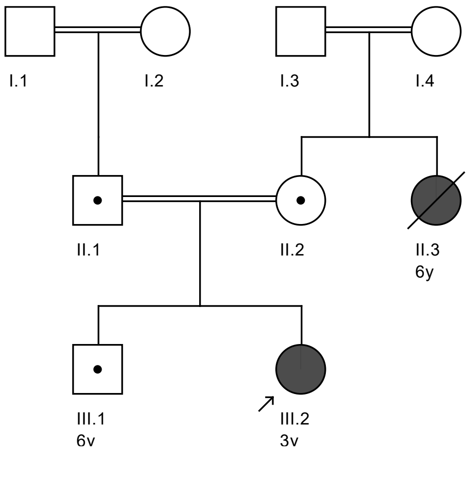

Individual 9

The male individual was born to unaffected parents. In the pedigree, low vision, disability due to seizures, congenital heart disease and dwarfism have been reported. Pregnancy was uneventful except for the umbilical cord being wrapped around the fetus' neck leading to preterm labour. He was born at

38 gestational weeks with a weight of 1780g (-3.79 SD), length of 40 cm (-5.22 SD) and microcephaly with an OFC of 31 cm (-2.73 SD).

At the last assessment at the age of 2 years and 6 months, he presented with severe ID, absent speech, spastic tetraplegia with no sphincter control and blindness. He was able to sit independently after occupational therapy, but unable to walk. Seizures occurred daily and started at the age of two months as gaze deviation and epileptic spasms and later frequently affected vision. He was treated with CZP and VPA and he was seizure free for a few months. He died at the age of 3.8 years.

Singleton exome sequencing revealed a homozygous nonsense variant in *PPFIBP1* (NM_003622.4): c.1300C>T, p.(Gln434*). Exome sequencing also identified a heterozygous suspicious variant in *ACADS* (MIM: 606885) but so far, only bi-allelic variants were described to cause ACADS deficiency (MIM: 201470) and the variant thus was not deemed causative for his condition. His parents were phenotypically normal.

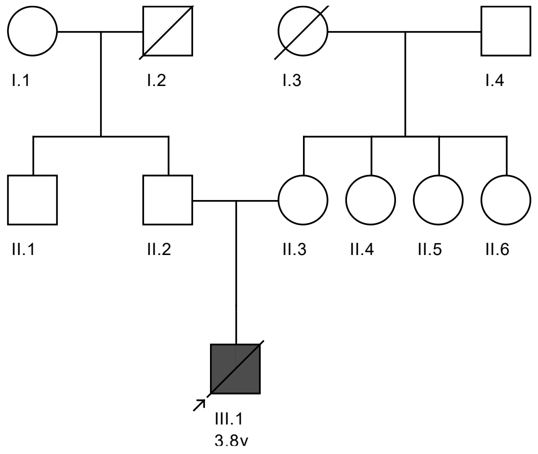

Individual 10

The male individual was born to healthy consanguineous parents of Egyptian origin. A similarly affected sister has passed away before. He was born at term (38 GW) with a weight of 3000 g (-0.73 SD) and microcephaly. He was admitted to neonatal intensive care unit as febrile convulsion started in the first week of life. In the course, epilepsy manifested with daily focal myoclonic seizures and epileptic spasms and hypsarrhythmia was seen on EEG. Seizures were controlled by medication with Valproate and Diazepam, although seizures relapsed once, but could be re-controlled with add-on medication. At the

age of one year and two months he presented with severe global developmental delay. At that time, he did not show social smile, was not able to sit independently and did not vocalise. Furthermore, he was affected by muscular hypotonia, right eye ptosis, left nystagmus, left iris coloboma and diffuse chorioretinal degeneration. MRI revealed bilateral symmetrical supratentorial abnormal myelination in the periventricular white matter, corona radiata and centrum semiovale, as well as a hypoplastic corpus callosum and a mildly ectatic ventricular system.

Singleton exome sequencing revealed a homozygous nonsense variant c.2629C>T, p.(Arg877*) in *PPFIBP1* (NM_003622.4). The parents were phenotypically normal and heterozygous by Sanger sequencing.

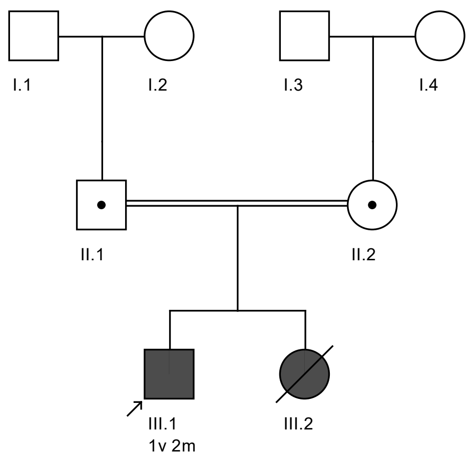

Individual 11

The male patient is the only child of healthy consanguineous Egyptian parents. The parents are first cousins. He was born at term by CS after an otherwise uneventful pregnancy with a birth weight of 2500 g (-1.9 SD, 3rd percentile). No documents were available for the birth length, OFC and Apgar score. First focal seizures started at age 2 weeks which initially were well-controlled by levetiracetam. At the age of 5 months, seizures progressed into myoclonic jerks five to seven times a day and clonazepam was started and later valproate was added. Despite these multiple antiepileptic drugs, his seizures were partially controlled on the maximum dosing of these drugs He was referred to the clinical genetics department at that age for genetic counselling. On physical examination, the weight was 8.3 kg (0.91 SD), length was 65 cm (-0.43 SD), and head circumference was 38 cm (-3.77 SD). Dysmorphic features

included bitemporal hollowing, wide nasal ridge, antiverted nostrils, low set ears and short neck. The patient showed global developmental delay; he could not recognize his parents, had no head control and did not show focusing, vocalizing, or smiling.

Neurologic examination demonstrated head lag, hypotonia, brisk reflexes, intermittent dystonic posture and nystagmus. Ophthalmologic examination showed bilateral primary optic atrophy. EEG showed occasional runs of spike slow waves. CT scans showed calcifications in the internal capsule, basal ganglia, white matter and periventricular. Brain-MRI displayed cortical atrophy, deep Sylvian fissures, mild ventriculomegaly, prominent basal ganglia, a hypoplastic corpus callosum, and retrocerebellar and bitemporal arachnoid cysts.

Singleton exome sequencing revealed a homozygous nonsense variant c.1468C>T, p.(Gln490*) in *PPFIBP1* (NM_003622.4). The parents were tested heterozygous for the variant by Sanger sequencing.

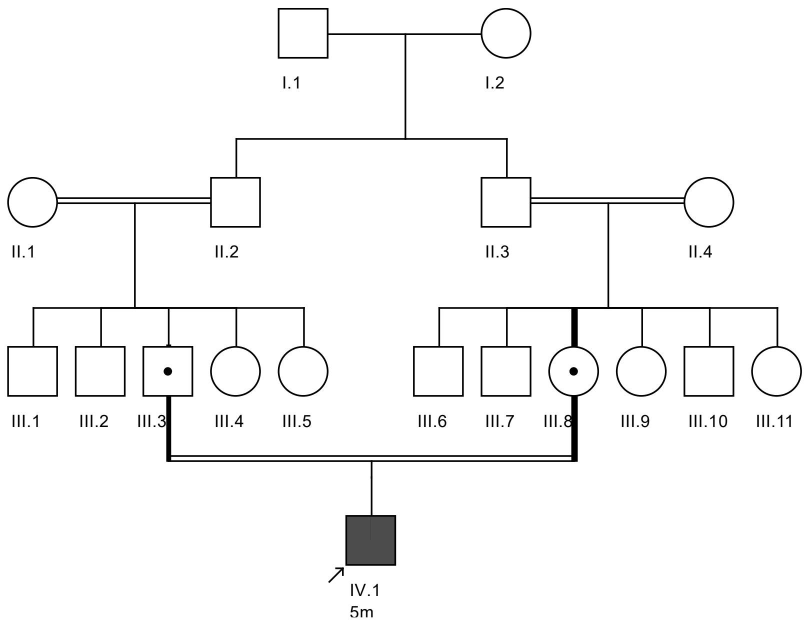

Individual 12

The female individual (V.I) was born at term after an uneventful pregnancy to unaffected and second-degree cousins of Syrian origin. At birth respiratory distress was reported but no mechanical ventilation was needed. From infancy, she was noted to have poor sucking with feeding difficulties, truncal hypotonia, and severe developmental delay. At 6 months, she developed generalized and focal seizures.

She became seizure-free with polytherapy including phenobarbital, valproic acid and carbamazepine. EEG during sleep showed slowing of background activity on the left posterior regions and epileptiform abnormalities on bi-hemispheric anterior regions (left> right). At her first evaluation at the age of 3 years and 9 months, her weight was 12.9 Kg (-1.4 SD), her length 90 cm (-2.6 SD) and her occipitofrontal circumference was 38.5 cm (-7.5 SD). She was noted to have dysmorphic features including sloping forehead, low-set hairline, and synophris. At the age of 5 years and 11 months, she presented with short stature (96 cm [-3.67 SD]), severe microcephaly (42 cm [-8.5 SD]) and low weight (13.2 kg [-3.1 SD]). She had profound intellectual disability with absent speech, truncal hypotonia being unable to sit or walk independently, dyskinetic movements and frequent stereotypic movements (e.g., hand-to-mouth).

A brain MRI performed at the age of five years revealed leukoencephalopathy with paucity of the white matter, ventriculomegaly with irregular walls of the lateral ventricles and suspected periventricular microcalcifications. It also showed abnormalities of cortical gyration including bilateral frontal polymicrogyria and thickening of the temporoparietal cortex, especially of the right insula.

Trio exome sequencing revealed a homozygous frameshift variant in *PPFIBP1* (NM_003622.4): c.2654del, p.(Tyr885Leufs*4). Her parents (IV.2; III.4) were phenotypically normal and heterozygous for this variant. Sanger sequencing showed the heterozygous variant only in one of the healthy sisters (V.3). One daughter (IV.1) of the maternal grandfather (III.2) suffered from severe encephalopathy.

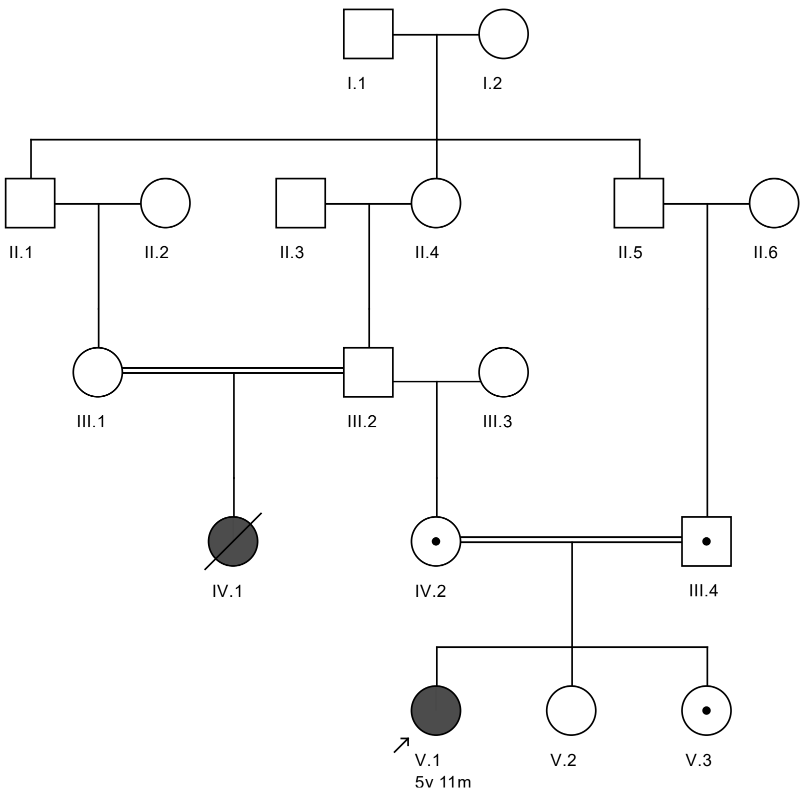

Fetus (family 13)

The clinical and molecular findings in the fetus are fully described in the main article.

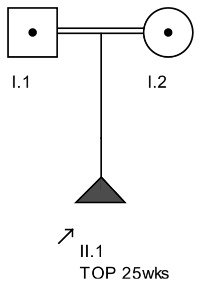

TOP = termination of pregnancy 1

Additional Case: Individual 14

In a four and a half year old male individual, a homozygous splice-site variant chr12:g.27682500T>C, NM_003622.4:c.2158+2T>C, p.? was identified by exome sequencing. The variant is predicted by splice AI to result in a disruption of the 3'-donor splice site of exon 23/30. Skipping of this exon would be out of frame. The variant is absent from gnomAD.

Exome sequencing also found a homozygous missense variant in the *WWOX* gene (MIM: 605131): NM_016373.4:c.900C>A, p.(Asn300Lys). This variant is absent from gnomAD and has a CADD score of 19.26. Bi-allelic missense variants in WWOX have been associated with autosomal recessive Spinocerebellar ataxia (MIM:614322) and bi-allelic LoF variants have been associated with developmental and epileptic encephalopathy (MIM:616211). Likely pathogenic and pathogenic bi-allelic missense variants have been reported in ClinVar. The *WWOX* variant has been classified as a variant of unknown significance (VUS) according to the guidelines of the ACMG.

The individual was born preterm at 32 gestational weeks due to a fetal heart rhythm disorder with a weight of 2000 g (0.59 SD) and an occipitofrontal circumference of 30.2 cm (0.55 SD). He later was affected by global developmental delay including delayed walking and sitting. At the age of 4 months, he developed refractory epilepsy including clonic seizures, upward gaze and epileptic spasms. Additionally, he showed strabismus and bilateral visual defects.

Given the VUS in the *WWOX* gene, two possible genetic diagnoses are plausible for this individual. To prevent this possibility from impacting the phenotypic description this Individual was not included in the overall phenotypic and genetic description.

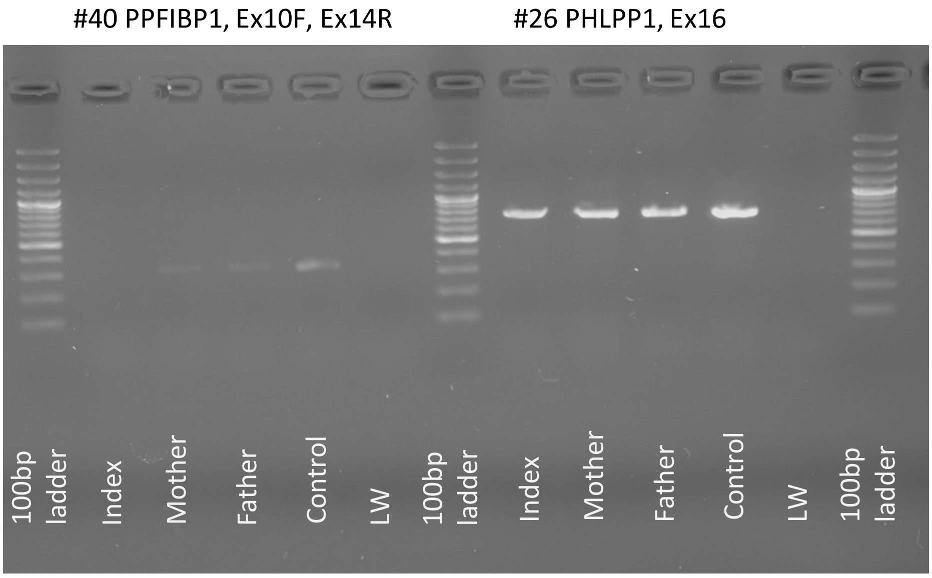

Figure S1. Gel electrophoresis of cDNA from individual 1, his parents, and a control sample of *PPFIBP1* (left) and a reference gene (*PHLPP1*, Ex 16; right). For the region *PPFIBP1*, spanning Ex10F-Ex14R encompassing the variant NM_003622.4: c.1146+1G>A, weaker bands compared to the control can be seen in the heterozygous parents and no band is seen in the homozygous index. This observation supports nonsense-mediated mRNA decay (NMD) of the mutated allele. For the reference gene, the bands have similar intensities in all samples.

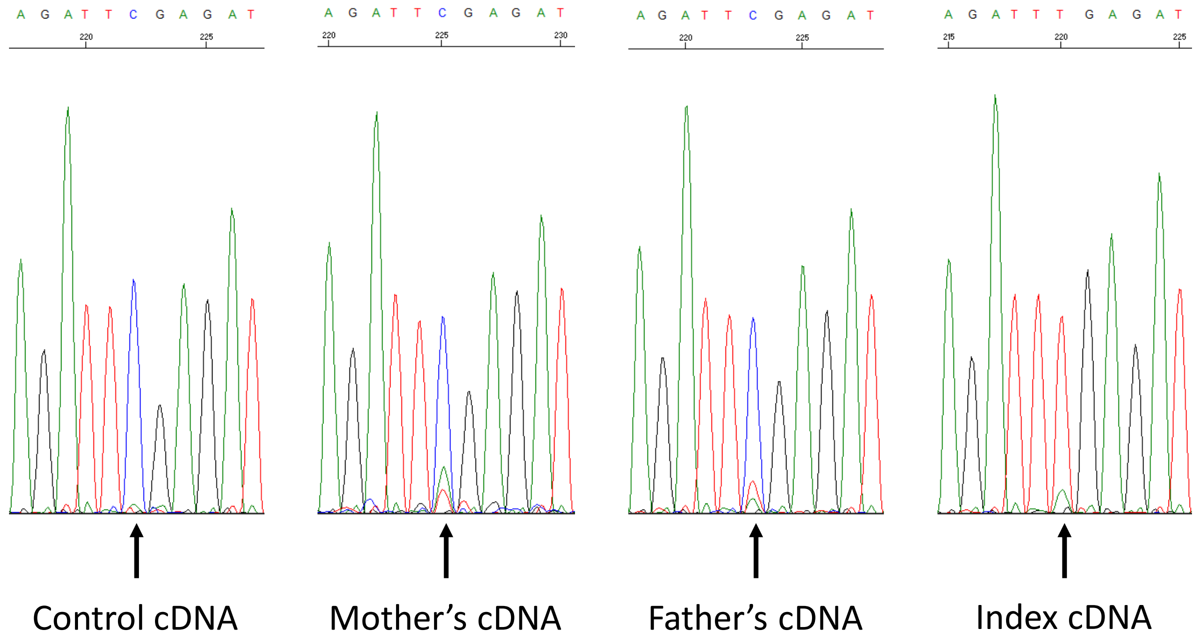

Figure S2. Electropherogram showing the sequence surrounding the c.403C>T variant at the cDNA level from a control sample, the parents who are heterozygous for the variant and individual 7 who is homozygous for the variant. The arrow indicates the base that is changed at the genomic level. Both parents show reduced signals of the mutated allele compared to the wild-type allele which suggests NMD of the aberrant mRNA.

**Table S1**								
**Individual**	**Chr**	**Genomic position** (GRCh38)	**cDNA** (NM_003622.4)	**Protein** (NP_003613.4)	**Allelic state**	**Predicted effect**	**ACMG criteria**	**Classification**
1	12	g.27667321G>A	c.1146+1G>A	p.?	homozygous	Loss of 5'-donor splice site of exon 13	PVS1, PM2_Supporting, PM3_Supporting	Pathogenic
2, 12	12	g.27689172del	c.2654del	p.(Tyr885Leufs*4)	homozygous	Nonsense mediated mRNA decay	PVS1, PM2_Supporting, PM3_Supporting	Pathogenic
3-1, 3-2, 3-3, 4	12	g.27673815_27673816del	c.1368_1369del	p.(Glu456Aspfs*3)	homozygous	Nonsense mediated mRNA decay	PVS1, PM2_Supporting, PM3_Supporting, PP1_Moderate	Pathogenic
5-1, 5-2	12	g.27688340C>T	c.2413C>T	p.(Arg805*)	homozygous	Nonsense mediated mRNA decay	PVS1, PM2_Supporting, PM3_Supporting	Pathogenic
6-1, 6-2, 11	12	g.27676485C>T	c.1468C>T	p.(Gln490*)	homozygous	Nonsense mediated mRNA decay	PVS1, PM2_Supporting, PM3_Supporting	Pathogenic
7	12	g.27647774C>T	c.403C>T	p.(Arg135*)	homozygous	Nonsense mediated mRNA decay	PVS1, PM2_Supporting, PM3_Supporting	Pathogenic
8	12	g.27676434_27676444del	c.1417_1427del	p.(Ala473Lysfs*20)	homozygous	Nonsense mediated mRNA decay	PVS1, PM2_Supporting, PM3_Supporting	Pathogenic
9	12	g.27672464C>T	c.1300C>T	p.(Gln434*)	homozygous	Nonsense mediated mRNA decay or inframe deletion of exon 15	PVS1_Moderate, PM2_Supporting, PM3_Supporting	Uncertain significance**

**Table S1 continued**								
10	12	g.27689147C>T	c.2629C>T	p.(Arg877*)	homozygous	Nonsense mediated mRNA decay	PVS1, PM2_Supporting, PM3_Supporting	Pathogenic
Fetus (13)	12	g.27682633G>T	c.2177G>T	p.(Gly726Val)	homozygous	Missense change predicted to severely disturb topology of the SAM-domain region	PM2_Supporting PM3_Supporting PP3	Uncertain significance ***
Additional case (14)	12	g.27682500T>C	c.2158+2T>C	p.?	homozygous	Loss of 5'-donor splice site of exon 23	PVS1 PM2_Supporting, PM3_Supporting	Pathogenic

* Although ACMG criteria are formally not perfectly suited for the description of novel disease-associated genes, we classified all variants from this study to simplify further use of the data.

** Since SpliceAI predicts a loss of the acceptor and donor splice sites of exon 15/30 with moderate probabilities, an in-frame deletion of exon 15/30 removing less than 10% of the protein cannot be ruled out. Thus, the PVS1 criterion is weighted at a moderate level and this variant can only be classified as of uncertain significance. Due to the high clinical overlap to the rest of the cohort, this variant is deemed causative despite being classified as uncertain.

***Due to the high clinical overlap to the rest of the cohort, this variant is deemed causative despite being classified as uncertain.

Table S1. Variant information and classification according to the ACMG criteria[1,*]. Variant descriptions were validated according to the HGVS nomenclature.

**Ind.**	**Genomic position** (GRCh38)	**cDNA**	**CADD-v6**[2]	**SpliceAI**[3]	**MaxEntScan**[4]	**NNSPLICE**[5]	**Nucleotide conservation**	**gnomAD**[6]
1	chr12:g.27667321	c.1146+1G>A, p.?	35	0.95	1	1	High	0

Table S2. *In silico* prediction of the splice variant *PPFIBP1* (NM_003622.4). Red color represents a very high probability of the variant to be damaging for each aspect in the table.

**Ind.**	**Genomic position** (GRCh38)	**cDNA**	**CADD-v6**[2]	**REVEL**[7]	**Mutation Taster**[8]	**M-CAP 1.3**[9]	**Polyphen 2 v2.2.2**[10]	**GERP++**[11]	**AA conservation**	**gnomAD**[6]
Fetus (11)	chr12:27682633	c.2177G>T, p.(Gly726Val)	24.6	0,609 (LDC)	D	0.063 (PoP)	PrD	4,46	High	0

Table S3. *In silico* prediction of the missense variant and conservation of the affected amino acid in *PPFIBP1*(NM_003622.4). Red color represents a very high probability of the variant to be damaging for each aspect in the table; LDC = likely disease causing; D = Deleterious; PoP = Possibly Pathogenic; PrD = Probably Damaging

Supplemental Methods

Use of GTEx Expression Data

To assess the relevance of the variants in different transcripts by using the expression data of GTEx v8 featuring Ensembl transcripts, the following assumptions have been made given the only minor differences in the untranslated regions of the transcripts. It has been assumed that NM_003622.4 which equals the Ensembl transcript ENST00000228425.11 also equals the version ENST00000228425.10. Also, it has been assumed that the transcript NM_001198915.2 equals ENST00000537927.5.

Supplemental sequencing methods

Individual 1

Trio exome sequencing of the index and the parents was performed at the Institute of Human Genetics at the University of Leipzig Medical Center. Library preparation was done using the Nextera DNA Flex Pre-Enrichment LibraryPrep with Illumina Nextera DNA UD Indexes by Illumina (San Diego, CA, USA). Target enrichment was achieved by using the Human Core Exome hybridization probes from Twist Bioscience (San Francisco, CA, USA). Paired-end Next-Generation-Sequencing was then performed on a NovaSeq 6000 Instrument (Illumina), located at the facilities of GeneWIZ (Leipzig, Germany), using an S1 Reagent Kit (300 cycles) by Illumina. Coverage of more than 20x has been achieved in more than 95 % of target sequences in all family members.

Analysis of the raw data, variant annotation and prioritization were performed using the software Varfeed (Limbus, Rostock, Germany) and Varvis (Limbus, Rostock, Germany). Variants were prioritized based on the mode of inheritance, impact on the gene product, minor allele frequency and *in silico* predicted pathogenicity. For research evaluation of variants in potential candidate genes, we also considered the biological function of the gene product with regards to neurodevelopment, mutational constraint parameters[6] (i.e. observed/expected - ratio, pLI-Score, Z-Score), expression patterns of the gene[14], insights on animal models and further aspects such as protein interaction networks and the function of similar or related proteins.

Individual 2

Trio exome sequencing of the index case and the parents was performed as previously described.[15] Library preparation was done using the Nextera DNA Flex Pre-Enrichment LibraryPrep with Illumina Nextera DNA UD Indexes by Illumina (San Diego, CA, USA). Target enrichment was achieved by using a modified version of the Human Core Exome hybridization probes from Twist Bioscience (San Francisco, CA, USA) complemented with additional custom probes. Paired-end Next-Generation-Sequencing (2x100 bp) was then performed on a NovaSeq 6000 Instrument (Illumina), at IntegraGen SA (Evry, France). Coverage of more than 20X has been achieved in more than 97 % of target sequences in the index case. Analysis of the raw data, variant annotation and prioritization were performed as previously described.[15]

Individuals 3-1, 3-2 and 3-3

Exome sequencing and variant prioritization supported by autozygome analysis were performed as previously described.[16,17]

Individual 4

Exome sequencing of the index and targeted sequencing on both DNA strands of the relevant *PPFIBP1* region for the parents was performed at CENTOGENE (the rare disease company) (Rostock, Germany). Double stranded DNA capture baits against approximately 36.5 Mb of the human coding exome (targeting >98% of the coding RefSeq from the human genome build GRCh37/hg19) are used to enrich target regions from fragmented genomic DNA with the Twist Human Core Exome Plus kit by Twist Bioscience (San Francisco, CA, USA). The generated library is sequenced on an Illumina platform (San Diego, CA, USA) to obtain at least 20x coverage depth for >98% of the targeted bases. An in-house bioinformatics pipeline, including read alignment to GRCh37/hg19 genome assembly, variant calling, annotation and comprehensive variant filtering is applied. All variants with minor allele frequency

(MAF) of less than 1% in gnomAD[6] database, and disease-causing variants reported in HGMD[18], in ClinVar[19] or in CentoMD® by CENTOGENE are considered. The investigation for relevant variants is focused on coding exons and flanking +/-20 intronic nucleotides of genes with clear gene-phenotype evidence (based on OMIM® information). All potential modes of inheritance patterns are considered. In addition, provided family history and clinical information are used to evaluate identified variants with respect to their pathogenicity and causality. Variants are categorized into five classes (pathogenic; likely pathogenic; VUS; likely benign; benign). All variants related to the phenotype of the patient are reported. CENTOGENE has established stringent quality criteria and validation processes for variants detected by next-generation sequencing. Variants with low quality and/or unclear zygosity are confirmed by orthogonal methods. Consequently, a specificity of >99.9% for all reported variants is warranted.

Individuals 5-1, 5-2, 8 and 10

Exome sequencing and variant annotation were performed as previously described.[20]

Individuals 6-1, 6-2 and 11

Exome sequencing (ES) of the proband was performed using a TruSeq DNA PCR-free library preparation method by Illumina (San Diego, CA, USA) and subsequent sequencing on a NovaSeq 6000 sequencing instrument (Illumina) to a sequencing depth of 30x median coverage. The resulting ES data was analysed using the Mutation Identification Pipeline (MIP) as previously described.[21] Segregation of the variant identified in *PPFIBP1* was carried out by PCR amplification of genomic DNA and subsequent Sanger sequencing using the BigDye version 3.1 sequencing kit (Applied Biosystems, Foster City, CA, USA) on a 3500 Genetic Analyzer (Applied Biosystems).

Individual 7

Genome sequencing of the proband and her parents was performed at the Science for Life Laboratory, Clinical Genomics facility, Stockholm, using a TruSeq DNA PCR-free library preparation method by Illumina (San Diego, CA, USA) and subsequent sequencing on a NovaSeq 6000 sequencing instrument (Illumina) to a sequencing depth of 30x median coverage. The resulting WGS data was analyzed using the Mutation Identification Pipeline (MIP) as previously described.[21] Confirmation of the variant observed in *PPFIBP1* was carried out by PCR amplification of genomic DNA and subsequent Sanger sequencing using the BigDye version 3.1 sequencing kit (Applied Biosystems, Waltham, Massachusetts, USA) on a 3500xl Genetic Analyzer (Applied Biosystems).

Individual 9

Exome sequencing of the patient was performed at the Macrogen company (Seoul, South Korea) using Agilent SureSelect V6 post capture kit with a NovaSeq 6000 instrument by Illumina (San Diego, CA, USA). Target regions have a mean read depth of 100X.

Analysis of the raw data was performed using BWA and GATK packages. Variant annotation and prioritization were done using an in-house pipeline of the Palindrome lab; briefly this pipeline uses an ACMG based algorithm and an in-house database for variant prioritization. Clinically significant variants are curated manually.

Individual 12

Genomic DNA was extracted from peripheral blood leukocytes using standard protocols. For library preparation we followed the manufacturer's instructions (SureSelectQXT Automated Target Enrichment for the Illumina Platform, Protocol Version B0, November 2015, Agilent Technologies, Santa Clara, CA, USA). Target enrichment for trio exome sequencing was achieved by using the SureSelect Human All Exon v7 (Agilent Technologies, Santa Clara, CA, USA). The libraries were sequenced using the NovaSeq 6000 system performing paired-end runs covering at least 2x150nt.

(Illumina Inc., San Diego, CA, USA). The generated sequences were analyzed using an in-house pipeline designed to automate the analysis workflow. The average exome coverage of the target bases of at least 100X with 90% of the bases covered by at least 40 reads. Direct Sanger Sequencing using specific primers was performed using the BigDye version 3.1 sequencing kit (Applied Biosystems, Waltham, Massachusetts, USA) on a 3500xl Genetic Analyzer (Applied Biosystems).

Fetus (Family 13)

Trio exome sequencing of the index and the parents was performed at Laboratoire Biomnis-Eurofins (Lyon, France). Library preparation was done using the Twist Library Preparation from Twist Bioscience (San Francisco, CA, USA). Target enrichment was achieved by using the Twist Human Core Exome hybridization probes from Twist Bioscience. Paired-end Next-Generation-Sequencing was then performed on a NextSeq500 Instrument, using an a NextSeq 500/550 High Output Kit v2 (2x75pb, paired-end) by Illumina (San Diego, CA, USA). Coverage of more than 30x has been achieved in more than 97 % of target sequences in all family members.

Analysis of the raw data, variant annotation and prioritization were performed using the software SeqOne (Montpellier, France).

Sanger verification has been performed with the BigDye v3.1 kit followed by a purification with the BigDye X-terminator from Life Technologies. The electrophoresis has been done on the SeqStudio instrument from Applied Biosystems (Waltham, Massachusetts, USA).

RNA analysis

Family 1 (Figure S1)

RNA was extracted using PAXgene Blood RNA Kit (Qiagen) with an input volume of 9 ml. cDNA synthesis was done using PrimeScript RT Master Mix (TaKaRa) starting with a total RNA amount of 1 µg. PCR was done using DreamTaq Hot Start DNA Polymerase (ThermoScientific) and the following oligos: PPFIBP1 (NM_001198915.2), Ex10_A_F, 5’ GCTGCTCAATTCCAGTTCCA 3’; Ex14_A_R,

5’ TGCTGGACTTCTGCAGACTT 3’ generating a 332 bp PCR product and as a reference PHLPP1 (NM_194449.3), Ex16_A_F, GGAATGTGGAGGTGCCCTAC, Ex16_A_R, gggaggaaagatgccaggac, generating a 713 bp product. Sanger sequencing was performed using PCR product after clean-up using Exo SAP-IT Express (Applied Biosystems) and the oligos as described above. Sequencing reaction was done using Big Dye Terminator v3.1 Cycle Sequencing kit and Big Dye Terminator v1.1, v3.1 5x Sequencing Buffer. Final clean up was done using ethanol. Electrophoresis was performed on a ABI3500 capillary electrophoresis instrument (Thermo Scientific).

Family 7 (Figure S2)

RT-PCR was performed on RNA isolated from the patient and parents blood, using the High Capacity cDNA Reverse Transcription Kit (Applied Biosystems, Thermo Fisher) and the following M13-tagged primers: PPFIBP1_cDNA_4F:TGGATTGTTAGAGATGATGG and PPFIBP1_cDNA_8R:GAAGTCTCTCACTGTCCATT. Subsequent sequencing of the PCR products was carried out with M13 primers, using the BigDye version 3.1 sequencing kit (Applied Biosystems) on a 3500xl Genetic Analyzer (Applied Biosystems) with alignment to the reference sequence NM_003622.

Supplemental References

1. Richards, S., Aziz, N., Bale, S., Bick, D., Das, S., Gastier-Foster, J., Grody, W.W., Hegde, M., Lyon, E., Spector, E., et al. (2015). Standards and guidelines for the interpretation of sequence variants: a joint consensus recommendation of the American College of Medical Genetics and Genomics and the Association for Molecular Pathology. Genetics in Medicine *17*, 405–424.

2. Rentzsch, P., Schubach, M., Shendure, J., and Kircher, M. (2021). CADD-Splice—improving genome-wide variant effect prediction using deep learning-derived splice scores. Genome Med *13*, 31.

3. Jaganathan, K., Kyriazopoulou Panagiotopoulou, S., McRae, J.F., Darbandi, S.F., Knowles, D., Li, Y.I., Kosmicki, J.A., Arbelaez, J., Cui, W., Schwartz, G.B., et al. (2019). Predicting Splicing from Primary Sequence with Deep Learning. Cell *176*, 535-548.e24.

4. Yeo, G., and Burge, C.B. (2004). Maximum Entropy Modeling of Short Sequence Motifs with Applications to RNA Splicing Signals. Journal of Computational Biology *11*, 377–394.

5. Jian, X., Boerwinkle, E., and Liu, X. (2014). In silico prediction of splice-altering single nucleotide variants in the human genome. Nucleic Acids Research *42*, 13534–13544.

6. Karczewski, K.J., Francioli, L.C., Tiao, G., Cummings, B.B., Alföldi, J., Wang, Q., Collins, R.L., Laricchia, K.M., Ganna, A., Birnbaum, D.P., et al. (2020). The mutational constraint spectrum quantified from variation in 141,456 humans. Nature *581*, 434–443.

7. Ioannidis, N.M., Rothstein, J.H., Pejaver, V., Middha, S., McDonnell, S.K., Baheti, S., Musolf, A., Li, Q., Holzinger, E., Karyadi, D., et al. (2016). REVEL: An Ensemble Method for Predicting the Pathogenicity of Rare Missense Variants. The American Journal of Human Genetics *99*, 877–885.

8. Schwarz, J.M., Rödelsperger, C., Schuelke, M., and Seelow, D. (2010). MutationTaster evaluates disease-causing potential of sequence alterations. Nat Methods *7*, 575–576.

9. Jagadeesh, K.A., Wenger, A.M., Berger, M.J., Guturu, H., Stenson, P.D., Cooper, D.N., Bernstein, J.A., and Bejerano, G. (2016). M-CAP eliminates a majority of variants of uncertain significance in clinical exomes at high sensitivity. Nat Genet *48*, 1581–1586.

10. Adzhubei, I., Jordan, D.M., and Sunyaev, S.R. (2013). Predicting functional effect of human missense mutations using PolyPhen-2. Curr Protoc Hum Genet *Chapter 7*, Unit7.20.

11. Cooper, G.M., Stone, E.A., Asimenos, G., NISC Comparative Sequencing Program, Green, E.D., Batzoglou, S., and Sidow, A. (2005). Distribution and intensity of constraint in mammalian genomic sequence. Genome Res *15*, 901–913.

12. Altschul, S.F., Madden, T.L., Schäffer, A.A., Zhang, J., Zhang, Z., Miller, W., and Lipman, D.J. (1997). Gapped BLAST and PSI-BLAST: a new generation of protein database search programs. Nucleic Acids Res *25*, 3389–3402.

13. Altschul, S.F., Wootton, J.C., Gertz, E.M., Agarwala, R., Morgulis, A., Schäffer, A.A., and Yu, Y.-K. (2005). Protein database searches using compositionally adjusted substitution matrices. FEBS J *272*, 5101–5109.

14. Lonsdale, J., Thomas, J., Salvatore, M., Phillips, R., Lo, E., Shad, S., Hasz, R., Walters, G., Garcia, F., Young, N., et al. (2013). The Genotype-Tissue Expression (GTEx) project. Nat Genet *45*, 580–585.

15. Tran Mau-Them, F., Duffourd, Y., Vitobello, A., Bruel, A., Denommé-Pichon, A., Nambot, S., Delanne, J., Moutton, S., Sorlin, A., Orphanomix Physician's Group, et al. (2021). Interest of exome

sequencing trio-like strategy based on pooled parental DNA for diagnosis and translational research in rare diseases. Molec Gen & Gen Med *9*,.

16. Shaheen, R., Maddirevula, S., Ewida, N., Alsahli, S., Abdel-Salam, G.M.H., Zaki, M.S., Tala, S.A., Alhashem, A., Softah, A., Al-Owain, M., et al. (2019). Genomic and phenotypic delineation of congenital microcephaly. Genetics in Medicine *21*, 545–552.

17. Saudi Mendeliome Group (2015). Comprehensive gene panels provide advantages over clinical exome sequencing for Mendelian diseases. Genome Biol *16*, 134.

18. Stenson, P.D., Mort, M., Ball, E.V., Chapman, M., Evans, K., Azevedo, L., Hayden, M., Heywood, S., Millar, D.S., Phillips, A.D., et al. (2020). The Human Gene Mutation Database (HGMD®): optimizing its use in a clinical diagnostic or research setting. Hum Genet *139*, 1197–1207.

19. Landrum, M.J., Lee, J.M., Benson, M., Brown, G.R., Chao, C., Chitipiralla, S., Gu, B., Hart, J., Hoffman, D., Jang, W., et al. (2018). ClinVar: improving access to variant interpretations and supporting evidence. Nucleic Acids Research *46*, D1062–D1067.

20. Makrythanasis, P., Maroofian, R., Stray-Pedersen, A., Musaev, D., Zaki, M.S., Mahmoud, I.G., Selim, L., Elbadawy, A., Jhangiani, S.N., Coban Akdemir, Z.H., et al. (2018). Biallelic variants in KIF14 cause intellectual disability with microcephaly. Eur J Hum Genet *26*, 330–339.

21. Stranneheim, H., Lagerstedt-Robinson, K., Magnusson, M., Kvarnung, M., Nilsson, D., Lesko, N., Engvall, M., Anderlid, B.-M., Arnell, H., Johansson, C.B., et al. (2021). Integration of whole genome sequencing into a healthcare setting: high diagnostic rates across multiple clinical entities in 3219 rare disease patients. Genome Med *13*, 40.

22. Mencacci, N.E., Kamsteeg, E.-J., Nakashima, K., R'Bibo, L., Lynch, D.S., Balint, B., Willemsen, M.A.A.P., Adams, M.E., Wiethoff, S., Suzuki, K., et al. (2016). De Novo Mutations in PDE10A Cause Childhood-Onset Chorea with Bilateral Striatal Lesions. Am J Hum Genet *98*, 763–771.
