# Supplementary material for: Bi-allelic loss-of-function variants in PPFIBP1 cause a neurodevelopmental disorder with microcephaly, epilepsy, and periventricular calcifications
Source: Am J Hum Genet. 2022 Jul 12;109(8):1421–35. doi: 10.1016/j.ajhg.2022.06.008 (PMC9388382; doi:10.1016/j.ajhg.2022.06.008)
Supplement: Document S2. Article plus supplemental information [file mmc3.pdf]

Bi-allelic loss-of-function variants in *PPFIBP1* cause a neurodevelopmental disorder with microcephaly, epilepsy, and periventricular calcifications

Authors

Erik Rosenhahn, Thomas J. O'Brien, Maha S. Zaki, ..., Andre E.X. Brown, Reza Maroofian, Konrad Platzer

Correspondence

andre.brown@lms.mrc.ac.uk (A.E.X.B.),
konrad.platzer@medizin.uni-leipzig.de (K.P.)

PPFIBP1 **encodes for liprin-β1, a presynaptic scaffold. Here, we describe 16 individuals from 12 unrelated families with bi-allelic loss-of-function variants in** ***PPFIBP1*** **sharing a phenotype of severe developmental delay, epilepsy, microcephaly, and cerebral calcifications. A** ***C. elegans hlb-1*** **knockout model demonstrates behavioral abnormalities to underscore gene-disease causality.**

Rosenhahn et al., 2022, The American Journal of Human Genetics *109*, 1421–1435

August 4, 2022 © 2022 The Authors.

https://doi.org/10.1016/j.ajhg.2022.06.008

CellPress

Bi-allelic loss-of-function variants in *PPFIBP1* cause a neurodevelopmental disorder with microcephaly, epilepsy, and periventricular calcifications

Erik Rosenhahn,[1,38] Thomas J. O'Brien,[2,38] Maha S. Zaki,[4] Ina Sorge,[5] Dagmar Wieczorek,[6] Kevin Rostasy,[7] Antonio Vitobello,[8] Sophie Nambot,[9] Fowzan S. Alkuraya,[10,11] Mais O. Hashem,[10] Amal Alhashem,[11,12] Brahim Tabarki,[12] Abdullah S. Alamri,[13] Ayat H. Al Safar,[13] Dalal K. Bubshait,[13] Nada F. Alahmady,[14] Joseph G. Gleeson,[15,16] Mohamed S. Abdel-Hamid,[17] Nicole Lesko,[18,19] Sofia Ygberg,[18,19,20] Sandrina P. Correia,[19,21] Anna Wredenberg,[18,19] Shahryar Alavi,[22,23] Seyed M. Seyedhassani,[24] Mahya Ebrahimi Nasab,[24] Haytham Hussien,[25] Tarek E.I. Omar,[25] Ines Harzallah,[26] Renaud Touraine,[26] Homa Tajsharghi,[27] Heba Morsy,[28] Henry Houlden,[28] Mohammad Shahrooei,[29,30] Maryam Ghavideldarestani,[29] Ghada M.H. Abdel-Salam,[4] Annalaura Torella,[31,32] Mariateresa Zanobio,[31] Gaetano Terrone,[33] Nicola Brunetti-Pierri,[32,34] Abdolmajid Omrani,[35] Julia Hentschel,[1] Johannes R. Lemke,[1,36] Heinrich Sticht,[37] Rami Abou Jamra,[1] Andre E.X. Brown,[2,3,39,*] Reza Maroofian,[28,39] and Konrad Platzer[1,39,*]

Summary

PPFIBP1 encodes for the liprin-β1 protein, which has been shown to play a role in neuronal outgrowth and synapse formation in *Drosophila melanogaster.* By exome and genome sequencing, we detected nine ultra-rare homozygous loss-of-function variants in 16 individuals from 12 unrelated families. The individuals presented with moderate to profound developmental delay, often refractory early-onset epilepsy, and progressive microcephaly. Further common clinical findings included muscular hyper- and hypotonia, spasticity, failure to thrive and short stature, feeding difficulties, impaired vision, and congenital heart defects. Neuroimaging revealed abnormalities of brain morphology with leukoencephalopathy, ventriculomegaly, cortical abnormalities, and intracranial periventricular calcifications as major features. In a fetus with intracranial calcifications, we identified a rare homozygous missense variant that by structural analysis was predicted to disturb the topology of the SAM domain region that is essential for protein-protein interaction. For further insight into the effects of *PPFIBP1* loss of function, we performed automated behavioral phenotyping of a *Caenorhabditis elegans PPFIBP1/hlb-1* knockout model, which revealed defects in spontaneous and light-induced behavior and confirmed resistance to the acetylcholinesterase inhibitor aldicarb, suggesting a defect in the neuronal presynaptic zone. In conclusion, we establish bi-allelic loss-of-function variants in *PPFIBP1* as a cause of an autosomal recessive severe neurodevelopmental disorder with early-onset epilepsy, microcephaly, and periventricular calcifications.

[1]Institute of Human Genetics, University of Leipzig Medical Center, 04103 Leipzig, Germany; [2]MRC London Institute of Medical Sciences, London W12 0NN, UK; [3]Faculty of Medicine, Institute of Clinical Sciences, Imperial College London, London SW7 2AZ, UK; [4]Clinical Genetics Department, Human Genetics and Genome Research Institute, National Research Centre, Cairo 12622, Egypt; [5]Department of Pediatric Radiology, University Hospital Leipzig, 04103 Leipzig, Germany; [6]Institute of Human Genetics, Medical Faculty and University Hospital Düsseldorf, Heinrich-Heine-University Düsseldorf, 40225 Düsseldorf, Germany; [7]Department of Pediatric Neurology, Children's and Adolescents' Hospital Datteln, Witten/Herdecke University, 58448 Witten, Germany; [8]UF6254 Innovation en Diagnostic Genomique des Maladies Rares, CHU Dijon Bourgogne, FHU translad, Génétique des Anomalies du Développement, INSERM UMR 1231, Université de Bourgogne-Franche Comté, 21070 Dijon, France; [9]Centre de Génétique et Centre de référence des Maladies rare, Anomalies du Développement et Syndromes Malformatifs, Hôpital d'Enfants, Centre Hospitalier Universitaire de Dijon, 21079 Dijon, France; [10]Department of Translational Genomics, Center for Genomic Medicine, King Faisal Specialist Hospital and Research Center, Riyadh 11211, Saudi Arabia; [11]Department of Anatomy and Cell Biology, College of Medicine, Alfaisal University, Riyadh 11211, Saudi Arabia; [12]Department of Pediatrics, Prince Sultan Military Medical City, Riyadh 12233, Saudi Arabia; [13]Department of Pediatrics, Imam Abdulrahman bin Faisal University, Dammam 34212, Saudi Arabia; [14]Biology Department, Imam Abdulrahman bin Faisal University, Dammam 34212, Saudi Arabia; [15]Department of Neurosciences, University of California, San Diego, La Jolla, CA 92093, USA; [16]Rady Children's Institute for Genomic Medicine, San Diego, La Jolla, CA 92093, USA; [17]Medical Molecular Genetics Department, Human Genetics and Genome Research Institute, National Research Centre, Cairo 12622, Egypt; [18]Department of Medical Biochemistry and Biophysics, Karolinska Institutet, 171 77 Stockholm, Sweden; [19]Centre for Inherited Metabolic Diseases, Karolinska University Hospital, 171 76 Stockholm, Sweden; [20]Neuropediatric Unit, Department of Women's and Children's Health, Karolinska University Hospital, 171 77 Stockholm, Sweden; [21]Department of Molecular Medicine and Surgery, Karolinska Institutet, 171 76 Stockholm, Sweden; [22]Department of Cell and Molecular Biology and Microbiology, Faculty of Biological Science and Technology, University of Isfahan, Isfahan, Iran; [23]Palindrome, Isfahan, Iran; [24]Dr. Seyedhassani Medical Genetic Center, Yazd, Iran; [25]Alexandria University Children's Hospital, Faculty of Medicine, Alexandria University, Alexandria 21526, Egypt; [26]Clinical, Chromosomal and Molecular Genetics Department, University Hospital Center, 42270 Saint-Étienne, France; [27]School of Health Sciences, Translational Medicine, University of Skövde, 541 28 Skövde, Sweden; [28]UCL Queen Square Institute of Neurology, University College London, London WC1N 3BG, UK; [29]Specialized Immunology Laboratory of Dr. Shahrooei, Sina Medical Complex, Ahvaz, Iran; [30]Department of Microbiology and Immunology, Clinical and Diagnostic Immunology, KU Leuven, 3000 Leuven, Belgium; [31]Department of Precision Medicine, University of Campania Luigi Vanvitelli, 80138 Naples, Italy; [32]Telethon Institute of Genetics and Medicine, 80078 Naples, Italy; [33]Child Neurology Unit, Department of Translational Medical Science, University of Naples Federico II, 80131 Naples, Italy; [34]Department of Translational Medicine, Section of Pediatrics, University of Naples Federico II, 80131 Naples, Italy; [35]Division of Clinical Studies, The Persian Gulf Nuclear Medicine Research Center, Bushehr University of Medical Sciences, Bushehr, Iran; [36]Center for Rare Diseases, University of Leipzig Medical Center, 04103 Leipzig, Germany; [37]Institute of Biochemistry, Friedrich-Alexander-Universität Erlangen-Nürnberg, 91054 Erlangen, Germany

[38]These authors contributed equally

[39]These authors contributed equally

*Correspondence: andre.brown@lms.mrc.ac.uk (A.E.X.B.), konrad.platzer@medizin.uni-leipzig.de (K.P.)

https://doi.org/10.1016/j.ajhg.2022.06.008.

© 2022 The Authors. This is an open access article under the CC BY license (http://creativecommons.org/licenses/by/4.0/).

Introduction

PPFIBP1 (GenBank: NM_003622.4; MIM: 603141) encodes for the PPFIA-binding protein 1, also known as liprin-β1. Liprin-β1 belongs to the liprin protein family whose members are characterized by a highly conserved N-terminal coiled-coil region and three adjacent C-terminal sterile alpha motifs (SAM domains) that form multiple protein-binding surfaces and allow for protein-protein interaction.[1–3] In mammals, the liprin family comprises four liprin-α proteins (liprin-α1–4) and two liprin-β proteins (liprin-β1 and -β2). Liprin-β1 has the ability to homodimerize and to heterodimerize with the homologous α-liprins.[1] In addition, liprin-β1 and liprin-α1 co-localize to the cell membrane and to the periphery of focal adhesions in fibroblast cell cultures (COS cells).[1,4] Liprin-α proteins are major scaffold proteins involved in synapse formation, synaptic signaling, and axonal transport processes via the assembly of large protein complexes.[5,6] Although yet to be confirmed, it has been suggested, that liprin-β1 could play a role in the regulation of liprin-α-mediated protein assemblies.[1,6,7] In line with this is the observation that liprin-β1 forms a ternary complex with liprin-α2 and CASK,[3] a presynaptic scaffolding protein essential for neurodevelopment.[8,9] A previous knockout model of the *PPFIBP1* ortholog *hlb-1* in *C. elegans* showed abnormal locomotion behavior. Furthermore, abnormal and decreased distribution of snb-1, an ortholog of human VAMP-family proteins involved in presynaptic vesicle release, increased presynaptic zone size, and resistance to the acetylcholinesterase inhibitor aldicarb indicated a role of *hlb-1* in the regulation of presynaptic function.[10] Pointing towards a role in neurodevelopment, null-allele mutants of the liprin-β1 ortholog liprin-β resulted in altered axon outgrowth and synapse formation of R7 photoreceptors and also reduced larval neuromuscular junction (NMJ) size in *D. melanogaster.*[7] Indeed, *PPFIBP1* has been proposed as a candidate gene for congenital microcephaly based on a single family, although this link remains tentative and requires independent confirmation.[11]

Here, we describe a cohort of 16 individuals with a neurodevelopmental disorder from 12 unrelated families harboring homozygous loss-of-function (LoF) variants and a fetus with a missense variant in *PPFIBP1*.

Subjects and methods

Patient recruitment and consent

The study was approved by the ethics committee of the University of Leipzig (402/16-ek). Written informed consent for molecular testing and permission for publication of the data was obtained from all individuals and/or their legal representatives by the referring physicians according to the guidelines of the ethics committees and institutional review boards of the respective institute.

All individuals were ascertained in the context of local diagnostic protocols followed by research evaluation of the sequencing data. The compilation of the cohort was supported by international collaboration and online matchmaking via GeneMatcher[12] in the case of families 2, 4, 5, 6, 7, 8, 9, 10, 11, 12, and 13. Family 3 was recently published as part of a larger cohort of individuals with congenital microcephaly.[11] In the context of our study, we describe the phenotype of affected individuals of family 3 in detail. Phenotypic and genotypic information were obtained from the referring collaborators with a standardized questionnaire.

Exome sequencing

Trio exome sequencing (ES) was performed for the affected individuals and the parents in the families 1, 2, 12, and 13 and quadruple ES was done in family 5 for the two affected siblings and their parents. Singleton ES was performed for the affected individuals of the families 3, 4, 6, 8, 9, 10 and 11. Family 7 was investigated by trio genome sequencing (GS) (see supplemental methods for further details).

Variant prioritization

We first analyzed single-nucleotide variants annotated in local and public mutation databases, as well as rare (minor allele frequency [MAF] $< 1\%$ in gnomAD) potentially protein-damaging variants in known disease-associated genes (e.g., by using *in silico* panels like MorbidGenes[13]). Synonymous variants and intronic variants with >20 bp distance to the adjacent exon border were not considered. Variants were prioritized on the basis of the plausibility of the mode of inheritance, zygosity, and phenotype with regard to gene-associated diseases, the presumed consequences on the gene product based on the variant type, allele frequencies in the general population and in-house databases, and *in silico* predicted pathogenicity. Because no pathogenic or likely pathogenic variants according to the guidelines of the American College of Medical Genetics (ACMG)[14] in known disease-associated genes could be found and the families gave consent for further research, we then evaluated the sequencing data in a research setting aiming to identify potentially causative variants in novel candidate genes. For this purpose, variants in potential candidate genes were prioritized according to the following parameters and an MAF of <1%. The plausibility of the mode of inheritance and zygosity was assessed with respect to the phenotype of parents and siblings and considering parental sequencing data in cases for which trio ES or GS was performed. For further prioritization, we obtained allele frequencies and mutational constraint parameters from gnomAD.[15] We specifically valued ultra-rare variants (MAF $< 0.01\%$), e.g., heterozygous missense variants in candidate genes with a Z score > 3.09 or heterozygous LoF variants in candidate genes with a pLI > 0.9 (pLI = probability of being loss-of-function intolerant) suggesting a selective constraint on these variant types in a population not affected by early-onset neurodevelopmental disorders. For homozygous candidate variants, lower selective constraint measures were used: Z score of >0.5 and LOEUF of <0.9 (LOEUF = loss-of-function observed/expected upper bound fraction). In addition, absence of homozygotes in the general population was a requirement. Several *in silico* predictions were considered for missense and splice-site variants (see next paragraph). Candidate genes that are at least moderately expressed in the central nervous system were prioritized, obtaining expression data from GTEx.[16] All variants in *PPFIBP1* described here were aligned to the human reference genome version GRCh38 (hg38) and to the transcript GenBank: NM_003622.4 (Ensembl: ENST00000228425.11) representing the transcript with the highest expression across all tissues[16] and the MANE Select v0.95 default transcript.[17] The pathogenicity of all

described variants was classified according to the guidelines of the ACMG[14] (Table S1).

In silico prediction

In silico predictions of the splice-site variant c.1146+1G>A (p.?) were assessed with CADD-v6,[18] SpliceAI,[19] MaxEntScan,[20] and NNSPLICE[21] (Table S2). *In silico* predictions of the missense variant c.2177G>T (p.Gly726Val) were assessed with CADD-v6,[18] REVEL,[22] Mutation Taster,[23] M-CAP 1.3,[24] and Polyphen 2[25] (Table S3).

Structural analysis

Structural analysis of the p.Gly726Val variant was performed on the basis of the crystal structure of murine PPFIBP1 (PDB: 3TAD, chain C[3]), which exhibits 97% sequence identity to its human ortholog in the region of the SAM domains (according to a BLAST sequence comparison with standard parameters[26]). The exchange was modeled with SwissModel[27] and RasMol[28] was used for structure analysis and visualization. For the identification of hydrophobic interactions, we used a 3.9 Å distance cutoff for as specified in Wallace et al., 1995.[29] Structural analysis for the other variants was not necessary because all are LoF variants and predicted to lead to a loss of protein.

Mutant *C. elegans* generation

The knockout worm model was designed and made by SunyBiotech (Fuzhou, Fujian, China) in their reference N2 background. CRISPR guide RNA was designed to target a large deletion (17,118 bp) starting close to the start codon and excising all exons from the gene. Deletions were confirmed by PCR.

Worm preparation

All strains were cultured on Nematode growth medium at 20°C and fed with *E. coli* (OP50) following standard procedure.[30] Synchronized populations of young adult worms for imaging were cultured by bleaching unsynchronized gravid adults, and allowing L1 diapause progeny to develop for 2.5 days at 20°C.[31] On the day of imaging, young adults were washed in M9,[32] transferred to the imaging plates (3 worms per well) with a COPAS 500 Flow Pilot,[33] and returned to a 20°C incubator for 3.5 h. Plates were then transferred onto the multi-camera tracker for another 30 min to habituate prior to imaging.[34] For drug experiments, imaging plates were dosed with the compound at the desired concentration 1 day prior to imaging. Worms were then dispensed and tracked as described above, except for the 1 h exposure time where worms were returned to a 20°C incubator for 30 min and then transferred to the tracker for 30 min prior to imaging.[35]

Image acquisition, processing, and feature extraction

Videos were acquired and processed following methods previously described in detail.[36] Briefly, videos were acquired in a room with a nominal temperature of 20°C at 25 frames per second and a resolution of 12.4 μm px^{-1}. Three videos were taken sequentially: a 5-min pre-stimulus video; a 6-min blue light recording with three 10-s blue light pulses starting at 60, 160, and 260 s; and a 5-min post-stimulus recording.

Videos were segmented and tracked with Tierpsy Tracker.[37] After segmentation and skeletonization, a manual threshold was applied to filter skeletonized objects, likely to be non-worms from feature extraction, that did not meet the following criteria: 200–2000 μM length, 20–500 μM width. Tierpsy Tracker's viewer was also used to mark wells with visible contamination, agar damage, or excess liquid as "bad," and exclude these wells from downstream analysis.

Following tracking, we extracted a previously defined set of 3,076 behavioral features for each well in each of the three videos (pre-stimulus, blue light, and post-stimulus).[38] The extraction of behavioral features was performed on a per-track basis and are then averaged across tracks to produce a single feature vector for each well. Statistically significant differences in the pre-stimulus, post-stimulus, and blue-light behavioral feature sets extracted from the loss-of-function mutant compared to the N2 reference strain were calculated with block permutation t-tests (code available on GitHub, see web resources). Permutations were randomly shuffled within, but not between, the independent days of image acquisition in order to control for day-to-day variation in the experiments. The p-values were then corrected for multiple comparisons with the Benjamini-Hochberg procedure[39] to control the false discovery rate at 5%. The code for generating the figures is available on GitHub (see data and code availability).

Pharyngeal pumping assay

Pharyngeal pumps per minute (ppm) of *C. elegans* strains were determined by counting grinder movements over a 15 s period by eye using a stereomicroscope,[40] $n = 120$ worms per strain. Grinder movements of a single worm were counted three times and the results recorded as an average of these values. Statistical differences in ppm between N2 reference strain and *hlb-1(syb4896)* were calculated with block permutation t-tests. The code for generating the figures is available on GitHub (see data and code availability).

Results

Clinical description

All individuals except for individual 7 and individual 9 are offspring of consanguineous parents. Four of the 16 individuals in this cohort deceased during childhood at ages ranging from 3 years and 9 months to 8 years. All individuals shared a core phenotype of global developmental delay/intellectual disability (GDD/ID) and epilepsy. 15 were affected by profound or severe GDD/ID (15/16). They had not acquired speech (15/16) and showed impaired motor development (15/16). Most of them never achieved gross motor milestones such as sitting and walking, except for individual 6-1, who was able to sit independently at the age of 6 years, and individual 7, who could stand and walk. Individual 1 presented with moderate ID, developed expressive language skills, and had a normal motor development. All individuals were affected by epilepsy: most commonly with focal seizures (11/16) including focal to bilateral tonic-clonic (2/16) and one case of impaired awareness seizures (1/16). Furthermore, generalized onset seizures occurred in seven of the individuals (7/16). Epileptic spasms were described in seven (7/16). Other reported seizure types included tonic (3/16) and myoclonic seizures (6/16). The median age of seizure onset was at 2 months with a range from the first day of life up to 4 years. Most individuals were initially affected by daily seizures (12/16). All individuals have

been treated with multiple antiepileptic drugs (AEDs). In the majority, the epilepsy was drug-resistant (13/16) in that they did not achieve sustained seizure freedom of 1 year or, if longer, three times the longest preintervention interseizure interval on therapy with at least two AED schedules.[41] One of them, individual 8, was seizure-free for 5 months at the last assessment since the introduction of valproate. The outcome of this intervention remains undetermined. In individuals 7 and 12, epilepsy was drug-responsive (2/16) as they were sustained seizure-free for 2.5 years and 2 years, respectively. Both received antiepileptic polytherapy including valproate. Individual 2 was seizure-free for 4 to 5 months at the last assessment on her first two combined AED regimens and the responsiveness of the seizures remained undefined. (For further information on antiepileptic treatment, see Table S4.) Electroencephalography (EEG) was performed in 14 individuals (Table S4). EEG findings included focal (3/14) or multifocal (5/14) interictal epileptiform discharges. In two individuals, bilateral paroxysmal discharges were observed (2/14). Hypsarrhythmia was recorded in four individuals (4/14) who were also affected by epileptic spasms and thus met the criteria for West syndrome. In one of these cases, the phenotype progressed to Lennox-Gastaut syndrome later. All but individual 8 were affected by microcephaly (15/16), defined here by an occipitofrontal circumference (OFC) ≤ -2 standard deviations (SD) (range: $<<-3$ SD to -1.78 SD) at last assessment. The majority showed primary (9/16) and/or progressive (11/16) microcephaly. Secondary microcephaly (4/16) developed in individual 1, individual 6-1 (who had a low OFC of -1.94 SD already at birth), individual 7, and individual 12. Individual 8 showed borderline low normal head circumference at the last assessment. Other common neurological findings comprise muscular hypertonia (10/16) up to spastic tetraplegia (6/16), but also muscular hypotonia (5/16), dystonic movements (3/16), and nystagmus (4/16).

Nine individuals were born small for gestational age (birthweight $\leq 10^{th}$ percentile; 9/16). Failure to thrive leading to decreased body weight (≤ -2SD) was seen in eight individuals (8/16), and short stature (height ≤ -2 SD) manifested in seven (7/16). Some of the individuals exhibited feeding difficulties (7/16), and deglutition disorders were described in three of them.

Other repeatedly described symptoms include impaired hearing (4/16), ophthalmologic abnormalities (8/16), undescended testes (3/10), and congenital heart defects (7/16). The latter comprise patent ductus arteriosus (PDA, 6/16), atrial septal defects (ASDs, 3/16), ventricular septal defects (VSDs, 2/16), a dilated left ventricle (1/16), and a coronary fistula (1/16) with mitral regurgitation and cardiomegaly. There were no overarching dysmorphic facial features in the affected individuals. (For an overview of the phenotypic spectrum, see Table 1 and Figure 1A. For further details on the phenotype of each individual, see supplemental notes and Table S4.)

Neuroimaging

Neuroimaging revealed abnormalities of brain morphology in all 14 individuals that underwent MRI, except for individual 1, who had a normal MRI at the age of 18 years. Eleven individuals presented signs of leukoencephalopathy (11/14) (Figure 1C) mainly in a periventricular localization (9/14). Five of the individuals showed paucity of the white matter (5/14). For each of the individuals 3-2 and 4, MRI data from two different time points was available that suggested a progression of the periventricular hyperintensities and loss of white matter, respectively. Seven individuals had abnormalities of the cortex morphology (7/14). Four of them showed disorders of cortical gyration (Figure 1C) including bilateral frontal polymicrogyria (1/14), increased cortical thickness (4/14), and pachygyria (3/14), with one also showing severe periventricular gray matter heterotopia (1/14; Figure 1C: [g]). Cortical atrophy was seen in three (3/14). Ventriculomegaly of variable degree (10/14) was a common finding. Other notable findings included hypoplasia of the corpus callosum (7/14), cerebellar vermian hypoplasia (2/14), and a Blake's pouch cyst (1/14). Head CT scan, performed in eight individuals, revealed bilateral intracranial calcifications (ICCs) in all of them (9/9). Calcifications mostly appeared in a scattered pattern with periventricular localization (9/9) but also the basal ganglia (5/9), centra semiovale (2/9), and internal capsule (2/9) were affected (Figure 1C). Furthermore, CT scan also showed ventriculomegaly in individual 5-2, who did not have MRI.

Fetal phenotype

The fetus (individual 13) showed severe intrauterine growth retardation and microcephaly during pregnancy, and the pregnancy was terminated in the 25th gestational week. Autopsy confirmed length and weight below -2 SD and an occipitofrontal circumference below -4 SD. An X-ray babygram showed ICCs (Figure 1B), and the histopathological examination of the brain revealed predominant macrocalcification and rare necrotic foci in the process of calcification in the germinative and periventricular areas around the 3rd ventricle and occipital horns, as well as cerebral edema with spongiosis and glial response. As an additional finding, autopsy revealed a bicornuate uterus. The parents of the fetus are healthy individuals.

Genetic results

ES and GS revealed homozygous LoF variants in *PPFIBP1* (GenBank: NM_003622.4) in all affected individuals. In the affected individuals of the families 2-12, we detected eight different homozygous protein-truncating variants. These comprise five nonsense variants and three frameshift variants. Three of the variants were recurrent as each was identified in two unrelated families. (All variants are displayed in Table 1.) Because all of these variants lead to premature termination codons > 50 nucleotides upstream of the last exon-exon splice junction considering the transcripts with the highest expression overall and

Table 1. Clinical and genetic details of all affected individuals with causative variants in *PPFIBP1*

Ind.	Age[a] (sex)	Variant (GenBank: NM_003622.4)	Development	Seizure types (age of onset)	MRI (age)	ICCs[b]	Neurological findings	Microcephaly	Growth	CHD	Ophthalmologic features
**Ind. 1**	19 years (M)	c.1146+1G>A (p.?), homozygous	moderate ID, delayed speech, normal motor development	focal impaired awareness (4 years)	normal (18 years)	not done	none	yes	SGA	no	normal
**Ind. 2**	6 years[c] (6 years) (F)	c.2654del (p.Tyr885Leufs*4), homozygous	profound DD, no speech, unable to sit	focal, generalized tonic clonic (2 months)	paucity of the WM, VM, hypoplastic CC, Blakes's pouch cyst (5 years)	yes	spastic tetraplegia, nystagmus	yes	short stature, low weight	no	bilateral papillary pallor, no eye contact
**Ind. 3-1**	11 years (M)	c.1368_1369del (p.Glu456Aspfs*3), homozygous	profound DD, no speech, unable to sit	epileptic spasms, focal, tonic clonic, tonic (7 months)	periventricular leukomalacia, metopic synostosis	yes	spastic tetraplegia	yes	SGA, low weight	yes	poor fixation
**Ind. 3-2**	7 years (M)	c.1368_1369del (p.Glu456Aspfs*3), homozygous	profound DD, no speech, unable to sit	epileptic spasms, LGS (2 months)	moderate hyperintensity of periventricular white matter, mild VM (2 years)	yes	spastic tetraplegia	yes	SGA, low weight	yes	normal
**Ind. 3-3**	5 years (M)	c.1368_1369del (p.Glu456Aspfs*3), homozygous	profound DD, no speech, unable to sit	epileptic spasms, focal, multifocal (1 day)	VM, abnormal signal intensity of the WM, bilateral temporal and left occipital pachygyria (3 days)	yes	spastic tetraplegia	yes	SGA, low weight	yes	normal
**Ind. 4**	11 months (M)	c.1368_1369del (p.Glu456Aspfs*3), homozygous	profound DD no speech, unable to sit	epileptic spasms, focal, generalized tonic, status epilepticus (5 months)	VM, paucity of the WM, bilateral parietal and occipital pachygyria (5 months)	yes	spastic diplegia, hyperreflexia	yes	SGA, short stature	yes	haemorrhagic retinitis, chronic retinal detachment, right eye exotropia w/ slow pupillary reaction
**Ind. 5-1**	8 years[c] (8 years) (F)	c.2413C>T (p.Arg805*), homozygous	profound DD, no speech, unable to sit	focal, myoclonic (2 months)	not done	yes	spastic tetraplegia	yes	SGA, short stature, low weight	no	N/A
**Ind. 5-2**	2 years[c] (4 years) (F)	c.2413C>T (p.Arg805*), homozygous	profound DD, no speech, unable to sit	focal, myoclonic, tonic (1 months)	not done, VM on CT	yes	hypertonia of the limbs, dystonia	yes	short stature	no	normal
**Ind. 6-1**	6 years (M)	c.1468C>T (p.Gln490*), homozygous	profound DD, no speech, sat independently at 6 years	generalized tonic clonic, myoclonic (4 months)	VM, cortical atrophy, demyelination of periventricular WM, thin CC, cerebellar vermian hypoplasia	not done	hypertonia of the limbs	yes	short stature, low weight	no	optic atrophy, followed light
**Ind. 6-2**	2 years (M)	c.1468C>T (p.Gln490*), homozygous	profound DD, no speech, no head support	generalized tonic clonic, myoclonic, excessive smacking movements (2 months)	asymmetrical VM, cortical atrophy, demyelination of periventricular WM, thin CC, cerebellar vermian hypoplasia	yes	spasticity, rigidity, dystonic movement	yes	short stature, low weight	yes	optic atrophy, couldn't follow light

(*Continued on next page*)

Table 1. ***Continued***

Ind.	Age[a] (sex)	Variant (GenBank: NM_003622.4)	Development	Seizure types (age of onset)	MRI (age)	ICCs[b]	Neurological findings	Microcephaly	Growth	CHD	Ophthalmologic features
**Ind. 7**	4 years (F)	c.403C>T (p.Arg135*), homozygous	severe DD, no speech, motor delay but can stand and walk	epileptic spasms, focal with apnoea, myoclonic (4 months)	normal at 4 months; thin CC, periventricular dysmyelination, possibly reduction of the WM at 1.5 years	not done	hypotonia	yes	normal	no	normal
**Ind. 8**	N/A (F)	c.1417_1427del (p.Ala473Lysfs*20), homozygous	profound DD, no speech, unable to sit	generalized tonic clonic (6 months)	bilateral parietal pachygyria, periventricular heterotopia, VM, hyperintensity and paucity of the WM	N/A	hypotonia, nystagmus	no, but low OFC	SGA	yes	normal, but poor fixation
**Ind. 9**	2 years 6 months[c] (3 years 9 months) (M)	c.1300C>T (p.Gln434*), homozygous	severe DD, no speech, can sit but not walk	epileptic spasms and gaze (2 months)	abnormal	N/A	spastic tetraplegia, no sphincter control	yes	SGA	no	blindness
**Ind. 10**	1 years 2 month (M)	c.2629C>T (p.Arg877*), homozygous	severe DD, no speech yet, motor delay	focal myoclonic, epileptic spasms (1 week)	abnormal myelination of the periventricular WM and at corona radiata and centrum semiovale, hypoplastic CC, mild VM	N/A	hypotonia, nystagmus	yes	N/A	no	right ptosis, left iris coloboma, diffuse chorioretinal degeneration
**Ind. 11**	5 months (M)	c.1468C>T (p.Gln490*), homozygous	severe DD, no speech yet, no head support	focal, myoclonic (2 weeks)	cortical atrophy, deep Sylvian fissures, mild VM, prominent basal ganglia, hypoplastic CC, retrocerebellar and bitemporal arachnoid cysts	yes	hypotonia, dystonia, brisk reflexes, nystagmus	yes	SGA	yes	optic atrophy
**Ind. 12**	5 years 11 months (F)	c.2654del (p.Tyr885Leufs*4), homozygous	profound DD, no speech, unable to sit	focal, generalized (6 months)	VM, leukoencephalopathy, paucity of the WM, suspected periventricular microcalcifications, frontal polymicrogyria, temporoparietal thickening of the cortex (5 years)	not done	hypotonia, dyskinesia, stereotypic movements	yes	short stature, low weight	N/A	abnormalities of VEPs
**Fetus (ind. 13)**	25th GW	c.2177G>T (p.Gly726Val), homozygous	developmental age estimated around 22nd GW	–	–	yes[d]	–	yes	IUGR	–	–

Abbreviations: CC, corpus callosum; CHD, congenital heart defect; DD, developmental delay; F, female; IUGR, intrauterine growth restriction; GDD, global developmental delay; GW, gestational week; ICCs, intracranial calcifications; ID, intellectual disability; LGS, Lennox-Gastaut syndrome; M, male; N/A, not available; OFC, occipitofrontal circumference; SGA, small for gestational age; VEPs, visually evoked potentials; VM, ventriculomegaly; WM, white matter.
Further clinical details are provided in Table S4.
[a]Age at last assessment.
[b]On CT scan.
[c]Deceased (age at death).
[d]ICCs seen on X-ray babygram.

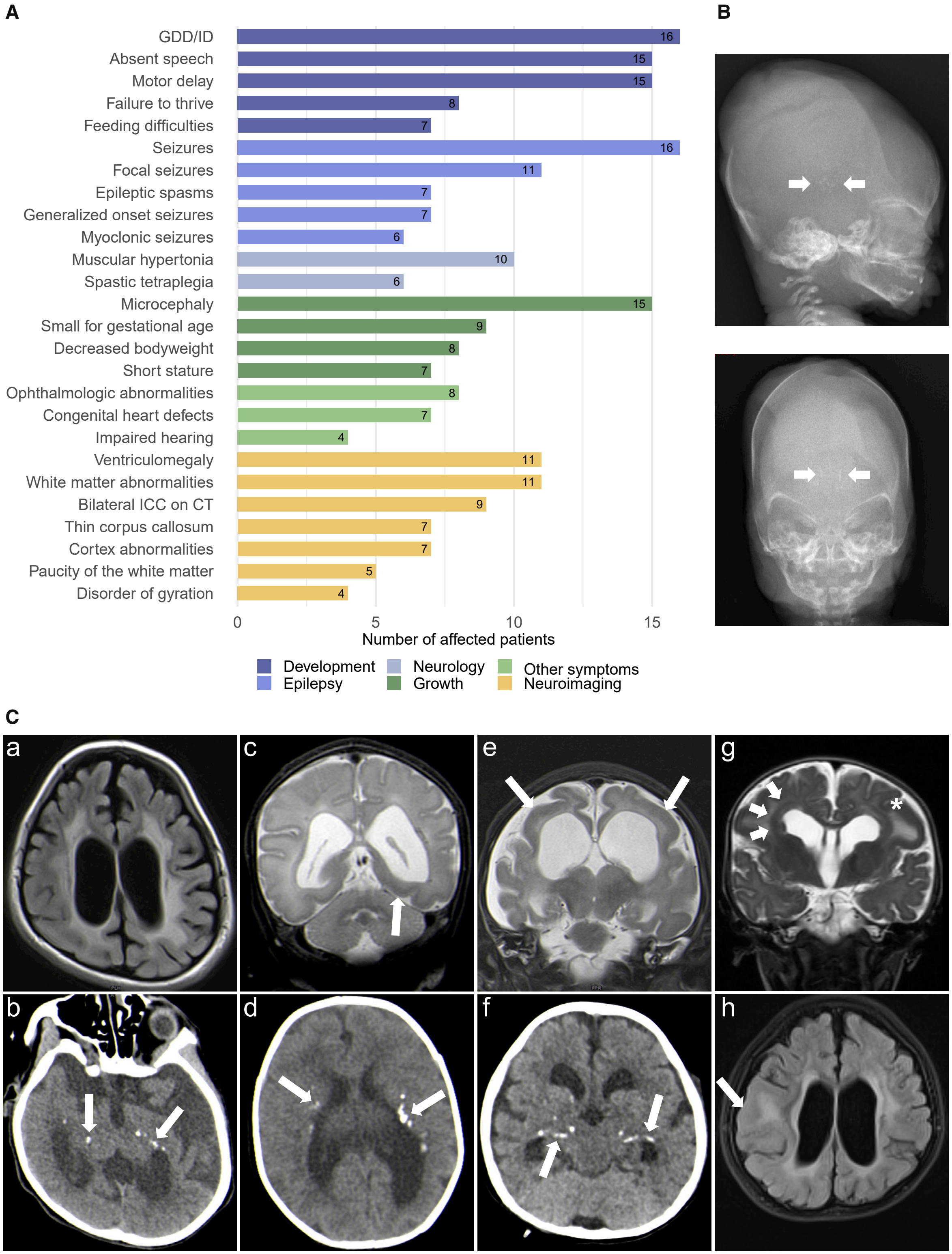

Figure 1. Prevalence of clinical findings, neuroimaging features, and X-ray of the fetus
(A) Prevalence of phenotypic features in the cohort grouped by clinical categories.
(B) Fetus, 25th gestational week: X-ray babygram postmortem showing macroscopic intracranial calcifications (arrows).
(C) Exemplary MRI and CT images. a) Individual 2, MRI, age 5 years, T2-FLAIR axial: pronounced leukoencephalopathy with hyperintensities of the white matter, paucity of the white matter, consecutive ventriculomegaly. b) Individual 2, CT, age 5 years: bilateral symmetrical calcifications periventricular and in the basal ganglia (arrows). c) Individual 3-3, MRI, age 3 days, T2-TSE coronal: moderate ventriculomegaly with accentuation of the occipital horn and pachygyria with thickening of the occipitotemporal cortex (arrow). d) Individual 3-3, CT, age 3 days, bilateral calcifications periventricular and in the deep white matter (arrows). e) Individual 4, MRI, age 3 months, T2-TSE coronal: severe paucity of the white matter and consecutive ventriculomegaly, bilateral pachygyria, and thickening

(legend continued on next page)

in brain tissues in specific (GenBank: NM_003622 and GenBank: NM_001198915.2),[15,16] they are predicted to undergo nonsense-mediated mRNA decay (NMD).[42] For two of the variants described above, limitations to the prediction of NMD have to be considered. For the variant c.403C>T (p.Arg135*), NMD can only be predicted (Figure S2) with respect to transcript GenBank: NM_003622.4, as the variant lies in the 5′ untranslated region (UTR) of the transcript Genbank: NM_001198915.2. The variant c.1300C>T (p.Gln434*) in exon 15/30 (GenBank: NM_003622.4) is predicted by SpliceAI[19] to cause a loss of the acceptor- and donor-splice sites of exon 15 with Δ-scores of 0.39 and 0.3, respectively, which means the variant could affect splicing at these positions. A loss of these splice sites would lead to an in-frame deletion of exon 15, which would potentially be less disruptive on protein function than NMD due to a nonsense variant. All LoF variants mentioned above can be classified as pathogenic according to the guidelines of the ACMG[14] except for the variant c.1300C>T (p.Gln434*), which can only be classified as of unknown significance. Nonetheless, this variant is deemed causative due to a high phenotypic overlap with the rest of the cohort (Table S1).

In family 1, a homozygous splice-site variant, c.1146+1G>A (p.?), affecting the consensus 5′-splice site of exon 13 was identified. Multiple *in silico* tools consistently predict a loss of the splice site (Table S2). This could lead to out-of-frame exon skipping or to intron retention.[43,44] Thus, the mRNA resulting from this allele is likely to include a premature termination codon, thus resulting in NMD (Figure S1).

Furthermore, in a fetus, a homozygous missense variant, c.2177G>T (p.Gly726Val) was identified. The missense variant lies in the second SAM domain (Figure 2A) and affects a highly conserved amino acid considering nine species up to the opossum. Multiple *in silico* tools consistently predicted a damaging effect of the variant (Table S3). Structural analysis showed that Gly726 is located in a tight turn of the second SAM domain of *PPFIPB1* (Figure 2B). At this position, a valine can only be accommodated in a strained backbone conformation resulting in domain destabilization. In addition, the longer valine sidechain causes steric problems with Asn803 located in the third SAM domain (Figure 1B), which are predicted to disrupt the domain interface.

All of the identified variants are very rare in the general population, represented by gnomAD.[15] The variants detected in the families 1, 2, 3, 4, 6, 8, 9, 10, 11, and 12 and in the fetus are absent from gnomAD. Five alleles are reported for the variant identified in family 5 (MAF of 0.0000199) and seven alleles are reported for the variant identified in family 7 (MAF of 0.00002828), all in heterozygous state in each case. The parents were confirmed as heterozygous carriers in the families 1, 2, 4, 5, 6, 7, 8, 10, 11, 12, and 13 by Sanger sequencing and/or trio ES/GS.

Modeling loss of *PPFIBP1* in *C. elegans*

Worm models are useful for modeling the underlying mechanistic causes of genetic disorders. Automated quantitative phenotyping of the disease model mutant *hlb-1(syb4896)* was used to identify differences compared to the wild-type strain N2 across a range of behavioral dimensions.[38]

Loss of *hlb-1* did not result in developmental delay or a growth defect in *C. elegans*, however the *hlb-1(syb4896)* mutant showed a significant increase in body curvature (Figure 3A). In existing *C. elegans* models of epilepsy "head bobbing" is a phenotype associated with convulsions and the onset of seizures.[45] We saw no statistically significant difference in the head movement of *hlb-1(syb4896)* compared to N2 during baseline (pre-stimulus) tracking (Figure 3B). However, upon stimulation with pulses of blue light, a significant increase in the acceleration of the head tip (indicative of increased head movement) was observed for mutant strains (Figure 3C), highlighting some overlap in the behavioral phenotype of *hlb-1(syb4896)* and other pre-existing worm models of epilepsy. Thus, this finding indirectly suggests some elements of a mild epileptic phenotype may be present in *hlb-1(syb4896)*.

There is little difference in the baseline locomotion of *hlb-1(syb4896)* and N2 (Figure 3E). However, *hlb-1(syb4896)* displays a short-lived photophobic escape response when pulsed with blue light, as demonstrated by the LoF mutant returning to a paused state faster upon the cessation of the aversive stimulus (Figures 3F–3G). We also note that there is an attenuated change in posture of *hlb-1(syb4896)* during blue light tracking (Figure 3H).

A previous study into the function of *hlb-1* in *C. elegans* identified a defect in pharyngeal pumping rate,[10] which we also confirm for *hlb-1(syb4896)* (Figure 3D), and enlarged pre- and post-synaptic sites. Given the role of aberrant synaptic transmission events in the onset of epileptic seizures and the hypothesis that liprin-β1 acts as a core scaffold to mediate protein assembly in the presynaptic zone,[3] we investigated whether our quantitative phenotyping approach could detect a defect in the synaptic transmission apparatus of *hlb-1(syb4896)*.

Aldicarb is an acetylcholinesterase inhibitor that induces paralysis of the body-wall muscles in *C. elegans* as a result of an accumulation of acetylcholine (ACh) and the subsequent overstimulation of acetylcholine receptors. Increased resistance to aldicarb occurs if mutations give rise to defects in presynaptic function, as ACh accumulates in the neuromuscular junction at a slower rate.[46] Indeed, *hlb-1(syb4896)* showed a significant dose-dependent decrease in the fraction of paused worms that were

of the parietal cortex (arrows). f) Individual 4, CT, age 3 months: bilateral symmetrical calcifications periventricular and in the basal ganglia (arrows). g) Individual 8, MRI, age 6 months, T2-TSE coronal: ventriculomegaly, pachygyria with thickening of the cortex (asterisk), and periventricular gray matter heterotopia (arrow). h) Individual 12, MRI, age 5 years, T2-FLAIR axial: ventriculomegaly, leukoencephalopathy, paucity of the white matter, and thickening of the cortex (arrow).

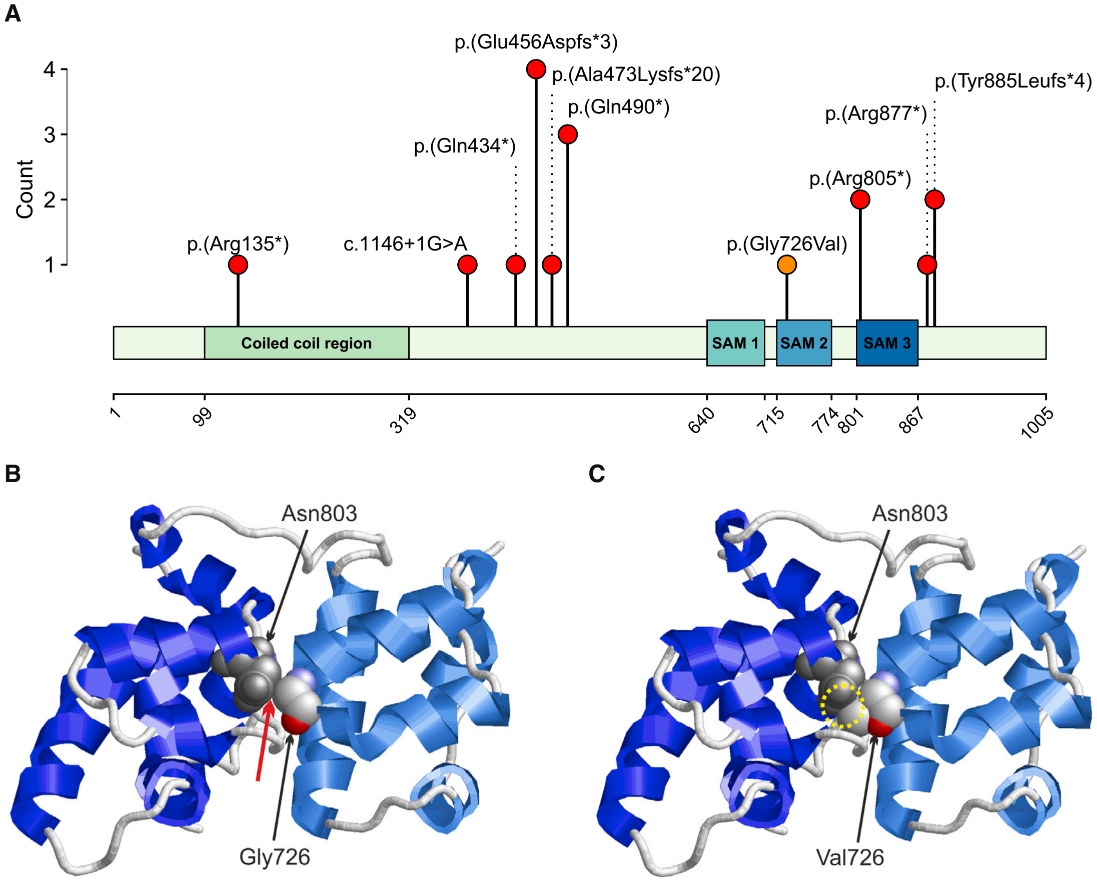

Figure 2. Variant locations on protein level and structure of liprin-β1 illustrating the effect of the p.Gly726Val exchange
(A) Location of the variants on protein level aligned to the liprin-β1 isoform 1 (GenBank: NP_003613.4 [GenBank: NM_003622.4]) and count of each variant in the cohort. Truncating and splice-site variants are indicated by red dots, the missense variant is indicated by an orange dot. Abbreviation: SAM, sterile alpha motif.
(B) Structure of SAM2 and SAM3 domains that pair in the wild-type protein. Gly726 is located in a tight turn and makes interactions with Asn803 of the adjacent SAM domain. The contacts are formed between Gly732(Cα) and Asn803(Cβ) (distance = 3.5 Å) and between Gly732(Cα) and Asn803(Cγ) (distance = 3.6 Å). The site of the contacts is marked by a red arrow; Asn803 is shown in grey and Gly726 is colored by atom type. The topology of the protein backbone is schematically depicted with helices in light blue (SAM2 domain) and dark blue (SAM3 domain).
(C) In the Gly726Val variant, a severe steric overlap (yellow circle) between the sidechains of Val726 and Asn803 is observed, which will disrupt the domain interface thereby altering the topology of the SAM domain region.

exposed to 1–10 μM aldicarb for 1 h compared to N2 (Figure 4A), demonstrating increased aldicarb resistance. Levamisole is a paralysis-inducing ACh receptor agonist. Resistance to levamisole has been shown to persist in worms if mutations affect the postsynaptic site, whereas sensitivity to levamisole persists if mutations only affect the presynaptic site.[47] In contrast to previous *hlb-1* studies,[10] we do not observe any resistance to levamisole in *hlb-1(syb4896)* worms. If anything, there is an increased sensitivity observed at 10 μM levamisole for 4 h (Figure 4B).

These findings provide evidence that a defect arises in the presynaptic, but not postsynaptic, apparatus of *C. elegans* as a result of *hlb-1* LoF. Coupled with existing evidence that liprins are involved in the assembly of presynaptic active zones across species,[3,7] this points towards a conserved biological role of *hlb-1* and its orthologs in regulating the formation of NMJs and supports presynaptic defects as a cause of the pathologies arising from mutations in *PPFIBP1*.

Discussion

Here we describe 16 individuals from 12 unrelated families with a core phenotype of moderate to profound developmental delay, progressive microcephaly, epilepsy, and periventricular calcifications. In all 16 individuals, ES and GS revealed rare homozygous LoF variants in *PPFIBP1*. In addition, we describe a fetus with severe growth restriction, microcephaly, and intracranial calcifications with a homozygous missense variant that is *in silico* and structurally predicted to be disrupting.

Consistent with the proposed autosomal recessive inheritance, LoF variants in *PPFIBP1* in the general population are not common with an observed/expected ratio (o/e) of 0.57 (90% confidence interval = 0.43–0.75). In

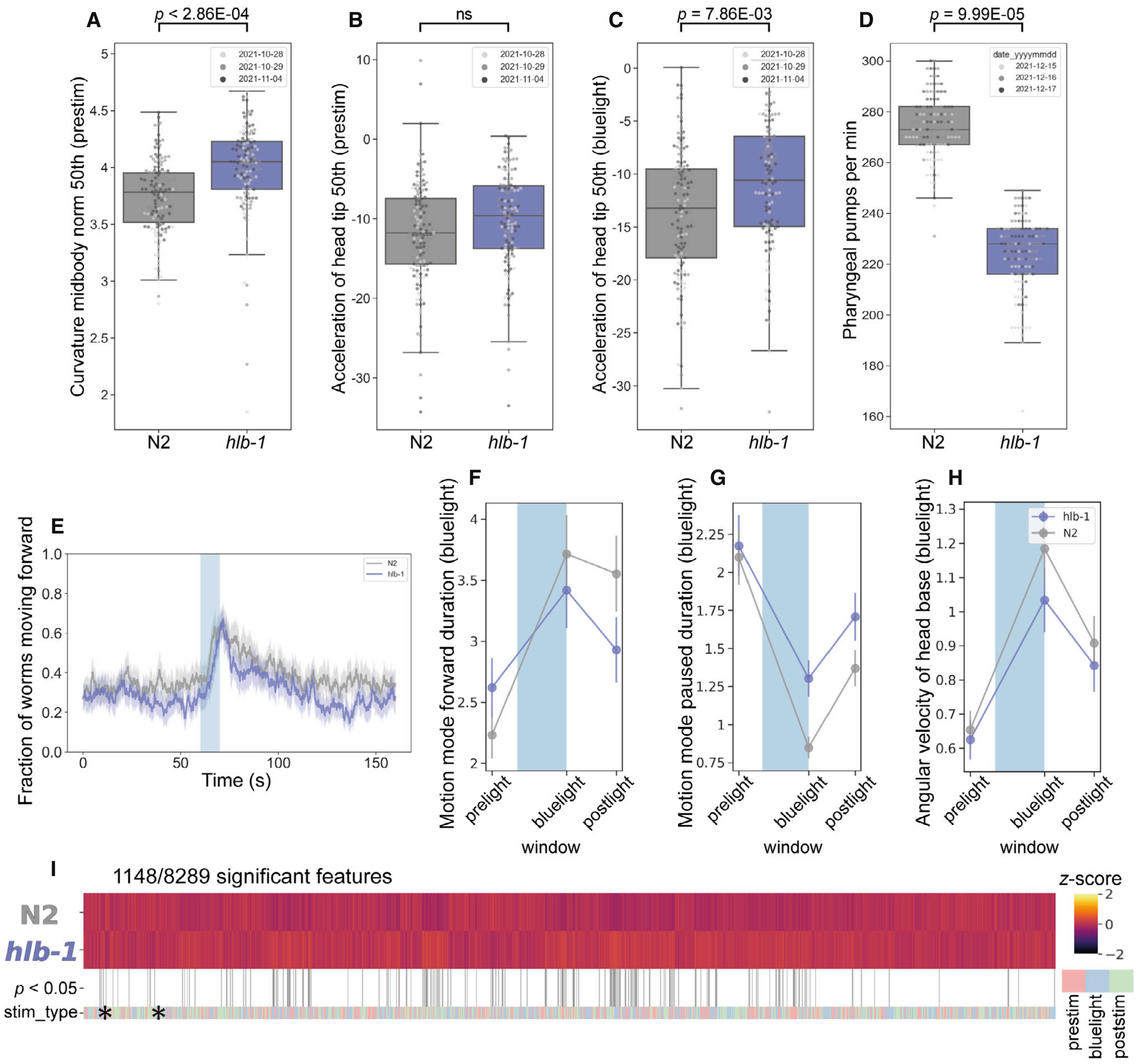

Figure 3. Behavioral phenotype of *Caenorhabditis elegans PPFIBP1* ortholog, *hlb-1(syb4896)*

(A–C) Example behavioral and postural features altered in the loss-of-function *hlb-1(syb4896)* [*C. elegans* ortholog of *PPFIBP1*] mutant strain under baseline (pre-stimulus) imaging conditions. Individual points marked on the box plots are average values from multiple worms in a single well. The different point colors indicate data from independent experimental days. The selected features were compared to the N2 reference strain with block permutation t-tests, and p values are shown above the respective plots.

(D) Pharyngeal pumps per minute of *hlb-1(syb4896)* and N2 reference strain.

(E) Overall fraction of worms moving forward 60 s prior to and 80 s following stimulation with a 10 s blue light pulse (blue shading). Colored lines represent averages of the detected fraction of paused worms across all biological replicates and shaded areas represent the 95% confidence intervals.

(F–H) Average changes in the total fraction of worms moving forward or paused prior to, during, and following stimulation with blue light (F and G), average change in an example postural feature in response to blue light (H). Feature values were calculated as averages of 10 s window summaries centered around 5 s before, 10 s after, and 20 s after the beginning of a 10 s blue light pulse (blue shading).

(I) Heatmap of the entire set of 8,289 behavioral features extracted by Tierpsy for *hlb-1(syb4896)* and N2. The stim_type barcode denotes when during image acquisition the feature was extracted: pre-stimulation (pink), blue light stimulation (blue), and post-stimulation (green). Asterisks indicate the selected features present in the box plots above (A–C) and the color map (right) represents the normalized *Z* score of the features.

addition, there were no homozygous LoF variants observed in gnomAD. Because all described variants are ultra-rare (MAF < 0.01%), it is highly unlikely to assemble a cohort with this level of phenotypical overlap and homozygous LoF variants in *PPFIBP1* by coincidence, which further strengthens disease causality.

The 15 individuals harboring homozygous frameshift or nonsense variants exhibit a consistent phenotype in terms

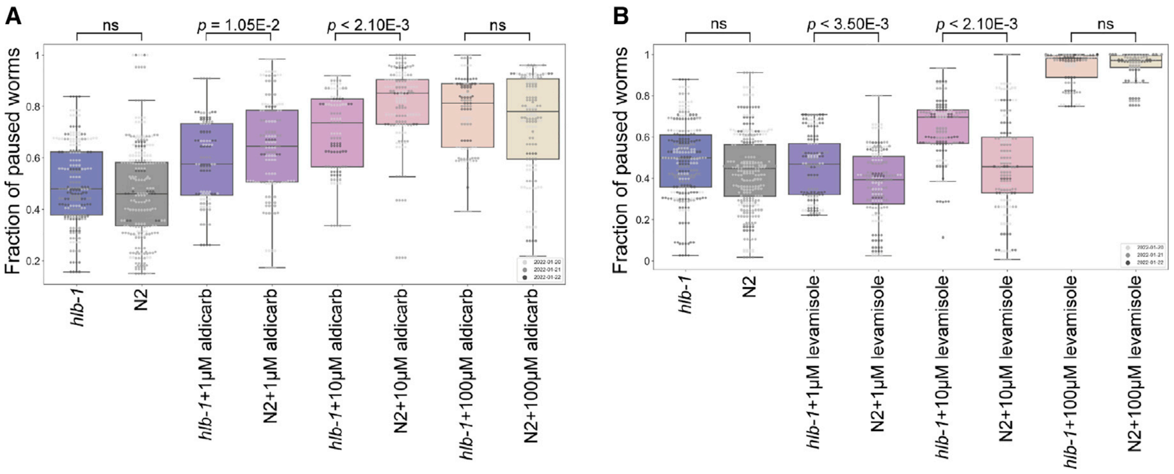

Figure 4. Fraction of paused worms in response to treatment with aldicarb or levamisole
(A and B) Overall fraction of paused worms after (A) 1 h exposure to aldicarb and (B) 4 h exposure to levamisole at the concentrations denoted under the boxplots. N2 (grey) and *hlb-1* (blue) are solvent only controls (DMSO and ddH2O for aldicarb and levamisole, respectively). Individual points marked on the box plots are averaged values from multiple worms in a single well. The different point colors indicate data from independent experimental days. The fraction of paused *hlb-1(syb4896)* worms was compared to the fraction of paused N2 worms at each concentration with block permutation t-tests, with $p > 0.05$ considered not significant (ns), $n = 30$ wells for each compound and concentration tested.

of the severity of the developmental delay, epilepsy, and frequently found neuroimaging features. Only individual 7 presented a milder disease course compared to the other individuals with truncating variants, as she had secondary microcephaly, was able to stand and walk, albeit showing impaired motor development, and showed less prominent neuroimaging features. The nonsense variant c.403C>T (p.Arg135*) found in individual 7 lies in the 6th exon thus being the most upstream variant in this cohort. It is predicted to cause NMD considering the transcript GenBank: NM_003622.4, which shows the highest overall expression and thereby most likely has the highest biological relevance. Nevertheless, it cannot be ruled out that a shorter transcript like GenBank: NM_001198915.2 with its start codon lying 57 base pairs downstream of this variant could compensate for the loss of the main transcript to some extent. GenBank: NM_001198915.2 has the second highest mean expression across all tissues and particularly shows expression levels that are comparable to those of GenBank: NM_003622.4 in some areas of the brain.[16]

Individual 1 with the homozygous splice-site variant has a milder phenotype compared to the other individuals with nonsense or frameshift variants, although he shares the core clinical signs. This could be due to an incomplete splice defect, either leading to the expression of a fraction of normal protein or to an altered protein not completely impaired in function or stability. Canonical splice site variants as observed in individual 1 can have a variety of effects on pre-mRNA splicing such as exon skipping, which is the most common mechanism in variants disrupting consensus 5′-donor splice sites[43] and would result in a frameshift in this case. However, a loss of the splice site could also result in intron retention with a premature termination-codon or enable the activation of a cryptic splice site with subsequent inclusion of an intron fragment or the removal of an exon fragment either inframe or out of frame. Both of the latter possibilities can lead to a variety of aberrant transcripts.

The pathogenicity of the missense variant identified in the fetus is not as clear as that of the LoF variants. However, the striking similarity of the intracranial calcifications, the growth restriction, and the severe microcephaly represent a significant phenotypic overlap with the rest of the cohort, suggesting this variant to be causative. Potential pathogenicity of the variant is further supported by its absence from the general population, by multiple *in silico* predictions and its expected effects on the SAM domains from structural analysis. SAM domains are a family of protein interaction modules present in a wide variety of proteins.[48] The Gly726Val exchange is located in the second SAM domain of PPFIBP1 destabilizing both the second SAM domain and the interaction between the second and third SAM domain. Therefore, this variant is expected to severely disturb the topology of the SAM domain region and its function in protein-protein interactions. Given the hypothesis that liprin-β1 acts as a core scaffold to mediate protein assembly in the presynaptic zone,[3] the ability to precisely interact with other proteins would appear to be critical for protein function.

ICCs located in the periventricular area but also affecting the basal ganglia and the internal capsule appear to be a highly characteristic sign in this cohort. Pathologic ICCs have heterogeneous etiologies such as neoplastic, infectious, vascular, metabolic, and genetic conditions.[49] Congenital infections with pathogens of the TORCH-spectrum, and congenital cytomegalovirus (CMV) infections in particular, account for a significant amount of congenital and pediatric ICCs that are associated with brain

malformations and impaired neurodevelopment.[50] However, genetic disorders such as interferonopathies represent important differential diagnoses for congenital ICCs and some conditions significantly overlap with the symptomatic spectrum of congenital TORCH-infections.[51–53] It is assumed that the genetic etiologies of unsolved ICCs have not been fully discovered yet.[50,54] Individual 4 was admitted to the neonatal intensive care unit for 1 month after birth, as his clinical presentation was indicative of a congenital CMV infection (see supplemental notes for further details). However, an active CMV infection could not be confirmed in standard laboratory diagnostics. In both affected siblings of family 5 and in individual 6-2, a screening for infections of the TORCH spectrum was performed with negative results and also the fetus was tested negative for CMV.

To date, no alterations in any of the human liprin genes have been associated with human disease. The biological function of liprin-β1 and its molecular mechanisms are still largely unstudied. However, recent studies point towards a role in neurodevelopment that echo the findings of a neurodevelopmental disorder in the cohort described here. Liprin- β1 has been identified as a binding partner of liprin-α proteins. The role of liprin-α proteins or their orthologues in synapse formation and synaptic transmission has been demonstrated in previous animal model studies.[7,55–57] Liprin-α proteins function as major scaffold proteins at the presynaptic active zone and at the postsynaptic density and also play a role in intracellular transport, cell motility, and protein assembly.[3,5,6,58–60] Wei et al. found that liprin-α2 forms a ternary complex simultaneously binding liprin-β1 and CASK, another presynaptic scaffold protein, supporting the hypothesis that liprin-β1 could act as a core scaffold and mediate large protein assemblies in the presynaptic active zone.[3] Interestingly, pathogenic variants in *CASK* (MIM: 300172) are associated with X-linked neurodevelopmental disorders.[61] Pathogenic variants in *CASK* cause X-linked neurodevelopmental disorders with varying phenotypes depending on variant type and inheritance. In particular, heterozygous and hemizygous LoF variants in *CASK* lead to microcephaly with pontine and cerebellar hypoplasia (MICPCH [MIM: 300749]). The phenotypic spectrum comprises moderate to profound ID, progressive microcephaly, impaired hearing, ophthalmologic anomalies, muscular hypo- or hypertonia and spasticity, as well as seizures and partly epileptic encephalopathy in males.[61] Because the phenotype is overlapping with the clinical signs found in this cohort, it seems possible that *PPFIBP1* and *CASK* are involved in similar biological functions such as protein assembly in the presynaptic active zone. Supporting the potential role of liprin-β1 in synapse formation and neurodevelopment, we have shown here that a *C. elegans PPFIBP1/hlb-1* knockout model shows defects in spontaneous and light-induced behavior. The observed sensitivity of the worm model to the acetylcholinesterase inhibitor aldicarb supports a presynaptic defect as at least a partial cause of the observed behavioral phenotypes. This is broadly consistent with previous work showing that null-allele mutants of the *drosophila* orthologs liprin-β and liprin-α independently cause abnormal axon outgrowth, target layer recognition, and synapse formation of R7 photoreceptors as well as reduced larval NMJ size in *Drosophila melanogaster.* Interestingly, distinct effects on axon outgrowth between single liprin-β and liprin-α mutants and an additive effect in double mutants were observed, indicating independent functions of both proteins.[7]

In summary, we establish bi-allelic loss-of-function variants in *PPFIBP1* as a cause for an autosomal recessive severe neurodevelopmental disorder with early-onset epilepsy, microcephaly, and periventricular calcifications.

Data and code availability

All identified variants in *PPFIBP1* have been uploaded to ClinVar https://www.ncbi.nlm.nih.gov/clinvar/submitters/506086/ with the following accession numbers: c.1146+1G>A (p.?) in individual 1 (ClinVar: VCV001679175); c.2654del (p.Tyr885Leufs*4) in individuals 2 and 12 (ClinVar: VCV001679176); c.1368_1369del (p.Glu456Aspfs*3) in individuals 3-1, 3-2, 3-3, and 4 (ClinVar: VCV001679177); c.2413C>T (p.Arg805*) in individuals 5-1 and 5-2 (ClinVar: VCV001679178); c.1468C>T (p.Gln490*) in individuals 6-1, 6-2, and 11 (ClinVar: VCV001679179); c.403C>T (p.Arg135*) in individual 7 (ClinVar: VCV001679180); c.1417_1427del (p.Ala473-Lysfs*20) in individual 8 (ClinVar: VCV001679181); c.1300C>T (p.Gln434*) in individual 9 (ClinVar: VCV001679182); c.2629C>T (p.Arg877*) in individual 10 (ClinVar: VCV001679183); c.2177G>T (p.Gly726Val) in the fetus (ClinVar: VCV001679120); c.2158+2T>C (p.?) in individual 14 (see supplemental information). The code used for tracking and extracting *C. elegans* behavioral features is available at https://github.com/Tierpsy and code for performing statistical analysis and generating figures is available at https://github.com/Tom-OBrien/Phenotyping-hlb1-disease-model-mutant. The associated *C. elegans* datasets are available at https://doi.org/10.5281/zenodo.6338403. The Morbid Genes Panel is available here https://morbidgenes.org/ and here https://zenodo.org/record/6136995#.YiYvI-jMKUk.

Supplemental information

Supplemental information can be found online at https://doi.org/10.1016/j.ajhg.2022.06.008.

Acknowledgments

We thank all families that participated in this study. This project has received funding from the European Research Council under the European External Action Service Horizon 2020 Research and Innovation Program (grant agreement no. 714853) and was supported by the Medical Research Council through grant MC-A658-5TY30. H.T. was supported by the European External Action Service Seventh Framework Programme for research, technological development, and demonstration under grant agreement no. 608473.

Declaration of interests

The authors declare no competing interests.

Received: April 1, 2022
Accepted: June 13, 2022
Published: July 12, 2022

Web resources

BLAST, https://blast.ncbi.nlm.nih.gov/Blast.cgi
Ensembl, https://www.ensembl.org/index.html
GeneMatcher, https://genematcher.org/
GenBank, https://www.ncbi.nlm.nih.gov/genbank/
gnomAD, https://gnomad.broadinstitute.org/
GTEx, https://gtexportal.org/home/
MorbidGenes, https://morbidgenes.org/
OMIM, https://www.omim.org/
PDB, https://www.rcsb.org/
SpliceAI, https://spliceailookup.broadinstitute.org/
SwissModel, https://swissmodel.expasy.org/

References

1. Serra-Pagès, C., Medley, Q.G., Tang, M., Hart, A., and Streuli, M. (1998). Liprins, a family of LAR transmembrane protein-tyrosine phosphatase-interacting proteins. J. Biol. Chem. *273*, 15611–15620. https://doi.org/10.1074/jbc.273.25.15611.
2. Zürner, M., and Schoch, S. (2009). The mouse and human Liprin-α family of scaffolding proteins: genomic organization, expression profiling and regulation by alternative splicing. Genomics *93*, 243–253. https://doi.org/10.1016/j.ygeno.2008.10.007.
3. Wei, Z., Zheng, S., Spangler, S.A., Yu, C., Hoogenraad, C.C., and Zhang, M. (2011). Liprin-mediated large signaling complex organization revealed by the liprin-α/CASK and liprin-α/liprin-β complex structures. Mol. Cell *43*, 586–598. https://doi.org/10.1016/j.molcel.2011.07.021.
4. Xie, X., Luo, L., Liang, M., Zhang, W., Zhang, T., Yu, C., and Wei, Z. (2020). Structural basis of liprin-α-promoted LAR-RPTP clustering for modulation of phosphatase activity. Nat. Commun. *11*, 169. https://doi.org/10.1038/s41467-019-13949-x.
5. Spangler, S.A., and Hoogenraad, C.C. (2007). Liprin-α proteins: scaffold molecules for synapse maturation. Biochem. Soc. Trans. *35*, 1278–1282. https://doi.org/10.1042/bst0351278.
6. Xie, X., Liang, M., Yu, C., and Wei, Z. (2021). Liprin-α-Mediated assemblies and their roles in synapse formation. Front. Cell Dev. Biol. *9*, 653381. https://doi.org/10.3389/fcell.2021.653381.
7. Astigarraga, S., Hofmeyer, K., Farajian, R., and Treisman, J.E. (2010). Three Drosophila liprins interact to control synapse formation. J. Neurosci. *30*, 15358–15368. https://doi.org/10.1523/jneurosci.1862-10.2010.
8. Becker, M., Mastropasqua, F., Reising, J.P., Maier, S., Ho, M.-L., Rabkina, I., Li, D., Neufeld, J., Ballenberger, L., Myers, L., et al. (2020). Presynaptic dysfunction in CASK-related neurodevelopmental disorders. Transl. Psychiatry *10*, 312. https://doi.org/10.1038/s41398-020-00994-0.
9. Hsueh, Y.-P. (2009). Calcium/calmodulin-dependent serine protein kinase and mental retardation. Ann. Neurol. *66*, 438–443. https://doi.org/10.1002/ana.21755.
10. Wang, D.-Y., and Wang, Y. (2009). HLB-1 functions as a new regulator for the organization and function of neuromuscular junctions in nematode Caenorhabditis elegans. Neurosci. Bull. *25*, 75–86. https://doi.org/10.1007/s12264-009-0119-9.
11. Shaheen, R., Maddirevula, S., Ewida, N., Alsahli, S., Abdel-Salam, G.M.H., Zaki, M.S., Tala, S.A., Alhashem, A., Softah, A., Al-Owain, M., et al. (2019). Genomic and phenotypic delineation of congenital microcephaly. Genet. Med. *21*, 545–552. https://doi.org/10.1038/s41436-018-0140-3.
12. Sobreira, N., Schiettecatte, F., Valle, D., and Hamosh, A. (2015). GeneMatcher: a matching tool for connecting investigators with an interest in the same gene. Hum. Mutat. *36*, 928–930. https://doi.org/10.1002/humu.22844.
13. Jauss, R.-T., Popp, B., Platzer, K., and Jamra, R. (2022). MorbidGenes-Panel-v2022-02.1 (Zenodo).
14. Richards, S., Aziz, N., Bale, S., Bick, D., Das, S., Gastier-Foster, J., Grody, W.W., Hegde, M., Lyon, E., Spector, E., et al. (2015). Standards and guidelines for the interpretation of sequence variants: a joint consensus recommendation of the American college of medical genetics and genomics and the association for molecular pathology. Genet. Med. *17*, 405–424. https://doi.org/10.1038/gim.2015.30.
15. Karczewski, K.J., Francioli, L.C., Tiao, G., Cummings, B.B., Alföldi, J., Wang, Q., Collins, R.L., Laricchia, K.M., Ganna, A., Birnbaum, D.P., et al. (2020). The mutational constraint spectrum quantified from variation in 141, 456 humans. Nature *581*, 434–443. https://doi.org/10.1038/s41586-020-2308-7.
16. Lonsdale, J., Thomas, J., Salvatore, M., Phillips, R., Lo, E., Shad, S., Hasz, R., Walters, G., Garcia, F., Young, N., et al. (2013). The Genotype-Tissue expression (GTEx) project. Nat. Genet. *45*, 580–585. https://doi.org/10.1038/ng.2653.
17. Morales, J., Pujar, S., Loveland, J.E., Astashyn, A., Bennett, R., Berry, A., Cox, E., Davidson, C., Ermolaeva, O., Farrell, C.M., et al. (2022). A joint NCBI and EMBL-EBI transcript set for clinical genomics and research. Nature *604*, 310–315. https://doi.org/10.1038/s41586-022-04558-8.
18. Rentzsch, P., Schubach, M., Shendure, J., and Kircher, M. (2021). CADD-Splice—improving genome-wide variant effect prediction using deep learning-derived splice scores. Genome Med. *13*, 31. https://doi.org/10.1186/s13073-021-00835-9.
19. Jaganathan, K., Kyriazopoulou Panagiotopoulou, S., McRae, J.F., Darbandi, S.F., Knowles, D., Li, Y.I., Kosmicki, J.A., Arbelaez, J., Cui, W., Schwartz, G.B., et al. (2019). Predicting splicing from primary sequence with deep learning. Cell *176*, 535–548.e24. https://doi.org/10.1016/j.cell.2018.12.015.
20. Yeo, G., and Burge, C.B. (2004). Maximum entropy modeling of short sequence motifs with applications to RNA splicing signals. J. Comput. Biol. *11*, 377–394. https://doi.org/10.1089/1066527041410418.
21. Jian, X., Boerwinkle, E., and Liu, X. (2014). In silico prediction of splice-altering single nucleotide variants in the human genome. Nucleic Acids Res. *42*, 13534–13544. https://doi.org/10.1093/nar/gku1206.
22. Ioannidis, N.M., Rothstein, J.H., Pejaver, V., Middha, S., McDonnell, S.K., Baheti, S., Musolf, A., Li, Q., Holzinger, E., Karyadi, D., et al. (2016). REVEL: an ensemble method for predicting the pathogenicity of rare missense variants. Am. J. Hum. Genet. *99*, 877–885. https://doi.org/10.1016/j.ajhg.2016.08.016.
23. Schwarz, J.M., Rödelsperger, C., Schuelke, M., and Seelow, D. (2010). MutationTaster evaluates disease-causing potential of

sequence alterations. Nat. Methods *7*, 575–576. https://doi.org/10.1038/nmeth0810-575.

24. Jagadeesh, K.A., Wenger, A.M., Berger, M.J., Guturu, H., Stenson, P.D., Cooper, D.N., Bernstein, J.A., and Bejerano, G. (2016). M-CAP eliminates a majority of variants of uncertain significance in clinical exomes at high sensitivity. Nat. Genet. *48*, 1581–1586. https://doi.org/10.1038/ng.3703.
25. Adzhubei, I., Jordan, D.M., and Sunyaev, S.R. (2013). Predicting functional effect of human missense mutations using PolyPhen-2. Curr. Protoc. Hum. Genet.. Chapter 7, Unit7.20.
26. Altschul, S.F., Madden, T.L., Schäffer, A.A., Zhang, J., Zhang, Z., Miller, W., and Lipman, D.J. (1997). Gapped BLAST and PSI-BLAST: a new generation of protein database search programs. Nucleic Acids Res. *25*, 3389–3402. https://doi.org/10.1093/nar/25.17.3389.
27. Guex, N., and Peitsch, M.C. (1997). SWISS-MODEL and the Swiss-PdbViewer: an environment for comparative protein modeling. Electrophoresis *18*, 2714–2723. https://doi.org/10.1002/elps.1150181505.
28. Sayle, R.A., and Milner-White, E.J. (1995). RASMOL: biomolecular graphics for all. Trends Biochem. Sci. *20*, 374–376. https://doi.org/10.1016/s0968-0004(00)89080-5.
29. Wallace, A.C., Laskowski, R.A., and Thornton, J.M. (1995). LIGPLOT: a program to generate schematic diagrams of protein-ligand interactions. Protein Eng. Des. Sel. *8*, 127–134. https://doi.org/10.1093/protein/8.2.127.
30. Stiernagle, T. (2006). Maintenance of C. elegans (WormBook).
31. Barlow, I. Bleach Synchronisation of *C. elegans* v1. https://doi.org/10.17504/protocols.io.2bzgap6.
32. Islam, P. Preparing Worms for the COPAS (Wormsorter) v1. https://doi.org/10.17504/protocols.io.bfqbjmsn.
33. Moore, S., and Barlow, I. COPAS Wormsorter v1. https://doi.org/10.17504/protocols.io.bfc9jiz6.
34. Barlow, I. Disease Model Screen Protocol v1. https://doi.org/10.17504/protocols.io.bsicncaw.
35. O'Brien, J., and T.. (2022). Response of Disease Model Mutants to Cholinergic Drugs v1. https://doi.org/10.17504/protocols.io.b5p7q5rn.
36. Barlow, I., Feriani, L., Minga, E., McDermott-Rouse, A., O'Brien, T., Liu, Z., Hofbauer, M., Stowers, J.R., Andersen, E.C., Ding, S.S., et al. (2021). Megapixel Camera Arrays for High-Resolution Animal Tracking in Multiwell Plates (Animal Behavior and Cognition).
37. Javer, A., Currie, M., Lee, C.W., Hokanson, J., Li, K., Martineau, C.N., Yemini, E., Grundy, L.J., Li, C., Ch'ng, Q., et al. (2018). An open-source platform for analyzing and sharing worm-behavior data. Nat. Methods *15*, 645–646. https://doi.org/10.1038/s41592-018-0112-1.
38. Javer, A., Ripoll-Sánchez, L., and Brown, A.E.X. (2018). Powerful and interpretable behavioural features for quantitative phenotyping of *Caenorhabditis elegans*. Phil. Trans. R. Soc. B *373*, 20170375. https://doi.org/10.1098/rstb.2017.0375.
39. Benjamini, Y., and Hochberg, Y. (1995). Controlling the false discovery rate: a practical and powerful approach to multiple testing. J. Roy. Stat. Soc. B *57*, 289–300. https://doi.org/10.1111/j.2517-6161.1995.tb02031.x.
40. J O'Brien, T. (2022). Pharyngeal Pumping Assay v1. https://doi.org/10.17504/protocols.io.b3hiqj4e.
41. Kwan, P., Arzimanoglou, A., Berg, A.T., Brodie, M.J., Allen Hauser, W., Mathern, G., Moshé, S.L., Perucca, E., Wiebe, S., and French, J. (2009). Definition of drug resistant epilepsy: consensus proposal by the ad hoc Task Force of the ILAE Commission on Therapeutic Strategies. Epilepsia *51*, 1069–1077. https://doi.org/10.1111/j.1528-1167.2009.02397.x.
42. Lewis, B.P., Green, R.E., and Brenner, S.E. (2003). Evidence for the widespread coupling of alternative splicing and nonsense-mediated mRNA decay in humans. Proc. Natl. Acad. Sci. USA *100*, 189–192. https://doi.org/10.1073/pnas.0136770100.
43. Anna, A., and Monika, G. (2018). Splicing mutations in human genetic disorders: examples, detection, and confirmation. J. Appl. Genet. *59*, 253–268. https://doi.org/10.1007/s13353-018-0444-7.
44. Baralle, D. (2005). Splicing in action: assessing disease causing sequence changes. J. Med. Genet. *42*, 737–748. https://doi.org/10.1136/jmg.2004.029538.
45. Williams, S.N., Locke, C.J., Braden, A.L., Caldwell, K.A., and Caldwell, G.A. (2004). Epileptic-like convulsions associated with LIS-1 in the cytoskeletal control of neurotransmitter signaling in Caenorhabditis elegans. Hum. Mol. Genet. *13*, 2043–2059. https://doi.org/10.1093/hmg/ddh209.
46. Miller, K.G., Alfonso, A., Nguyen, M., Crowell, J.A., Johnson, C.D., Rand, J.B., and Alfonso, A. (1996). A genetic selection for Caenorhabditis elegans synaptic transmission mutants. Proc. Natl. Acad. Sci. USA *93*, 12593–12598. https://doi.org/10.1073/pnas.93.22.12593.
47. Waggoner, L.E., Dickinson, K.A., Poole, D.S., Tabuse, Y., Miwa, J., and Schafer, W.R. (2000). Long-term nicotine adaptation in *Caenorhabditis elegans* Involves PKC-dependent changes in nicotinic receptor abundance. J. Neurosci. *20*, 8802–8811. https://doi.org/10.1523/jneurosci.20-23-08802.2000.
48. Schultz, J., Bork, P., Ponting, C.P., and Hofmann, K. (1997). SAM as a protein interaction domain involved in developmental regulation: emergence of SAM. Protein Sci. *6*, 249–253. https://doi.org/10.1002/pro.5560060128.
49. Gonçalves, F.G., Caschera, L., Teixeira, S.R., Viaene, A.N., Pinelli, L., Mankad, K., Alves, C.A.P.F., Ortiz-Gonzalez, X.R., Andronikou, S., and Vossough, A. (2020). Intracranial calcifications in childhood: Part 1. Pediatr. Radiol. *50*, 1424–1447. https://doi.org/10.1007/s00247-020-04721-1.
50. Livingston, J.H., Stivaros, S., Warren, D., and Crow, Y.J. (2014). Intracranial calcification in childhood: a review of aetiologies and recognizable phenotypes. Dev. Med. Child Neurol. *56*, 612–626. https://doi.org/10.1111/dmcn.12359.
51. Duncan, C.J.A., Thompson, B.J., Chen, R., Rice, G.I., Gothe, F., Young, D.F., Lovell, S.C., Shuttleworth, V.G., Brocklebank, V., Corner, B., et al. (2019). Severe type I interferonopathy and unrestrained interferon signaling due to a homozygous germline mutation in *STAT2*. Sci. Immunol. *4*, eaav7501. https://doi.org/10.1126/sciimmunol.aav7501.
52. Meuwissen, M.E.C., Schot, R., Buta, S., Oudesluijs, G., Tinschert, S., Speer, S.D., Li, Z., van Unen, L., Heijsman, D., Goldmann, T., et al. (2016). Human USP18 deficiency underlies type 1 interferonopathy leading to severe pseudo-TORCH syndrome. J. Exp. Med. *213*, 1163–1174. https://doi.org/10.1084/jem.20151529.
53. O'Driscoll, M.C., Daly, S.B., Urquhart, J.E., Black, G.C.M., Pilz, D.T., Brockmann, K., McEntagart, M., Abdel-Salam, G., Zaki, M., Wolf, N.I., et al. (2010). Recessive mutations in the gene encoding the tight junction protein occludin cause band-like calcification with Simplified gyration and polymicrogyria. Am. J. Hum. Genet. *87*, 354–364. https://doi.org/10.1016/j.ajhg.2010.07.012.
54. Cerebral Calcification International Study Group, Tonduti, D., Panteghini, C., Pichiecchio, A., Decio, A., Carecchio,

M., Reale, C., Moroni, I., Nardocci, N., Garcia-Cazorla, A., et al. (2018). Encephalopathies with intracranial calcification in children: clinical and genetic characterization. Orphanet J. Rare Dis. *13*, 135. https://doi.org/10.1186/s13023-018-0854-y.

55. Wong, M.Y., Liu, C., Wang, S.S.H., Roquas, A.C.F., Fowler, S.C., and Kaeser, P.S. (2018). Liprin-α3 controls vesicle docking and exocytosis at the active zone of hippocampal synapses. Proc. Natl. Acad. Sci. USA *115*, 2234–2239. https://doi.org/10.1073/pnas.1719012115.
56. Kittelmann, M., Hegermann, J., Goncharov, A., Taru, H., Ellisman, M.H., Richmond, J.E., Jin, Y., and Eimer, S. (2013). Liprin-α/SYD-2 determines the size of dense projections in presynaptic active zones in C. elegans. J. Cell Biol. *203*, 849–863. https://doi.org/10.1083/jcb.201302022.
57. Patel, M.R., Lehrman, E.K., Poon, V.Y., Crump, J.G., Zhen, M., Bargmann, C.I., and Shen, K. (2006). Hierarchical assembly of presynaptic components in defined C. elegans synapses. Nat. Neurosci. *9*, 1488–1498. https://doi.org/10.1038/nn1806.
58. Spangler, S.A., Schmitz, S.K., Kevenaar, J.T., de Graaff, E., de Wit, H., Demmers, J., Toonen, R.F., and Hoogenraad, C.C. (2013). Liprin-α2 promotes the presynaptic recruitment and turnover of RIM1/CASK to facilitate synaptic transmission. J. Cell Biol. *201*, 915–928. https://doi.org/10.1083/jcb.201301011.
59. de Curtis, I. (2011). Function of liprins in cell motility. Exp. Cell Res. *317*, 1–8. https://doi.org/10.1016/j.yexcr.2010.09.014.
60. Chiaretti, S., and de Curtis, I. (2016). Role of liprins in the regulation of tumor cell motility and invasion. CCDT *16*, 238–248. https://doi.org/10.2174/1568009616031602061241O3.
61. Moog, U., and Kutsche, K. (1993). In CASK Disorders, M.P. GeneReviews®, H.H. Adam, R.A. Ardinger, S.E. Pagon, L.J. Wallace, K.W.G. Bean, G.M. Mirzaa, and A. Amemiya, eds. (University of Washington, Seattle)).

The American Journal of Human Genetics, Volume *109*

Supplemental information

Bi-allelic loss-of-function variants in *PPFIBP1* cause a neurodevelopmental disorder with microcephaly, epilepsy, and periventricular calcifications

Erik Rosenhahn, Thomas J. O'Brien, Maha S. Zaki, Ina Sorge, Dagmar Wieczorek, Kevin Rostasy, Antonio Vitobello, Sophie Nambot, Fowzan S. Alkuraya, Mais O. Hashem, Amal Alhashem, Brahim Tabarki, Abdullah S. Alamri, Ayat H. Al Safar, Dalal K. Bubshait, Nada F. Alahmady, Joseph G. Gleeson, Mohamed S. Abdel-Hamid, Nicole Lesko, Sofia Ygberg, Sandrina P. Correia, Anna Wredenberg, Shahryar Alavi, Seyed M. Seyedhassani, Mahya Ebrahimi Nasab, Haytham Hussien, Tarek E.I. Omar, Ines Harzallah, Renaud Touraine, Homa Tajsharghi, Heba Morsy, Henry Houlden, Mohammad Shahrooei, Maryam Ghavideldarestani, Ghada M.H. Abdel-Salam, Annalaura Torella, Mariateresa Zanobio, Gaetano Terrone, Nicola Brunetti-Pierri, Abdolmajid Omrani, Julia Hentschel, Johannes R. Lemke, Heinrich Sticht, Rami Abou Jamra, Andre E.X. Brown, Reza Maroofian, and Konrad Platzer

Contents

Supplemental note: Case Reports 3
Individual 1 3
Individual 2 4
Individual 3-1, 3-2 and 3-3 5
Individual 4 8
Individual 5-1 and 5-2 10
Individual 6-1 and 6-2 13
Individual 7 15
Individual 8 16
Individual 9 17
Individual 10 18
Individual 11 19
Individual 12 20
Fetus (family 13) 22
Additional Case: Individual 14 22
Figure S1. 24
Figure S2. 25
Table S1 27
Table S2 28
Table S3 29
Supplemental Methods 30
Use of GTEx Expression Data 30
Supplemental sequencing methods 30
Individual 1 30
Individual 2 31
Individuals 3-1, 3-2 and 3-3 31
Individual 4 31
Individuals 5-1, 5-2, 8 and 10 32
Individuals 6-1, 6-2 and 11 32
Individual 7 33
Individual 9 33
Individual 12 33
Fetus (Family 13) 34
RNA analysis 34
Family 1 (Figure S1) 34
Family 7 (Figure S2) 35

Supplemental References 36

Supplemental note: Case Reports

Individual 1

The male individual was born to healthy consanguineous parents (first cousins) of Turkish origin. The family history was uneventful. Pregnancy was uneventful besides growth retardation and oligohydramnios. He was born at 40+3 gestational weeks with a weight of 2650 g (-1.53 SD), a length of 49 cm (-1.44 SD) and an occipitofrontal circumference (OFC) of 33 cm (-1.15 SD). After birth, neonatal icterus and a ptosis of the left eye were noticed, he slept a lot.

Mild to moderate developmental delay was diagnosed in kindergarten. He visited a school for intellectually handicapped children. Focal impaired awareness seizures started at the age of four years with an initial frequency of two seizures per month and they required therapy with Lamotrigine and Oxcarbazepine. At the time of last examination, individual 1 was 19 years old. He presented with microcephaly (52 cm [<-2.5 SD]) and normal height (166 cm [-1.44 SD]) and weight (79 kg [0.78 SD]). At the time had focal seizures every 1-2 months and still received Lamotrigine and Oxcarbazepine. He was able to write his name, to recognize numbers and read single words. He had a large nose, short philtrum and a small chin.

Brain MRI at the age of 19 years did not reveal any anomalies.

Chromosomal analysis, SNP array and panel sequencing was normal. Trio exome sequencing revealed a homozygous splice variant in *PPFIBP1* (NM_003622.4): c.1146+1G>A. Both parents were confirmed heterozygous for this variant by Sanger sequencing and were phenotypically normal.

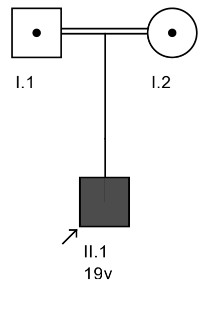

Individual 2

The female individual was born at term to unaffected and fourth degree consanguineous parents of Syrian origin. Two siblings of the individual passed away in childhood. One of them presented epilepsy and microcephaly and the second died due to high grade glioma. Neither of them was genetically tested. Pregnancy and neonatal period were uneventful.

At the age of six years, she presented with short stature (95 cm at 5 years and 4 months [-3.4 SD]), severe microcephaly (42 cm [<< -3 SD]) and low weight (12 kg [-4.14 SD]). She had profound intellectual disability (ID), absent speech, spastic tetraplegia and severely delayed motor development being unable to sit or walk independently. Seizures started at two months of age and she was lastly affected by focal as well as bilateral tonic-clonic seizures. EEG findings included a temporal paroxysmal focus with wave spikes during sleep. She received antiepileptic treatment with Levetiracetam and Clonazepam which controlled the seizures. Furthermore, she was affected by laryngo-pharyngo-tracheomalacia requiring gastrostomy at the age of five years, left hypoacusis, bilateral papillary pallor and a nystagmus on the left. Dysmorphic features included long philtrum, wide nasal bridge, low columella, synophris and low set ears. She died at the age of six years of cardiac and respiratory arrest, likely linked to intractable seizures.

Brain MRI was performed at the age of five years and revealed ventriculomegaly, leukoencephalopathy, paucity of the white matter and thalamus, microcalcification in basal ganglia, hypoplasia of the splenium and the knee of the corpus callosum and a Blake's pouch cyst. Cranial CT showed bilateral calcifications of the thalamus and periventricular along the wall of the lateral ventricles.

Trio exome sequencing revealed a homozygous frameshift variant in *PPFIBP1* (NM_003622.4): c.2654del, p.(Tyr885Leufs*4). The parents were heterozygous for this variant. In two daughters of a paternal cousin who presented ID, hypotonia and hyporeflexia with respiratory insufficiency and feeding difficulties, a homozygous mutation in *TBCK* was found. The mutation was not present in the individual described here.

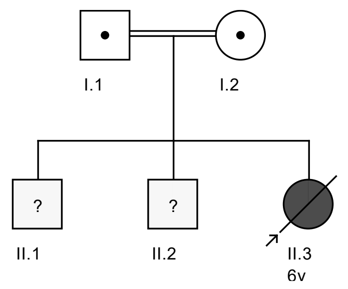

Individual 3-1, 3-2 and 3-3

Individual 3-1:

The male individual was born to healthy consanguineous parents. In the family there are two other similarly affected siblings (individuals 3-2 and 3-3) and a fourth sibling died shortly after birth at the age of 6 days and was not molecularly tested.

After an uneventful pregnancy, individual 3-1 was born at term (37+6 gestational weeks) with a weight of 2200 g (-2.67 SD), a length of 44 cm (-3.11 SD) and an OFC of 28.5 cm (-4.69 SD). After birth, two episodes of apnea were reported.

At the age of 11 years, he presented decreased body weight of 15 kg (<<-3 SD) and severe microcephaly with an OFC of 41 cm (<<-3 SD). He was affected by profound global developmental delay (GDD)/ ID, absent speech, spastic tetraplegia and severely delayed motor development being unable to sit or walk independently. He achieved social smiling at the age of 12 months. Seizures started at the age of seven months as epileptic spasms that evolved to focal, focal to bilateral tonic-clonic, and tonic seizures that occurred daily. Seizures were refractory to multiple antiepileptic drugs (Phenobarbital, Vigabatrin, Clonazepam). EEG findings included hypsarrhythmia and multifocal epileptiform discharges. Furthermore, he was noted to have impaired hearing, congenital heart defects (atrial septal defect and patent ductus arteriosus), a single kidney and café au lait spots.

CT and MRI imaging performed in the individual revealed bilateral intracranial calcifications in the periventricular region and in the centrum semiovale, periventricular leukomalacia and metopic synostosis.

Singleton exome sequencing revealed a homozygous frameshift variant in *PPFIBP1* (NM_003622.4): c.1368_1369del, p.(Glu456Aspfs*3). Exome sequencing also identified a homozygous missense variant c.5814C>A, p.(Asp1938Glu) in *CENPF* (GenBank: NM_016343.3; MIM: 600236) which is associated with Stromme syndrome (MIM: 243605) caused by bi-allelic truncating variants. Since up to date, no (likely) pathogenic missense variants were described to cause this condition and as his very similarly affected brother (Individual 3-2) was found heterozygous for this variant, it was not considered to be causative. The individual was tested positive for increased chromosomal breakage.

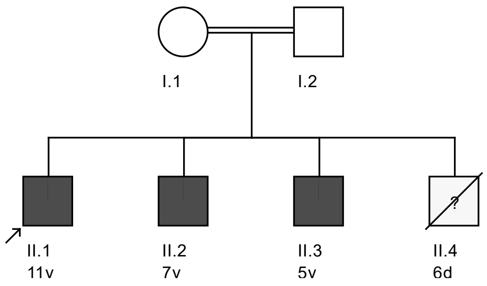

Individual 3-2:

The male individual was born to healthy consanguineous parents. In the family there are two other similarly affected siblings (individuals 3-1 and 3-3) and a fourth sibling died shortly after birth at the age of 6 days and was not molecularly tested.

After an uneventful pregnancy, individual 3-2 was born at term (37 gestational weeks) with a weight of 2200 g (-2.67 SD), a length of 46 cm (-2.05 SD) and an OFC of 31.5 cm (-2.33 SD). The neonatal period was uneventful

At the age of seven years, he presented decreased body weight of 14 kg (-3.84 SD) and severe microcephaly with an OFC of 44 cm (<<-3 SD). He was affected by severe GDD/ID, absent speech, spastic tetraplegia and severely delayed motor development being unable to sit or walk independently. He achieved social smiling at the age of 10 months. Seizures started at the age of two to three months as epileptic spasms. Epilepsy evolved to Lennox-Gastaut syndrome in the course. Seizures were very frequent and refractory to multiple antiepileptic drugs (Levetiracetam, Topiramate, Vigabatrin, Clonazepam). EEG showed hypsarrhythmia and in the course Lennox-Gastaut syndrome pattern.

Furthermore, he was noted to have impaired hearing, congenital heart defects (atrial septal defect, patent ductus arteriosus, small ventricular septal defect) and undescended testes.

MRI performed at the age of 3 days and 2 years showed some punctual hyperintensities in the basal ganglia progressing to moderate hyperintensities in the periventricular white matter and mild ventriculomegaly. CT scan revealed bilateral intracranial calcifications in the periventricular region and in the centrum semiovale,

Singleton exome sequencing revealed a homozygous frameshift variant in *PPFIBP1* (NM_003622.4): c.1368_1369del, p.(Glu456Aspfs*3).

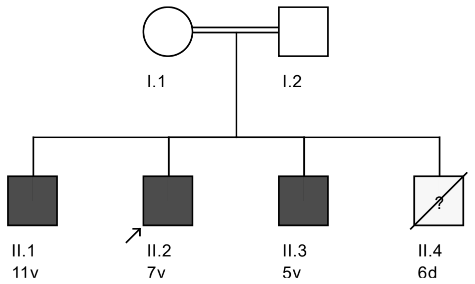

Individual 3-3:

The male individual was born to healthy consanguineous parents. In the family there are two other similarly affected siblings (individuals 3-1 and 3-2) and a fourth sibling died shortly after birth at the age of 6 days and was not molecularly tested.

Individual 3-3 was born late pre-term (36+3 gestational weeks) with a weight of 1800 g (-2.42 SD), a length of 43 cm (-1.92 SD) and an OFC of 29.5cm (-2.33 SD). During pregnancy intrauterine growth retardation was noted.

At the age of five years he presented decreased body weight of 8.5 kg (-5.28 SD) and severe microcephaly with an OFC of 39.5 cm (<< -3 SD). He was affected by severe GDD/ID, absent speech, spastic tetraplegia and severely delayed motor development being unable to sit or walk independently. He achieved social smile at the age of 12 months. Seizures started at the first day of life and evolved from left focal seizures to epileptic spasms and multifocal epilepsy. Seizures were refractory to multiple antiepileptic drugs (Levetiracetam, Phenobarbital, Vigabatrin). EEG showed right hemispheric mainly

temporal epileptiform discharges. Furthermore, he was noted to have impaired hearing, congenital heart defects (atrial septal defect, patent ductus arteriosus), undescended testes and a right ectopic pelvic kidney.

MRI from the third day of life showed ventriculomegaly with irregular walls of the lateral ventricles, T1-hyperintensity and T2-hypoinenstity in the periventricular white matter and in the basal ganglia, abnormal signal intensity of the white matter on the right side, and bilateral temporal and left occipital thickening of the cortex (pachygyria). CT scan revealed bilateral calcifications in the periventricular white matter, the ventricles wall and in basal ganglia.

Singleton exome sequencing revealed a homozygous frameshift variant in *PPFIBP1* (NM_003622.4): c.1368_1369del, p.(Glu456Aspfs*3). Exome sequencing also identified a homozygous missense variant c.5814C>A, p.(Asp1938Glu) in *CENPF* (GenBank: NM_016343.3; MIM: 600236) which is associated with Stromme syndrome (MIM: 243605) caused by bi-allelic truncating variants. Since up to date, no (likely) pathogenic missense variants were described to cause this condition and as his very similarly affected brother (Individual 3-2) was found heterozygous for this variant, it was not considered to be causative.

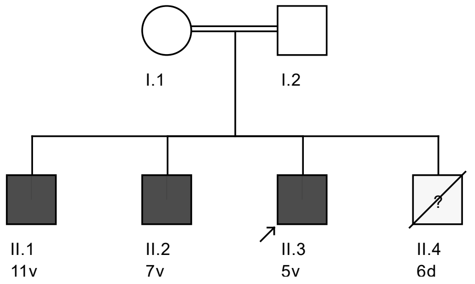

Individual 4

The male individual was born via normal vaginal delivery after 40 weeks of pregnancy as the first child of healthy consanguineous first-degree-cousin parents. The mother had a vaginal infection during pregnancy. Intrauterine growth restriction was evident in the prenatal ultrasound. At birth, his bodyweight was 2110 g (-2.92 SD), his length was 45 cm (-2.58 SD), his head circumference was 30 cm (-3.51 SD) and Apgar score was 7 and 9 at 1 and 5 minutes, respectively. He stayed in NICU for one

month with the impression of a congenital cytomegalovirus (CMV) infection based on the clinical presentation of microcephaly, intracranial calcifications, haemorrhagic retinitis, neonatal hypoglycaemia, neonatal seizure, hyperbilirubinemia and congenital heart disease with VSD of 5 mm, small PDA, small ASD and dilated left ventricle (LV) and left atrium (LA) with left to right shunt. CMV IgG serology was positive, but urine CMV PCR was negative. He received an intravenous course of Ganciclovir.

After discharge from NICU, he continued to have severe global developmental delay and recurrent admissions to the hospital. Chronic retinal detachment was found at 5 months of age. Also, he developed different types of seizures including focal onset seizures, tonic seizures and infantile spasms. His antiepileptic drugs included Phenobarbitone, Levetiracetam, Vigabatrin and Topiramate.

Currently, he can make cooing sounds and shows some interest in lights. On the most recent examination at 11 months of age, his head circumference was 39 cm (<< -3 SD), his weight was 8.5 kg (-0.93 SD) and his length was 67 cm (-2.94 SD). He had dysmorphic features including microcephaly, bitemporal hollowing, broad nasal bridge, long philtrum, micrognathia, short neck, low set ears, prominent antihelix ears, and bilateral undescended testes. He had right eye leukocoria, right eye exotropia with slow pupillary reaction on the same eye. His gag reflex was fair. He had spontaneous antigravity movements of his limbs, hypertonia with spasticity of his elbows, wrists, hips and knees, hyperreflexia with spreading and crossing and positive clonus.

Singleton exome sequencing revealed a homozygous frameshift variant in *PPFIBP1* (NM_003622.4): c.1368_1369del, p.(Glu456Aspfs*3). The parents were phenotypically normal and confirmed as heterozygous carriers of the variant.

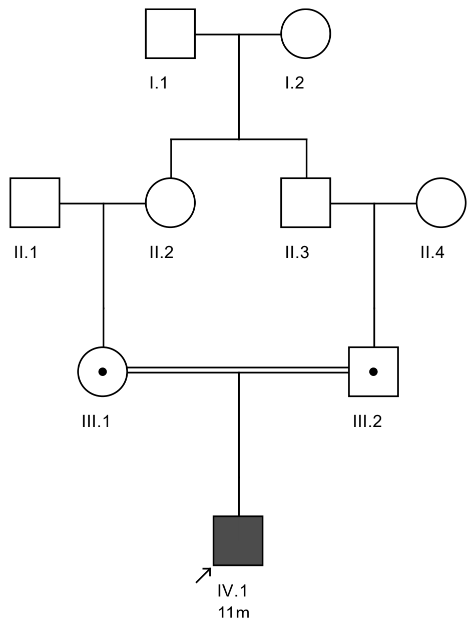

Individual 5-1 and 5-2

Individual 5-1:

The female individual was born to unaffected consanguineous (second degree) parents of Egyptian origin. She has a similarly affected sibling (see individual 5-2). Pregnancy was uneventful and she was born at term (39 gestational weeks) with a weight of 2500 g (-1.73 SD), length of 46 cm (-1.69 SD) and microcephaly with an OFC of 30 cm (-3.27 SD). After birth she presented with mild cyanosis and weak crying.

At the last assessment at the age of eight years she presented short stature (97 cm [-5 SD]), decreased weight (18 kg [-2.24 SD]) and severe progressive microcephaly (OFC = 42,5 cm [<< -3 SD]). She was affected by profound ID, absent speech, spastic tetraplegia and was unable to sit or walk. Seizures started at the age of two months, initially occurred daily and included focal seizures with head deviation and myoclonic epilepsy as clusters. By treatment with multiple antiepileptic drugs (VPA, CNZ, CBZ, LEV, LTG) seizures could be fairly controlled but seizures always recurred again in the course. EEG showed bilateral temporal paroxysmal discharges. Further symptoms included acquired scoliosis from spasticity

and a deglutition disorder with feeding difficulties, failure to thrive and infrequent vomiting. As a dysmorphic feature, bitemporal hollowing was noted. She died at the age of eight years.

Cranial CT showed periventricular calcifications with a linear pattern surrounding the lateral ventricles. Brain MRI was not performed.

TORCH screening, extended Metabolic screening and karyotyping were normal. Quadruple exome sequencing revealed a homozygous nonsense variant in *PPFIBP1* (NM_003622.4): c.2413C>T, p.(Arg805*) that was also detected in the similarly affected sibling in homozygous state. Her parents were phenotypically normal and found to be heterozygous carriers of the variant.

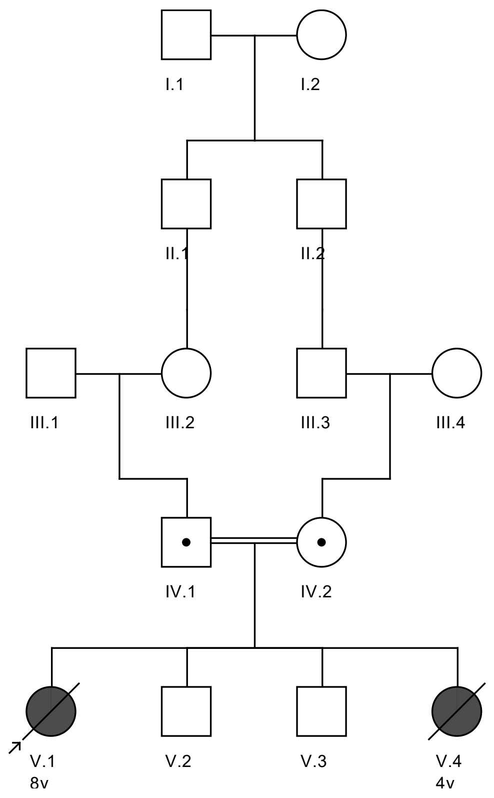

Individual 5-2:

The female individual was born to unaffected consanguineous (second degree) parents of Egyptian origin. Apart from a peptic ulcer in the mother, pregnancy was uneventful and she was born at term (38 gestational weeks) with a weight of 2700 g (-1.23 SD), a length of 47 cm (-1.15 SD) and microcephaly with an OFC of 30.5 cm (-2.85 SD).

At the last assessment at the age of two years she presented short stature (77 cm [-2.71 SD]), decreased body weight (9.5 kg [-1.57 SD]) and severe progressive microcephaly (OFC = 39.5 cm [<< -3 SD]). She was affected by profound ID, absent speech, hypertonia of the limbs, dystonia and was unable to sit or walk. Seizures started at the age of one month, initially as daily focal seizures with versive head movement that, after one month, evolved to myoclonic, focal and sometimes tonic seizures partly associated with cyanosis. She was treated with multiple antiepileptic drugs (VPA, CBZ, LEV, LTG) but seizures were refractory. EEG showed active left centro-temporal epileptogenic discharges. She experienced feeding difficulties. As dysmorphic features bitemporal hollowing, an upturned nose, a long philtrum and low set ears were noted. She died at the age of four years.

Cranial CT showed periventricular calcifications with a linear pattern surrounding the lateral ventricles and an enlarged ventricular system. Brain MRI was not performed.

TORCH screening was normal. Quadruple exome sequencing revealed a homozygous nonsense variant in *PPFIBP1* (NM_003622.4): c.2413C>T, p.(Arg805*) that was also detected in the similarly affected sibling in homozygous state. Her parents were phenotypically normal and found to be heterozygous carriers of the variant.

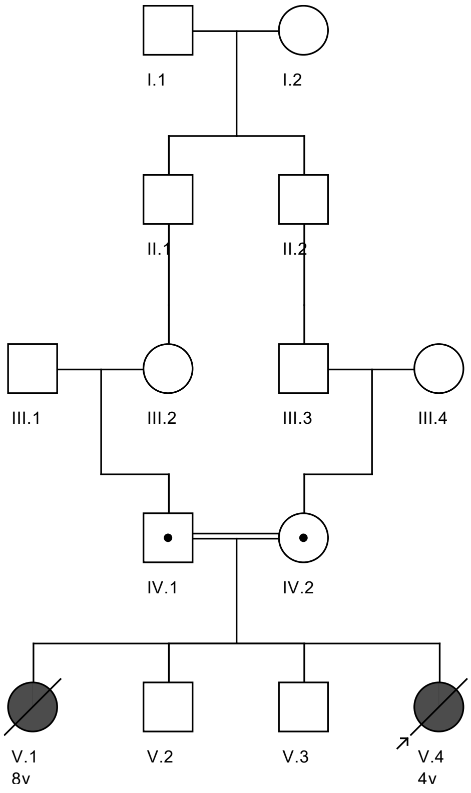

Individual 6-1 and 6-2

Individual 6-1:

The male individual was born to healthy first-cousin parents of Egyptian origin. He has a similarly affected younger brother (individual 6-2) and an unaffected brother. The pregnancy was uneventful and he was born at term (40 gestational weeks) with a weight of 2900 g (-0.96 SD), a length of 49 cm (-0.47 SD) and with an OFC of 32 cm (-1.94 SD).

At the last assessment at the age of six years he presented short stature (103 cm [-2.63 SD]), decreased body weight (15.3 kg [-2.3 SD]) and severe progressive microcephaly (OFC = 42.2 cm [<< -3 SD]). He was affected by profound ID, absent speech, hypertonia of the limbs and was unable to walk but could sit independently at the age of 6 years. Seizures started at the age of 4 months as myoclonic, followed by generalized tonic-clonic seizures and attacks of cyanosis, especially occurring together with fever. He was treated with multiple antiepileptic drugs (VPA, LEV, CZP) and the seizures could be fairly controlled and reduced to about one per month. EEG showed multifocal epileptiform discharges with abnormal background activity. Ophthalmological examination revealed optic atrophy and he was only

able to follow light. He experienced feeding difficulties was unable to masticate. As dysmorphic features a long face, prominent supraorbital ridges, sparse eyebrows, a prominent and upturned nose, a long philtrum, a v shaped upper lip, low set large ears with prominent antihelix and a retruded mandible were noted.

Brain MRI at the age of 4 years showed mild ventriculomegaly, cortical atrophy, a deep Sylvian fissure, periventricular and deep white matter demyelination mainly around frontal and occipital horn, a thin corpus callosum, and cerebellar vermian hypoplasia.

Metabolic screening and karyotyping were normal. Singleton exome sequencing revealed a homozygous nonsense variant in *PPFIBP1* (NM_003622.4): c.1468C>T, p.(Gln490*) that was also detected in the similarly affected sibling in homozygous state. His parents were phenotypically normal and heterozygous for this variant by Sanger sequencing.

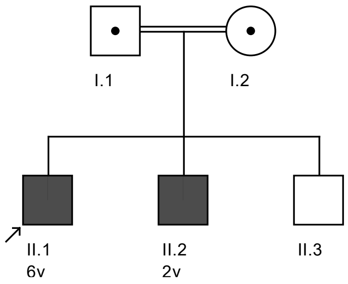

Individual 6-2:

The male individual was born to healthy first-cousin parents of Egyptian origin. He has a similarly affected older brother and an unaffected younger brother. The pregnancy was uneventful and he was born at term (39 GW) with a weight of 2800 g (-1.18 SD), a length of 47 cm (-1.52 SD) and microcephaly with an OFC of 31.8 cm (-2.10 SD). After birth he was admitted to hospital with jaundice for phototherapy.

At the last assessment at the age of two years he presented with short stature (79 cm [-2.66 SD]), decreased body weight (9.1 kg [-2.54 SD]) and severe progressive microcephaly (OFC = 40 cm [<< -3 SD]). He was affected by profound GDD with absent speech and no head support. He showed high spasticity, rigidity and dystonic movements. Seizures started at the age of two months and included

myoclonic, generalized tonic-clonic and tonic seizures with upward gaze and he showed excessive smacking movements. He was treated with multiple antiepileptic drugs (VPA, LEV, CZP) but seizures were refractory. EEG showed active multifocal spikes more in right temporoparietal area. Ophthalmological examination revealed optic atrophy and he was not able to follow light. Further symptoms included feeding difficulties and a persistent ductus arteriosus which was operated at one year of age. As dysmorphic features a long face, a high forehead, prominent supraorbital ridge, sparse eyebrows, a prominent and upturned nose, a long philtrum, a V-shaped upper lip, low set large ears with a prominent antihelix, retruded mandible and chin dimple were noted.

Brain MRI at the age of 8 months showed an asymmetric dilatation of the lateral ventricles with more dilatation on the right side, cortical atrophy, deep Sylvian fissures, periventricular and deep white matter demyelination mainly around frontal and occipital horn, a thin corpus callosum and cerebellar vermian hypoplasia.

Karyotyping, metabolic screening and TORCH screening were normal. Singleton exome sequencing revealed a homozygous nonsense variant in *PPFIBP1* (NM_003622.4): c.1468C>T, p.(Gln490*) that was also detected in the similarly affected sibling in homozygous state. His parents were phenotypically normal and heterozygous for this variant by Sanger sequencing.

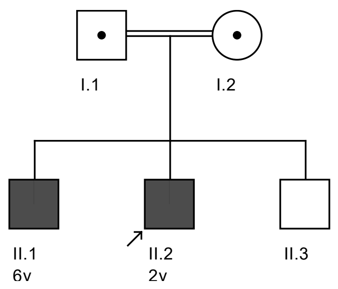

Individual 7

Individual 7 is the first common child of parents from the same area in northern Sweden. She was born full term, and already from the start she gave less contact and appeared hypotonic as compared to other children. Around 4 months of age she started having apneic episodes, and EEG confirmed epileptic activity. Her seizures progressed into infantile spasms at 7 months of age. She was treated with ACTH,

but the spasms recurred and Vigabatrin and Topiramate were added. An initial MRI performed at 4 months of age was normal. She continued to have multiple seizure types including myoclonias. At 1,5 years of age Valproic acid was added which controlled the seizures well. Concomitantly, a new MRI at 1,5 years of age showed a thin corpus callosum, patchy dysmyelination in periventricular regions, and possibly a reduction of the white matter. Analysis of amino acids, pipecolic acid, and very long chain fatty acids in plasma were all normal, as were organic acids, purine and pyrimidine metabolites and amino acids in urine. In her lumbar puncture she had increased levels of Glial Fibrillary Acidic Protein (GFAP) and Neurofilament light (NfL), indicating active destruction. Her head circumference was normal at birth, but at 2 years of age clearly microcephalic. Her development was now at 2 years of age delayed. She was not spastic, but she did have lively reflexes. She had a wide based gait and she could pick up objects using her full hand. She did not have any language and little understanding. She did recognize her close family but paid little attention to the milieu around her. Muscle biopsy showed slightly reduced ATP production with several substrates. She is now 4 years old, still seizure free on treatment, and with little development as compared to 2 years of age. Trio genome sequencing identified a homozygous mutation in the *PPFIBP1* gene c.403C>T, p.(Arg135*). Her parents were both confirmed heterozygous for the variant by sanger sequencing.

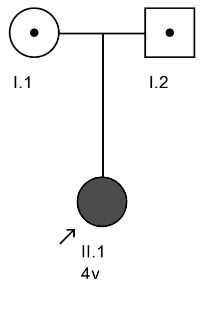

Individual 8

The female individual was born to unaffected consanguineous parents of Iranian origin. In the pedigree, a maternal aunt with developmental delay, seizures and congenital heart defects was described who deceased and was not molecularly evaluated. During pregnancy, the mother was affected by a urinary tract infection, a renal stone and headache. Individual 8 was born at 37+6 gestational weeks with a weight of 2600 g (-1.47 SD), a length of 50 cm (0.46 SD) and an OFC of 33 cm (-0.74 SD). As she had

a congenital coronary fistula, mitral regurgitation and cardiomegaly, she underwent angiography for closure of the fistula during neonatal period.

At the age of three years she presented with normal height (98 cm [0.77 SD]), a body weight of 11 kg (-1.85 SD) and progressive low head circumference (-1.78 SD, 3.8th percentile). She was affected by profound developmental delay, was not able to sit independently, did not speak and showed muscular hypotonia and nystagmus. Epilepsy started at the age of six months and she was affected by generalized tonic-clonic seizures. Antiepileptic treatment with topiramate, valproate and levetiracetam controlled the seizures and she was seizure free for 5 months. Further symptoms included mild scoliosis and an internal rotation of the right foot. MRI of the head at the age of six months revealed a severe disorder of gyration with bilateral parietal thickening of the cortex and pachygyria, severe gray matter heterotopia along the lateral ventricles wall, ventriculomegaly with irregular walls, and hyperintensity and paucity of the white matter

Singleton exome sequencing revealed a homozygous nonsense variant in *PPFIBP1* (NM_003622.4): c.1417_1427del, p.(Ala473Lysfs*20) that was found heterozygous in the parents and in the unaffected brother by Sanger sequencing.

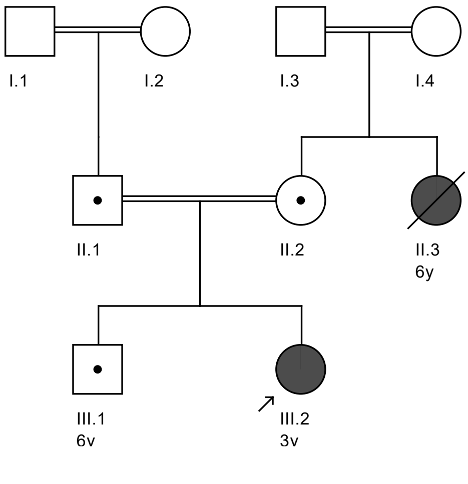

Individual 9

The male individual was born to unaffected parents. In the pedigree, low vision, disability due to seizures, congenital heart disease and dwarfism have been reported. Pregnancy was uneventful except for the umbilical cord being wrapped around the fetus' neck leading to preterm labour. He was born at

38 gestational weeks with a weight of 1780g (-3.79 SD), length of 40 cm (-5.22 SD) and microcephaly with an OFC of 31 cm (-2.73 SD).

At the last assessment at the age of 2 years and 6 months, he presented with severe ID, absent speech, spastic tetraplegia with no sphincter control and blindness. He was able to sit independently after occupational therapy, but unable to walk. Seizures occurred daily and started at the age of two months as gaze deviation and epileptic spasms and later frequently affected vision. He was treated with CZP and VPA and he was seizure free for a few months. He died at the age of 3.8 years.

Singleton exome sequencing revealed a homozygous nonsense variant in *PPFIBP1* (NM_003622.4): c.1300C>T, p.(Gln434*). Exome sequencing also identified a heterozygous suspicious variant in *ACADS* (MIM: 606885) but so far, only bi-allelic variants were described to cause ACADS deficiency (MIM: 201470) and the variant thus was not deemed causative for his condition. His parents were phenotypically normal.

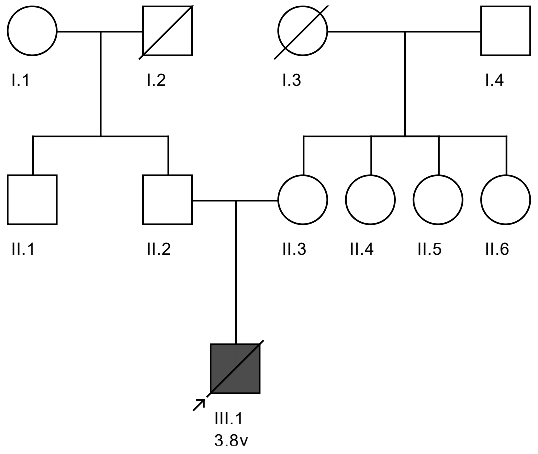

Individual 10

The male individual was born to healthy consanguineous parents of Egyptian origin. A similarly affected sister has passed away before. He was born at term (38 GW) with a weight of 3000 g (-0.73 SD) and microcephaly. He was admitted to neonatal intensive care unit as febrile convulsion started in the first week of life. In the course, epilepsy manifested with daily focal myoclonic seizures and epileptic spasms and hypsarrhythmia was seen on EEG. Seizures were controlled by medication with Valproate and Diazepam, although seizures relapsed once, but could be re-controlled with add-on medication. At the

age of one year and two months he presented with severe global developmental delay. At that time, he did not show social smile, was not able to sit independently and did not vocalise. Furthermore, he was affected by muscular hypotonia, right eye ptosis, left nystagmus, left iris coloboma and diffuse chorioretinal degeneration. MRI revealed bilateral symmetrical supratentorial abnormal myelination in the periventricular white matter, corona radiata and centrum semiovale, as well as a hypoplastic corpus callosum and a mildly ectatic ventricular system.

Singleton exome sequencing revealed a homozygous nonsense variant c.2629C>T, p.(Arg877*) in *PPFIBP1* (NM_003622.4). The parents were phenotypically normal and heterozygous by Sanger sequencing.

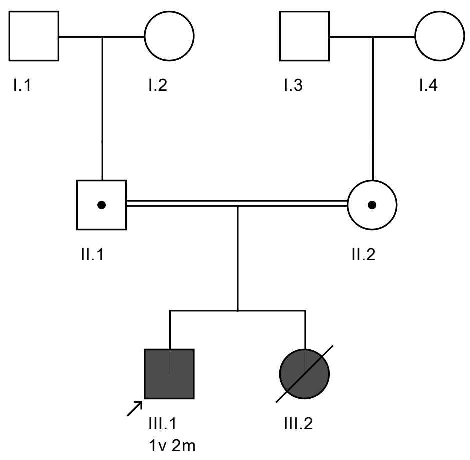

Individual 11

The male patient is the only child of healthy consanguineous Egyptian parents. The parents are first cousins. He was born at term by CS after an otherwise uneventful pregnancy with a birth weight of 2500 g (-1.9 SD, 3rd percentile). No documents were available for the birth length, OFC and Apgar score. First focal seizures started at age 2 weeks which initially were well-controlled by levetiracetam. At the age of 5 months, seizures progressed into myoclonic jerks five to seven times a day and clonazepam was started and later valproate was added. Despite these multiple antiepileptic drugs, his seizures were partially controlled on the maximum dosing of these drugs He was referred to the clinical genetics department at that age for genetic counselling. On physical examination, the weight was 8.3 kg (0.91 SD), length was 65 cm (-0.43 SD), and head circumference was 38 cm (-3.77 SD). Dysmorphic features

included bitemporal hollowing, wide nasal ridge, antiverted nostrils, low set ears and short neck. The patient showed global developmental delay; he could not recognize his parents, had no head control and did not show focusing, vocalizing, or smiling.

Neurologic examination demonstrated head lag, hypotonia, brisk reflexes, intermittent dystonic posture and nystagmus. Ophthalmologic examination showed bilateral primary optic atrophy. EEG showed occasional runs of spike slow waves. CT scans showed calcifications in the internal capsule, basal ganglia, white matter and periventricular. Brain-MRI displayed cortical atrophy, deep Sylvian fissures, mild ventriculomegaly, prominent basal ganglia, a hypoplastic corpus callosum, and retrocerebellar and bitemporal arachnoid cysts.

Singleton exome sequencing revealed a homozygous nonsense variant c.1468C>T, p.(Gln490*) in *PPFIBP1* (NM_003622.4). The parents were tested heterozygous for the variant by Sanger sequencing.

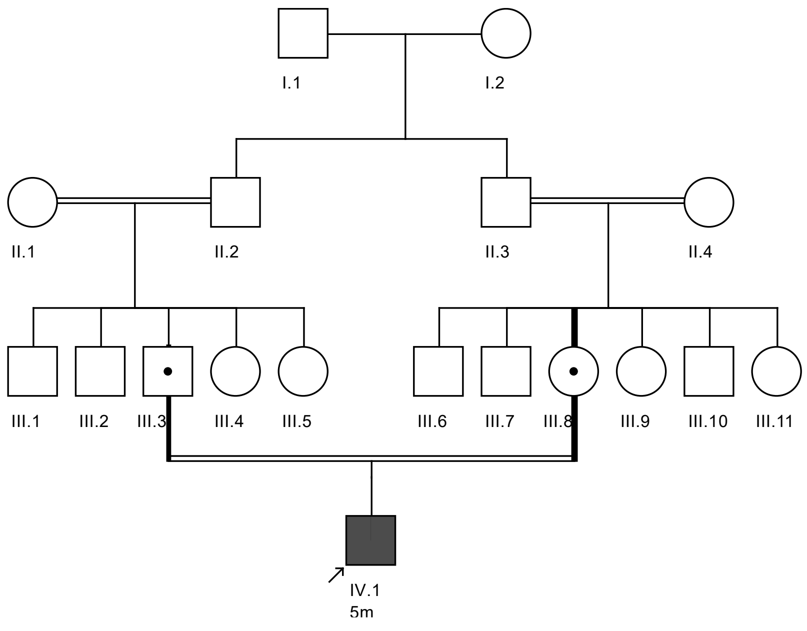

Individual 12

The female individual (V.I) was born at term after an uneventful pregnancy to unaffected and second-degree cousins of Syrian origin. At birth respiratory distress was reported but no mechanical ventilation was needed. From infancy, she was noted to have poor sucking with feeding difficulties, truncal hypotonia, and severe developmental delay. At 6 months, she developed generalized and focal seizures.

She became seizure-free with polytherapy including phenobarbital, valproic acid and carbamazepine. EEG during sleep showed slowing of background activity on the left posterior regions and epileptiform abnormalities on bi-hemispheric anterior regions (left> right). At her first evaluation at the age of 3 years and 9 months, her weight was 12.9 Kg (-1.4 SD), her length 90 cm (-2.6 SD) and her occipitofrontal circumference was 38.5 cm (-7.5 SD). She was noted to have dysmorphic features including sloping forehead, low-set hairline, and synophris. At the age of 5 years and 11 months, she presented with short stature (96 cm [-3.67 SD]), severe microcephaly (42 cm [-8.5 SD]) and low weight (13.2 kg [-3.1 SD]). She had profound intellectual disability with absent speech, truncal hypotonia being unable to sit or walk independently, dyskinetic movements and frequent stereotypic movements (e.g., hand-to-mouth).

A brain MRI performed at the age of five years revealed leukoencephalopathy with paucity of the white matter, ventriculomegaly with irregular walls of the lateral ventricles and suspected periventricular microcalcifications. It also showed abnormalities of cortical gyration including bilateral frontal polymicrogyria and thickening of the temporoparietal cortex, especially of the right insula.

Trio exome sequencing revealed a homozygous frameshift variant in *PPFIBP1* (NM_003622.4): c.2654del, p.(Tyr885Leufs*4). Her parents (IV.2; III.4) were phenotypically normal and heterozygous for this variant. Sanger sequencing showed the heterozygous variant only in one of the healthy sisters (V.3). One daughter (IV.1) of the maternal grandfather (III.2) suffered from severe encephalopathy.

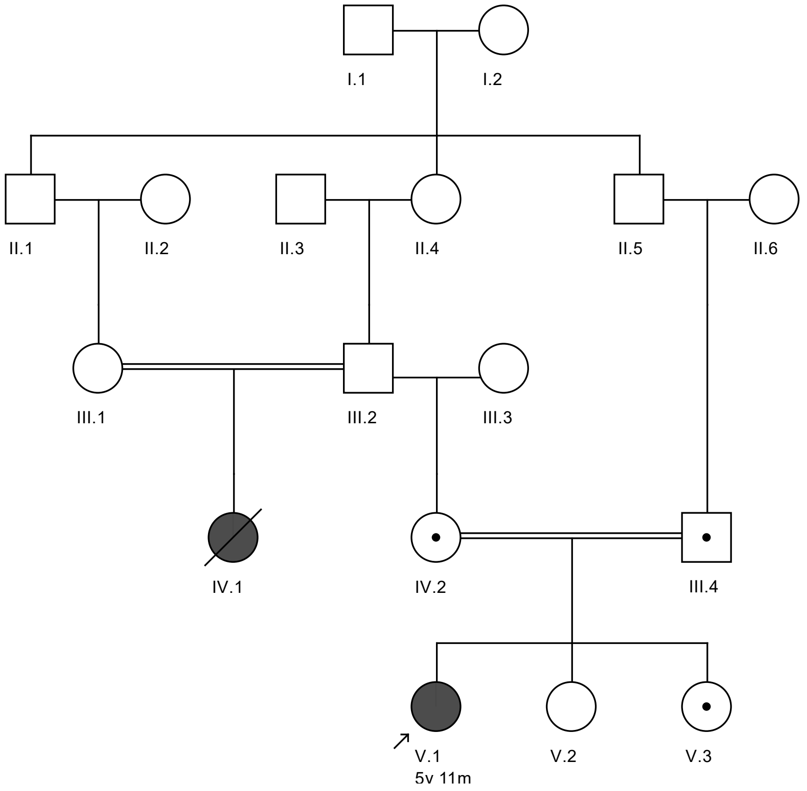

Fetus (family 13)

The clinical and molecular findings in the fetus are fully described in the main article.

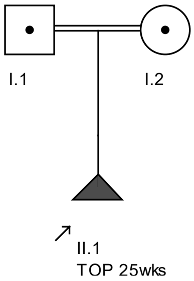

TOP = termination of pregnancy 1

Additional Case: Individual 14

In a four and a half year old male individual, a homozygous splice-site variant chr12:g.27682500T>C, NM_003622.4:c.2158+2T>C, p.? was identified by exome sequencing. The variant is predicted by splice AI to result in a disruption of the 3'-donor splice site of exon 23/30. Skipping of this exon would be out of frame. The variant is absent from gnomAD.

Exome sequencing also found a homozygous missense variant in the *WWOX* gene (MIM: 605131): NM_016373.4:c.900C>A, p.(Asn300Lys). This variant is absent from gnomAD and has a CADD score of 19.26. Bi-allelic missense variants in WWOX have been associated with autosomal recessive Spinocerebellar ataxia (MIM:614322) and bi-allelic LoF variants have been associated with developmental and epileptic encephalopathy (MIM:616211). Likely pathogenic and pathogenic bi-allelic missense variants have been reported in ClinVar. The *WWOX* variant has been classified as a variant of unknown significance (VUS) according to the guidelines of the ACMG.

The individual was born preterm at 32 gestational weeks due to a fetal heart rhythm disorder with a weight of 2000 g (0.59 SD) and an occipitofrontal circumference of 30.2 cm (0.55 SD). He later was affected by global developmental delay including delayed walking and sitting. At the age of 4 months, he developed refractory epilepsy including clonic seizures, upward gaze and epileptic spasms. Additionally, he showed strabismus and bilateral visual defects.

Given the VUS in the *WWOX* gene, two possible genetic diagnoses are plausible for this individual. To prevent this possibility from impacting the phenotypic description this Individual was not included in the overall phenotypic and genetic description.

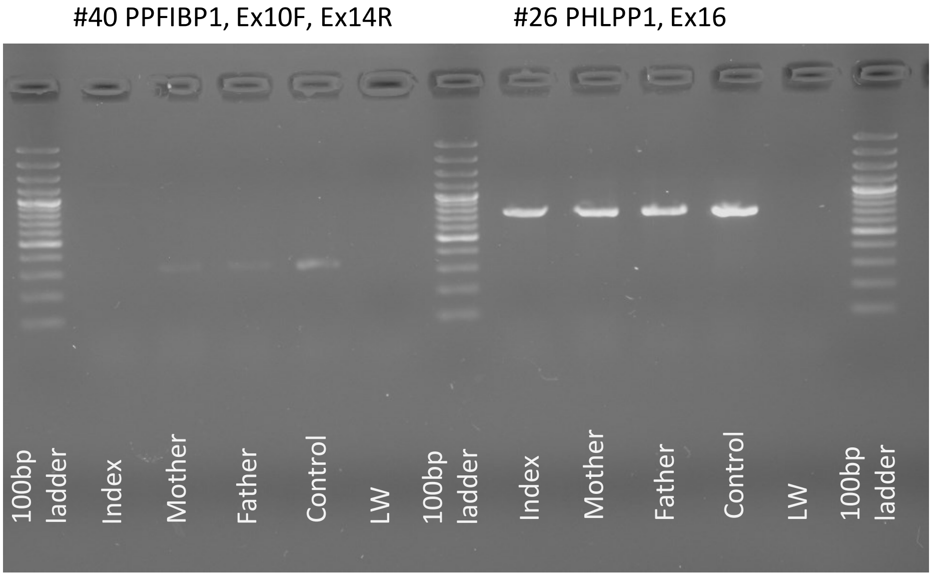

Figure S1. Gel electrophoresis of cDNA from individual 1, his parents, and a control sample of *PPFIBP1* (left) and a reference gene (*PHLPP1*, Ex 16; right). For the region *PPFIBP1*, spanning Ex10F-Ex14R encompassing the variant NM_003622.4: c.1146+1G>A, weaker bands compared to the control can be seen in the heterozygous parents and no band is seen in the homozygous index. This observation supports nonsense-mediated mRNA decay (NMD) of the mutated allele. For the reference gene, the bands have similar intensities in all samples.

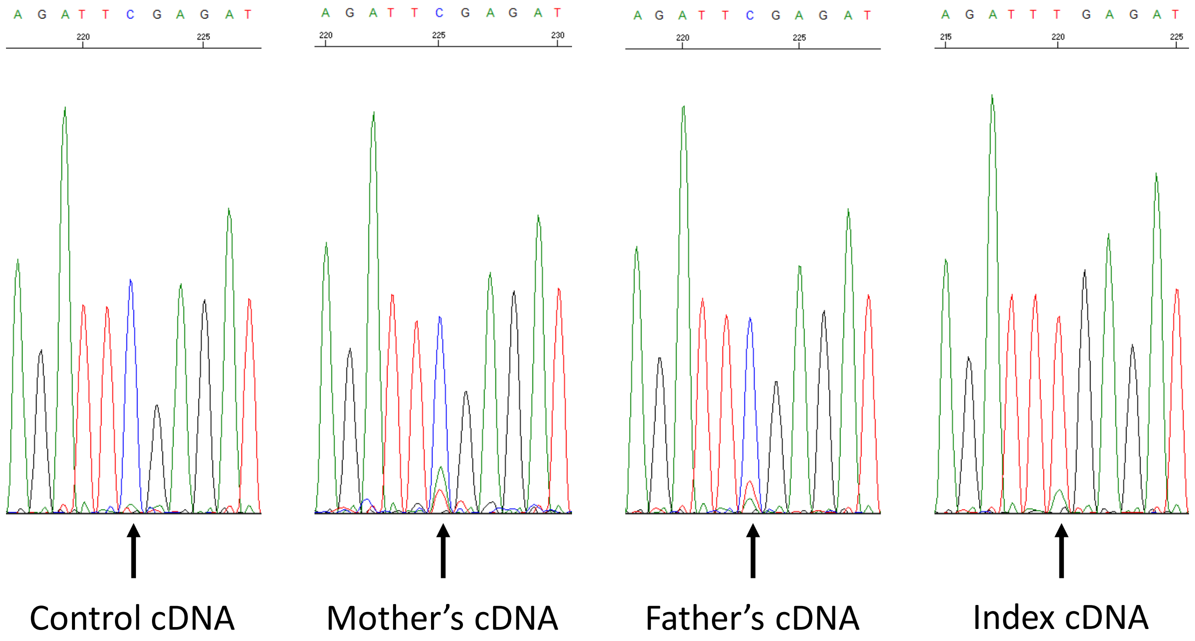

Figure S2. Electropherogram showing the sequence surrounding the c.403C>T variant at the cDNA level from a control sample, the parents who are heterozygous for the variant and individual 7 who is homozygous for the variant. The arrow indicates the base that is changed at the genomic level. Both parents show reduced signals of the mutated allele compared to the wild-type allele which suggests NMD of the aberrant mRNA.

Table S1

Individual	Chr	Genomic position (GRCh38)	cDNA (NM_003622.4)	Protein (NP_003613.4)	Allelic state	Predicted effect	ACMG criteria	Classification
1	12	g.27667321G>A	c.1146+1G>A	p.?	homozygous	Loss of 5'-donor splice site of exon 13	PVS1, PM2_Supporting, PM3_Supporting	Pathogenic
2, 12	12	g.27689172del	c.2654del	p.(Tyr885Leufs*4)	homozygous	Nonsense mediated mRNA decay	PVS1, PM2_Supporting, PM3_Supporting	Pathogenic
3-1, 3-2, 3-3, 4	12	g.27673815_27673816del	c.1368_1369del	p.(Glu456Aspfs*3)	homozygous	Nonsense mediated mRNA decay	PVS1, PM2_Supporting, PM3_Supporting, PP1_Moderate	Pathogenic
5-1, 5-2	12	g.27688340C>T	c.2413C>T	p.(Arg805*)	homozygous	Nonsense mediated mRNA decay	PVS1, PM2_Supporting, PM3_Supporting	Pathogenic
6-1, 6-2, 11	12	g.27676485C>T	c.1468C>T	p.(Gln490*)	homozygous	Nonsense mediated mRNA decay	PVS1, PM2_Supporting, PM3_Supporting	Pathogenic
7	12	g.27647774C>T	c.403C>T	p.(Arg135*)	homozygous	Nonsense mediated mRNA decay	PVS1, PM2_Supporting, PM3_Supporting	Pathogenic
8	12	g.27676434_27676444del	c.1417_1427del	p.(Ala473Lysfs*20)	homozygous	Nonsense mediated mRNA decay	PVS1, PM2_Supporting, PM3_Supporting	Pathogenic
9	12	g.27672464C>T	c.1300C>T	p.(Gln434*)	homozygous	Nonsense mediated mRNA decay or inframe deletion of exon 15	PVS1_Moderate, PM2_Supporting, PM3_Supporting	Uncertain significance**

**Table S1 continued**								
10	12	g.27689147C>T	c.2629C>T	p.(Arg877*)	homozygous	Nonsense mediated mRNA decay	PVS1, PM2_Supporting, PM3_Supporting	Pathogenic
Fetus (13)	12	g.27682633G>T	c.2177G>T	p.(Gly726Val)	homozygous	Missense change predicted to severely disturb topology of the SAM-domain region	PM2_Supporting PM3_Supporting PP3	Uncertain significance ***
Additional case (14)	12	g.27682500T>C	c.2158+2T>C	p.?	homozygous	Loss of 5'-donor splice site of exon 23	PVS1 PM2_Supporting, PM3_Supporting	Pathogenic

* Although ACMG criteria are formally not perfectly suited for the description of novel disease-associated genes, we classified all variants from this study to simplify further use of the data.

** Since SpliceAI predicts a loss of the acceptor and donor splice sites of exon 15/30 with moderate probabilities, an in-frame deletion of exon 15/30 removing less than 10% of the protein cannot be ruled out. Thus, the PVS1 criterion is weighted at a moderate level and this variant can only be classified as of uncertain significance. Due to the high clinical overlap to the rest of the cohort, this variant is deemed causative despite being classified as uncertain.

***Due to the high clinical overlap to the rest of the cohort, this variant is deemed causative despite being classified as uncertain.

Table S1. Variant information and classification according to the ACMG criteria[1,*]. Variant descriptions were validated according to the HGVS nomenclature.

**Ind.**	**Genomic position** (GRCh38)	**cDNA**	**CADD-v6**[2]	**SpliceAI**[3]	**MaxEntScan**[4]	**NNSPLICE**[5]	**Nucleotide conservation**	**gnomAD**[6]
1	chr12:g.27667321	c.1146+1G>A, p.?	35	0.95	1	1	High	0

Table S2. *In silico* prediction of the splice variant *PPFIBP1* (NM_003622.4). Red color represents a very high probability of the variant to be damaging for each aspect in the table.

**Ind.**	**Genomic position** (GRCh38)	**cDNA**	**CADD-v6**[2]	**REVEL**[7]	**Mutation Taster**[8]	**M-CAP 1.3**[9]	**Polyphen 2 v2.2.2**[10]	**GERP++**[11]	**AA conservation**	**gnomAD**[6]
Fetus (11)	chr12:27682633	c.2177G>T, p.(Gly726Val)	24.6	0,609 (LDC)	D	0.063 (PoP)	PrD	4,46	High	0

Table S3. *In silico* prediction of the missense variant and conservation of the affected amino acid in *PPFIBP1*(NM_003622.4). Red color represents a very high probability of the variant to be damaging for each aspect in the table; LDC = likely disease causing; D = Deleterious; PoP = Possibly Pathogenic; PrD = Probably Damaging

Supplemental Methods

Use of GTEx Expression Data

To assess the relevance of the variants in different transcripts by using the expression data of GTEx v8 featuring Ensembl transcripts, the following assumptions have been made given the only minor differences in the untranslated regions of the transcripts. It has been assumed that NM_003622.4 which equals the Ensembl transcript ENST00000228425.11 also equals the version ENST00000228425.10. Also, it has been assumed that the transcript NM_001198915.2 equals ENST00000537927.5.

Supplemental sequencing methods

Individual 1

Trio exome sequencing of the index and the parents was performed at the Institute of Human Genetics at the University of Leipzig Medical Center. Library preparation was done using the Nextera DNA Flex Pre-Enrichment LibraryPrep with Illumina Nextera DNA UD Indexes by Illumina (San Diego, CA, USA). Target enrichment was achieved by using the Human Core Exome hybridization probes from Twist Bioscience (San Francisco, CA, USA). Paired-end Next-Generation-Sequencing was then performed on a NovaSeq 6000 Instrument (Illumina), located at the facilities of GeneWIZ (Leipzig, Germany), using an S1 Reagent Kit (300 cycles) by Illumina. Coverage of more than 20x has been achieved in more than 95 % of target sequences in all family members.

Analysis of the raw data, variant annotation and prioritization were performed using the software Varfeed (Limbus, Rostock, Germany) and Varvis (Limbus, Rostock, Germany). Variants were prioritized based on the mode of inheritance, impact on the gene product, minor allele frequency and *in silico* predicted pathogenicity. For research evaluation of variants in potential candidate genes, we also considered the biological function of the gene product with regards to neurodevelopment, mutational constraint parameters[6] (i.e. observed/expected - ratio, pLI-Score, Z-Score), expression patterns of the gene[14], insights on animal models and further aspects such as protein interaction networks and the function of similar or related proteins.

Individual 2

Trio exome sequencing of the index case and the parents was performed as previously described.[15] Library preparation was done using the Nextera DNA Flex Pre-Enrichment LibraryPrep with Illumina Nextera DNA UD Indexes by Illumina (San Diego, CA, USA). Target enrichment was achieved by using a modified version of the Human Core Exome hybridization probes from Twist Bioscience (San Francisco, CA, USA) complemented with additional custom probes. Paired-end Next-Generation-Sequencing (2x100 bp) was then performed on a NovaSeq 6000 Instrument (Illumina), at IntegraGen SA (Evry, France). Coverage of more than 20X has been achieved in more than 97 % of target sequences in the index case. Analysis of the raw data, variant annotation and prioritization were performed as previously described.[15]

Individuals 3-1, 3-2 and 3-3

Exome sequencing and variant prioritization supported by autozygome analysis were performed as previously described.[16,17]

Individual 4

Exome sequencing of the index and targeted sequencing on both DNA strands of the relevant *PPFIBP1* region for the parents was performed at CENTOGENE (the rare disease company) (Rostock, Germany). Double stranded DNA capture baits against approximately 36.5 Mb of the human coding exome (targeting >98% of the coding RefSeq from the human genome build GRCh37/hg19) are used to enrich target regions from fragmented genomic DNA with the Twist Human Core Exome Plus kit by Twist Bioscience (San Francisco, CA, USA). The generated library is sequenced on an Illumina platform (San Diego, CA, USA) to obtain at least 20x coverage depth for >98% of the targeted bases. An in-house bioinformatics pipeline, including read alignment to GRCh37/hg19 genome assembly, variant calling, annotation and comprehensive variant filtering is applied. All variants with minor allele frequency

(MAF) of less than 1% in gnomAD[6] database, and disease-causing variants reported in HGMD[18], in ClinVar[19] or in CentoMD® by CENTOGENE are considered. The investigation for relevant variants is focused on coding exons and flanking +/-20 intronic nucleotides of genes with clear gene-phenotype evidence (based on OMIM® information). All potential modes of inheritance patterns are considered. In addition, provided family history and clinical information are used to evaluate identified variants with respect to their pathogenicity and causality. Variants are categorized into five classes (pathogenic; likely pathogenic; VUS; likely benign; benign). All variants related to the phenotype of the patient are reported. CENTOGENE has established stringent quality criteria and validation processes for variants detected by next-generation sequencing. Variants with low quality and/or unclear zygosity are confirmed by orthogonal methods. Consequently, a specificity of >99.9% for all reported variants is warranted.

Individuals 5-1, 5-2, 8 and 10

Exome sequencing and variant annotation were performed as previously described.[20]

Individuals 6-1, 6-2 and 11

Exome sequencing (ES) of the proband was performed using a TruSeq DNA PCR-free library preparation method by Illumina (San Diego, CA, USA) and subsequent sequencing on a NovaSeq 6000 sequencing instrument (Illumina) to a sequencing depth of 30x median coverage. The resulting ES data was analysed using the Mutation Identification Pipeline (MIP) as previously described.[21] Segregation of the variant identified in *PPFIBP1* was carried out by PCR amplification of genomic DNA and subsequent Sanger sequencing using the BigDye version 3.1 sequencing kit (Applied Biosystems, Foster City, CA, USA) on a 3500 Genetic Analyzer (Applied Biosystems).

Individual 7

Genome sequencing of the proband and her parents was performed at the Science for Life Laboratory, Clinical Genomics facility, Stockholm, using a TruSeq DNA PCR-free library preparation method by Illumina (San Diego, CA, USA) and subsequent sequencing on a NovaSeq 6000 sequencing instrument (Illumina) to a sequencing depth of 30x median coverage. The resulting WGS data was analyzed using the Mutation Identification Pipeline (MIP) as previously described.[21] Confirmation of the variant observed in *PPFIBP1* was carried out by PCR amplification of genomic DNA and subsequent Sanger sequencing using the BigDye version 3.1 sequencing kit (Applied Biosystems, Waltham, Massachusetts, USA) on a 3500xl Genetic Analyzer (Applied Biosystems).

Individual 9

Exome sequencing of the patient was performed at the Macrogen company (Seoul, South Korea) using Agilent SureSelect V6 post capture kit with a NovaSeq 6000 instrument by Illumina (San Diego, CA, USA). Target regions have a mean read depth of 100X.

Analysis of the raw data was performed using BWA and GATK packages. Variant annotation and prioritization were done using an in-house pipeline of the Palindrome lab; briefly this pipeline uses an ACMG based algorithm and an in-house database for variant prioritization. Clinically significant variants are curated manually.

Individual 12

Genomic DNA was extracted from peripheral blood leukocytes using standard protocols. For library preparation we followed the manufacturer's instructions (SureSelectQXT Automated Target Enrichment for the Illumina Platform, Protocol Version B0, November 2015, Agilent Technologies, Santa Clara, CA, USA). Target enrichment for trio exome sequencing was achieved by using the SureSelect Human All Exon v7 (Agilent Technologies, Santa Clara, CA, USA). The libraries were sequenced using the NovaSeq 6000 system performing paired-end runs covering at least 2x150nt.

(Illumina Inc., San Diego, CA, USA). The generated sequences were analyzed using an in-house pipeline designed to automate the analysis workflow. The average exome coverage of the target bases of at least 100X with 90% of the bases covered by at least 40 reads. Direct Sanger Sequencing using specific primers was performed using the BigDye version 3.1 sequencing kit (Applied Biosystems, Waltham, Massachusetts, USA) on a 3500xl Genetic Analyzer (Applied Biosystems).

Fetus (Family 13)

Trio exome sequencing of the index and the parents was performed at Laboratoire Biomnis-Eurofins (Lyon, France). Library preparation was done using the Twist Library Preparation from Twist Bioscience (San Francisco, CA, USA). Target enrichment was achieved by using the Twist Human Core Exome hybridization probes from Twist Bioscience. Paired-end Next-Generation-Sequencing was then performed on a NextSeq500 Instrument, using an a NextSeq 500/550 High Output Kit v2 (2x75pb, paired-end) by Illumina (San Diego, CA, USA). Coverage of more than 30x has been achieved in more than 97 % of target sequences in all family members.

Analysis of the raw data, variant annotation and prioritization were performed using the software SeqOne (Montpellier, France).

Sanger verification has been performed with the BigDye v3.1 kit followed by a purification with the BigDye X-terminator from Life Technologies. The electrophoresis has been done on the SeqStudio instrument from Applied Biosystems (Waltham, Massachusetts, USA).

RNA analysis

Family 1 (Figure S1)

RNA was extracted using PAXgene Blood RNA Kit (Qiagen) with an input volume of 9 ml. cDNA synthesis was done using PrimeScript RT Master Mix (TaKaRa) starting with a total RNA amount of 1 µg. PCR was done using DreamTaq Hot Start DNA Polymerase (ThermoScientific) and the following oligos: PPFIBP1 (NM_001198915.2), Ex10_A_F, 5’ GCTGCTCAATTCCAGTTCCA 3’; Ex14_A_R,

5’ TGCTGGACTTCTGCAGACTT 3’ generating a 332 bp PCR product and as a reference PHLPP1 (NM_194449.3), Ex16_A_F, GGAATGTGGAGGTGCCCTAC, Ex16_A_R, gggaggaaagatgccaggac, generating a 713 bp product. Sanger sequencing was performed using PCR product after clean-up using Exo SAP-IT Express (Applied Biosystems) and the oligos as described above. Sequencing reaction was done using Big Dye Terminator v3.1 Cycle Sequencing kit and Big Dye Terminator v1.1, v3.1 5x Sequencing Buffer. Final clean up was done using ethanol. Electrophoresis was performed on a ABI3500 capillary electrophoresis instrument (Thermo Scientific).

Family 7 (Figure S2)

RT-PCR was performed on RNA isolated from the patient and parents blood, using the High Capacity cDNA Reverse Transcription Kit (Applied Biosystems, Thermo Fisher) and the following M13-tagged primers: PPFIBP1_cDNA_4F:TGGATTGTTAGAGATGATGG and PPFIBP1_cDNA_8R:GAAGTCTCTCACTGTCCATT. Subsequent sequencing of the PCR products was carried out with M13 primers, using the BigDye version 3.1 sequencing kit (Applied Biosystems) on a 3500xl Genetic Analyzer (Applied Biosystems) with alignment to the reference sequence NM_003622.

Supplemental References

1. Richards, S., Aziz, N., Bale, S., Bick, D., Das, S., Gastier-Foster, J., Grody, W.W., Hegde, M., Lyon, E., Spector, E., et al. (2015). Standards and guidelines for the interpretation of sequence variants: a joint consensus recommendation of the American College of Medical Genetics and Genomics and the Association for Molecular Pathology. Genetics in Medicine *17*, 405–424.

2. Rentzsch, P., Schubach, M., Shendure, J., and Kircher, M. (2021). CADD-Splice—improving genome-wide variant effect prediction using deep learning-derived splice scores. Genome Med *13*, 31.

3. Jaganathan, K., Kyriazopoulou Panagiotopoulou, S., McRae, J.F., Darbandi, S.F., Knowles, D., Li, Y.I., Kosmicki, J.A., Arbelaez, J., Cui, W., Schwartz, G.B., et al. (2019). Predicting Splicing from Primary Sequence with Deep Learning. Cell *176*, 535-548.e24.

4. Yeo, G., and Burge, C.B. (2004). Maximum Entropy Modeling of Short Sequence Motifs with Applications to RNA Splicing Signals. Journal of Computational Biology *11*, 377–394.

5. Jian, X., Boerwinkle, E., and Liu, X. (2014). In silico prediction of splice-altering single nucleotide variants in the human genome. Nucleic Acids Research *42*, 13534–13544.

6. Karczewski, K.J., Francioli, L.C., Tiao, G., Cummings, B.B., Alföldi, J., Wang, Q., Collins, R.L., Laricchia, K.M., Ganna, A., Birnbaum, D.P., et al. (2020). The mutational constraint spectrum quantified from variation in 141,456 humans. Nature *581*, 434–443.

7. Ioannidis, N.M., Rothstein, J.H., Pejaver, V., Middha, S., McDonnell, S.K., Baheti, S., Musolf, A., Li, Q., Holzinger, E., Karyadi, D., et al. (2016). REVEL: An Ensemble Method for Predicting the Pathogenicity of Rare Missense Variants. The American Journal of Human Genetics *99*, 877–885.

8. Schwarz, J.M., Rödelsperger, C., Schuelke, M., and Seelow, D. (2010). MutationTaster evaluates disease-causing potential of sequence alterations. Nat Methods *7*, 575–576.

9. Jagadeesh, K.A., Wenger, A.M., Berger, M.J., Guturu, H., Stenson, P.D., Cooper, D.N., Bernstein, J.A., and Bejerano, G. (2016). M-CAP eliminates a majority of variants of uncertain significance in clinical exomes at high sensitivity. Nat Genet *48*, 1581–1586.

10. Adzhubei, I., Jordan, D.M., and Sunyaev, S.R. (2013). Predicting functional effect of human missense mutations using PolyPhen-2. Curr Protoc Hum Genet *Chapter 7*, Unit7.20.

11. Cooper, G.M., Stone, E.A., Asimenos, G., NISC Comparative Sequencing Program, Green, E.D., Batzoglou, S., and Sidow, A. (2005). Distribution and intensity of constraint in mammalian genomic sequence. Genome Res *15*, 901–913.

12. Altschul, S.F., Madden, T.L., Schäffer, A.A., Zhang, J., Zhang, Z., Miller, W., and Lipman, D.J. (1997). Gapped BLAST and PSI-BLAST: a new generation of protein database search programs. Nucleic Acids Res *25*, 3389–3402.

13. Altschul, S.F., Wootton, J.C., Gertz, E.M., Agarwala, R., Morgulis, A., Schäffer, A.A., and Yu, Y.-K. (2005). Protein database searches using compositionally adjusted substitution matrices. FEBS J *272*, 5101–5109.

14. Lonsdale, J., Thomas, J., Salvatore, M., Phillips, R., Lo, E., Shad, S., Hasz, R., Walters, G., Garcia, F., Young, N., et al. (2013). The Genotype-Tissue Expression (GTEx) project. Nat Genet *45*, 580–585.

15. Tran Mau-Them, F., Duffourd, Y., Vitobello, A., Bruel, A., Denommé-Pichon, A., Nambot, S., Delanne, J., Moutton, S., Sorlin, A., Orphanomix Physician's Group, et al. (2021). Interest of exome

sequencing trio-like strategy based on pooled parental DNA for diagnosis and translational research in rare diseases. Molec Gen & Gen Med *9*,.

16. Shaheen, R., Maddirevula, S., Ewida, N., Alsahli, S., Abdel-Salam, G.M.H., Zaki, M.S., Tala, S.A., Alhashem, A., Softah, A., Al-Owain, M., et al. (2019). Genomic and phenotypic delineation of congenital microcephaly. Genetics in Medicine *21*, 545–552.

17. Saudi Mendeliome Group (2015). Comprehensive gene panels provide advantages over clinical exome sequencing for Mendelian diseases. Genome Biol *16*, 134.

18. Stenson, P.D., Mort, M., Ball, E.V., Chapman, M., Evans, K., Azevedo, L., Hayden, M., Heywood, S., Millar, D.S., Phillips, A.D., et al. (2020). The Human Gene Mutation Database (HGMD®): optimizing its use in a clinical diagnostic or research setting. Hum Genet *139*, 1197–1207.

19. Landrum, M.J., Lee, J.M., Benson, M., Brown, G.R., Chao, C., Chitipiralla, S., Gu, B., Hart, J., Hoffman, D., Jang, W., et al. (2018). ClinVar: improving access to variant interpretations and supporting evidence. Nucleic Acids Research *46*, D1062–D1067.

20. Makrythanasis, P., Maroofian, R., Stray-Pedersen, A., Musaev, D., Zaki, M.S., Mahmoud, I.G., Selim, L., Elbadawy, A., Jhangiani, S.N., Coban Akdemir, Z.H., et al. (2018). Biallelic variants in KIF14 cause intellectual disability with microcephaly. Eur J Hum Genet *26*, 330–339.

21. Stranneheim, H., Lagerstedt-Robinson, K., Magnusson, M., Kvarnung, M., Nilsson, D., Lesko, N., Engvall, M., Anderlid, B.-M., Arnell, H., Johansson, C.B., et al. (2021). Integration of whole genome sequencing into a healthcare setting: high diagnostic rates across multiple clinical entities in 3219 rare disease patients. Genome Med *13*, 40.

22. Mencacci, N.E., Kamsteeg, E.-J., Nakashima, K., R'Bibo, L., Lynch, D.S., Balint, B., Willemsen, M.A.A.P., Adams, M.E., Wiethoff, S., Suzuki, K., et al. (2016). De Novo Mutations in PDE10A Cause Childhood-Onset Chorea with Bilateral Striatal Lesions. Am J Hum Genet *98*, 763–771.
